# Insights into the diversification of subclade IVa bHLH transcription factors in Fabaceae

**DOI:** 10.1186/s12870-021-02887-w

**Published:** 2021-02-23

**Authors:** Hayato Suzuki, Hikaru Seki, Toshiya Muranaka

**Affiliations:** 1 Department of Biotechnology, Graduate School of Engineering, Osaka University, 2-1 Yamadaoka, Suita, Osaka, 565-0871 Japan; 2 Industrial Biotechnology Initiative Division, Institute for Open and Transdisciplinary Research Initiatives, Osaka University, 2-1 Yamadaoka, Suita, Osaka, 565-0871 Japan

**Keywords:** Fabaceae, Triterpene saponin, bHLH, Transcriptional regulation, Classification

## Abstract

**Background:**

Fabaceae plants appear to contain larger numbers of subclade IVa basic-helix-loop-helix (bHLH) transcription factors than other plant families, and some members of this subclade have been identified as saponin biosynthesis regulators. We aimed to systematically elucidate the diversification of this subclade and obtain insights into the evolutionary history of saponin biosynthesis regulation in Fabaceae.

**Results:**

In this study, we collected sequences of subclade IVa bHLH proteins from 40 species, including fabids and other plants, and found greater numbers of subclade IVa bHLHs in Fabaceae. We confirmed conservation of the bHLH domain, C-terminal ACT-like domain, and exon-intron organisation among almost all subclade IVa members in model legumes, supporting the results of our classification. Phylogenetic tree-based classification of subclade IVa revealed the presence of three different groups. Interestingly, most Fabaceae subclade IVa bHLHs fell into group 1, which contained all legume saponin biosynthesis regulators identified to date. These observations support the co-occurrence and Fabaceae-specific diversification of saponin biosynthesis regulators. Comparing the expression of orthologous genes in *Glycine max*, *Medicago truncatula*, and *Lotus japonicus*, orthologues of MtTSAR1 (the first identified soyasaponin biosynthesis regulatory transcription factor) were not expressed in the same tissues, suggesting that group 1 members have gained different expression patterns and contributions to saponin biosynthesis during their duplication and divergence. On the other hand, groups 2 and 3 possessed fewer members, and their phylogenetic relationships and expression patterns were highly conserved, indicating that their activities may be conserved across Fabaceae.

**Conclusions:**

This study suggests subdivision and diversification of subclade IVa bHLHs in Fabaceae plants. The results will be useful for candidate selection of unidentified saponin biosynthesis regulators. Furthermore, the functions of groups 2 and 3 members are interesting targets for clarifying the evolution of subclade IVa bHLH transcription factors in Fabaceae.

**Supplementary Information:**

The online version contains supplementary material available at 10.1186/s12870-021-02887-w.

## Background

Triterpene saponins are a group of plant specialised (secondary) metabolites found widely across the plant kingdom [1]. Triterpenes have been intensively studied in terms of their biosynthesis [2], transcriptional regulation [3–5], and bioactivities [6–8]. The monocotyledonous plants in the genus *Avena* produce antifungal saponins, known as avenacins in the roots for protection against take-all disease [9]. Saponins produced by *Barbarea vulgaris* (Brassicaceae) show antifeedant activity against insect herbivores [10]. Fabaceae (Leguminosae) plants produce structurally diverse triterpenes, including hemolytic saponins in *Medicago truncatula*, betulinic acid in *Lotus japonicus*, and glycyrrhizin in *Glycyrrhiza uralensis*, as well as a common group called soyasaponins [11–15]. Leguminous triterpenes affect symbiotic nodulation, as transgenic *M. truncatula* with elevated saponin content showed enhanced nodulation [16] and *L. japonicus* mutants lacking lupeol (the precursor of betulinic acid) showed a phenotype of rapid nodulation [17].

Basic-helix-loop-helix (bHLH) transcription factors are one of the largest families of plant transcription factors, and are classified into approximately 25 subclades based on sequence homology within the bHLH domain and other shared protein domains [18, 19]. Land plants have acquired more bHLH genes than animals, chlorophytes, or red algae [19], and some subclades evolved to regulate plant specialised metabolism [5]. Subclade IVa is a good example of such regulation, as it represents conserved transcriptional regulation of methyl jasmonate (MeJA)-mediated metabolic processes in plants [5]. TRITERPENE SAPONIN BIOSYNTHESIS ACTIVATING REGULATOR1 (MtTSAR1) upregulates the soyasaponin pathway in *M. truncatula* [20]. MtTSARs 2 and 3 are factors that activate hemolytic saponin accumulation, with differences in tissue specificity [20, 21]. We recently identified GubHLH3 as a positive regulator of soyasaponin biosynthesis in *G. uralensis* [22], and this protein is closely related to MtTSAR2 but not MtTSAR1. This finding hints at the evolutionary history of Fabaceae subclade IVa bHLHs. *Chenopodium quinoa* (Amaranthaceae) seeds accumulate saponins with similar structures to the hemolytic saponins of *M. truncatula*. Mutations in *CqTSAR-like1* (*CqTSARL1*) were identified as a major factor affecting differences in the saponin accumulation pattern between saponin-producing and saponin-lacking ecotypes [23]. In *Catharanthus roseus* (Apocynaceae), bHLH iridoid synthesis 1 (CrBIS1) and CrBIS2 were found to positively regulate the biosynthesis pathway for the iridoid branch of monoterpenoid indole alkaloids (MIAs) [24, 25]. Interestingly, the functions of MtTSARs and CrBIS1 were shown to be interchangeable through heterologous expression of MtTSARs in *C. roseus* and CrBIS1 in *M. truncatula* [26]. In addition, production of both saponins and MIAs were commonly regulated by MeJA [5, 21, 24, 27].

Numerous studies have reported genome-wide identification and classification of bHLH factors in plants [18, 19, 28–30]. Although the genomes of *Arabidopsis thaliana* and *Oryza sativa* possess four and six subclade IVa members, respectively [19], more than 30 subclade IVa bHLH genes were found in the genomes of *Glycine max* and *M. truncatula* [21, 28]. This finding suggests that Fabaceae plants may have acquired a large number of subclade IVa members during the evolution of saponin biosynthesis.

In this study, we extensively explored subclade IVa bHLHs in fabids and showed that Fabaceae plants possess a large number of subclade IVa members, which were classified into three groups based on phylogenetic analysis. Group 1 had the greatest number of members, including MtTSARs and GubHLH3. Groups 2 and 3 contained fewer members, none of which were functionally-identified, but were obviously distinct from group 1 based on the tree and highly conserved among Fabaceae plants. We also performed in silico analysis to elucidate their structures and functions. This study will help to narrow down the candidates of unidentified saponin biosynthesis regulators and clarify the evolution of subclade IVa members in Fabaceae plants.

## Results

### Large numbers of subclade IVa members in Fabaceae plants

A total of 319 bHLH proteins and 33 subclade IVa members were identified previously in *G. max* [28]. We obtained 355 sequences of *G. max* bHLH proteins (Additional file 1: Table S1) using PlantTFDB [31]. Then, we assigned individual names to the novel members and re-selected subclade IVa members based on sequence similarity of the full-length proteins. Although five proteins (GmbHLH60–64) were designated as members of subclade IVa in a previous study [28], they had relatively long amino acid sequences (588–653 aa) and clustered more closely with bHLH proteins in subclade IIIf from *A. thaliana* on the phylogenetic tree (Additional file 3: Fig. S1). GmbHLH327, 329, 331, 334, 337, and 345 were newly assigned to subclade IVa based on Basic Local Alignment Search Tool (BLAST) search results. Finally, we identified 34 *G. max* subclade IVa bHLHs ranging in peptide length from 195 to 390 aa (Additional file 1: Table S1).

We collected sequences of all bHLH proteins from 40 plant species including *A. thaliana*, *C. roseus*, *C. quinoa* and various fabids (Additional file 1: Table S2). These proteins were used as queries for BLAST searches against the 4 and 34 subclade IVa bHLHs identified in *A. thaliana* and *G. max*, and we thereby identified the subclade IVa members in each plant species (Additional file 2). Fabaceae plants possessed 61 to 355 bHLHs and 4 to 35 subclade IVa members, while species outside of Fabaceae had 94 to 250 bHLHs and 2 to 8 subclade IVa members (Table 1). Because genome sequences of *Arachis hypogaea* and *Vigna unguiculata* were not used for the prediction in PlantTFDB, their bHLH sequences may not have all been collected. The percentage of subclade IVa genes relative to all bHLH genes was 5.56–18.2% and 1.82–5.76% in Fabaceae and non-Fabaceae fabids, respectively (Table 1). The genomes of Fabaceae contained significantly more subclade IVa bHLH genes than those of related plant families (Mann–Whitney U test, *U* = 329, *p* < 10^− 9^).

**Table 1:** Numbers of total bHLH and subclade IVa genes

ID	Species	bHLH	IVa	Group 1	Group 2	Group 3	% (IVa/bHLH)
01_Cl	*Citrullus lanatus*	126	4	0	1	3	3.17
02_Cm	*Cucumis melo*	131	4	0	1	3	3.05
03_Cs	*Cucumis sativus*	130	4	0	1	3	3.08
**04_Ad**	***Arachis duranensis***	**156**	**13**	**9**	**2**	**2**	**8.33**
**05_Ah**	***Arachis hypogaea***^a^	**72**	**4**	**4**	**0**	**0**	**5.56**
**06_Ai**	***Arachis ipaensis***	**160**	**11**	**7**	**2**	**2**	**6.88**
**07_Cc**	***Cajanus cajan***	**174**	**16**	**12**	**2**	**2**	**9.20**
**08_Ca**	***Cicer arietinum***	**140**	**14**	**10**	**2**	**2**	**10.0**
**09_Gm**	***Glycine max***	**355**	**34**	**26**	**4**	**4**	**9.58**
**10_Gs**	***Glycine soja***	**342**	**35**	**29**	**2**	**4**	**10.2**
**11_Gu**	***Glycyrrhiza uralensis***^b^	**163**	**10**	**8**	**2**	**0**	**6.13**
**12_Lj**	***Lotus japonicus***	**152**	**15**	**10**	**2**	**3**	**9.87**
**13_Mt**	***Medicago truncatula***	**181**	**33**	**28**	**2**	**3**	**18.2**
**14_Pv**	***Phaseolus vulgaris***	**174**	**18**	**14**	**2**	**2**	**10.3**
**15_Tp**	***Trifolium pratense***	**147**	**15**	**11**	**2**	**2**	**10.2**
**16_Va**	***Vigna angularis***	**157**	**14**	**10**	**2**	**2**	**8.92**
**17_Vr**	***Vigna radiata***	**153**	**11**	**7**	**2**	**2**	**7.19**
**18_Vu**	***Vigna unguiculata***^a^	**61**	**4**	**4**	**0**	**0**	**6.56**
19_Cm	*Castanea mollissima*	98	3	1	1	1	3.06
20_Jr	*Juglans regia*	125	6	2	2	2	4.80
21_Jc	*Jatropha curcas*	113	4	2	1	1	3.54
22_Me	*Manihot esculenta*	184	5	2	2	1	2.72
23_Rc	*Ricinus communis*	121	3	1	1	1	2.48
24_Lu	*Linum usitatissimum*	195	7	2	2	3	3.59
25_Pe	*Populus euphratica*	178	4	1	2	1	2.25
26_Pt	*Populus trichocarpa*	201	4	1	2	1	1.99
27_Sp	*Salix purpurea*	219	4	1	3	0	1.83
28_Cs	*Cannabis sativa*	99	2	1	1	0	2.02
29_Hl	*Humulus lupulus*	103	5	1	2	2	4.85
30_Mn	*Morus notabilis*	116	3	1	1	1	2.59
31_Zj	*Ziziphus jujuba*	139	8	1	4	3	5.76
32_Fv	*Fragaria vesca*	112	3	1	1	1	2.68
33_Fa	*Fragaria x ananassa*	94	3	1	1	1	3.19
34_Md	*Malus domestica*	250	8	2	2	4	3.20
35_Pm	*Prunus mume*	118	3	1	1	1	2.54
36_Pp	*Prunus persica*	129	3	1	1	1	2.33
37_Pb	*Pyrus bretschneideri*	197	8	1	4	3	4.06
38_At	*Arabidopsis thaliana*	153	4	0	4	0	2.61
39_Cr	*Catharanthus roseus*	96	5	0	5	0	5.21
40_Cq	*Chenopodium quinoa*	200	8	0	8	0	4.00

Detailed classification of plants is summarised in Table S2. Fifteen Fabaceae plants are shown in bold. ^a^Genomes had not been sequenced. ^b^Draft genome database was used for sequence retrieval

### Three groups of subclade IVa bHLHs found in Fabaceae plants

**Fig. 1:**
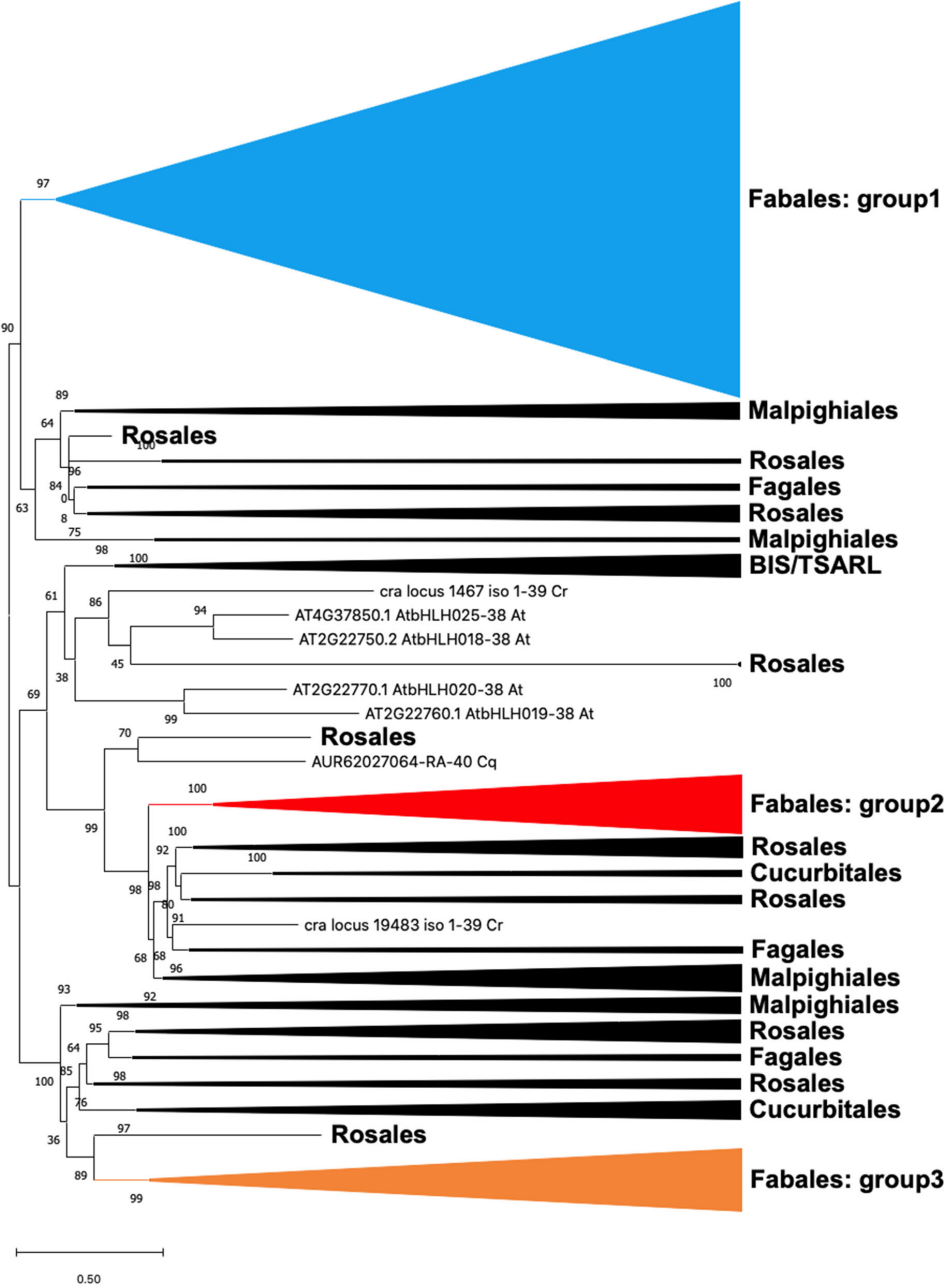
Phylogenetic tree of subclade IVa bHLH members in fabids. The approximately maximum-likelihood tree was built using FastTree and visualised with MEGA X. The local support values at each node were computed using 1000 resamples and the Shimodaira–Hasegawa test. Details are provided in Additional file 3: Fig. S2

To visualise the diversification of subclade IVa members in Fabaceae and other fabids, we constructed a phylogenetic tree using full-length sequences (Fig. 1, Additional file 3: Fig. S2). Subclade IVa bHLHs were further classified into three groups. Most Fabaceae subclade IVa bHLHs were included in group 1 (Table 1), which contained all MtTSARs and GubHLH3. Groups 2 and 3 had limited numbers of members, but were highly conserved among Fabaceae plants (Additional file 3: Fig. S2).

### Conservation of bHLH and ACT-like domains and exon-intron structures

**Fig. 2:**
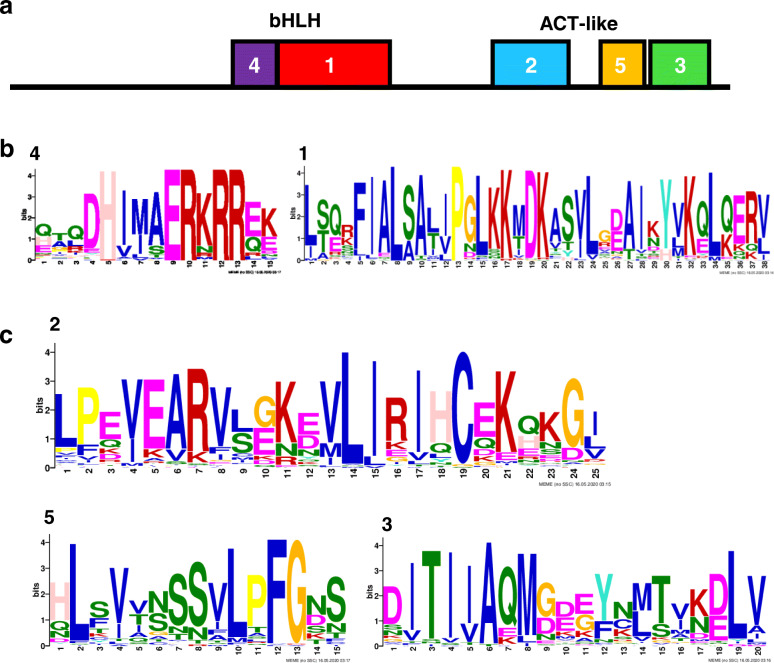
Conserved bHLH and ACT-like domains. Domain prediction and visualisation were performed using MEME. (a) Simplified domain structures of subclade IVa bHLHs. Conserved amino acid residues in (b) bHLH domains and (c) ACT-like domains. Details are provided in Additional file 3: Fig. S3

As described in previous studies [16, 28], bHLHs have highly conserved protein domains with other members of the same subclade. Subclade IVa bHLHs contain a bHLH domain and C-terminal ACT-like domain; the basic region contacts *cis*-motifs on genomic DNA, while the HLH and ACT-like domains are involved in dimerisation [18, 25, 32, 33]. Using MEME algorithm [34], we searched for these conserved domains (Fig. 2, Additional file 3: Fig. S3) in 82 subclade IVa bHLHs of *G. max*, *M. truncatula*, and *L. japonicus* (Additional file 1: Table S1). We found five motifs that were well conserved in almost all 82 proteins (Fig. 2a); two upstream motifs of the basic and HLH regions (Fig. 2b), and three motifs at the C-terminus corresponding to the ACT-like domain (Fig. 2c). Some group 1 members, GmbHLH105 and 106 and LjbHLH021, lacked the basic region (Additional file 3: Fig. S3) and these three proteins clustered together in the phylogenetic tree (Additional file 3: Fig. S2).

**Fig. 3:**
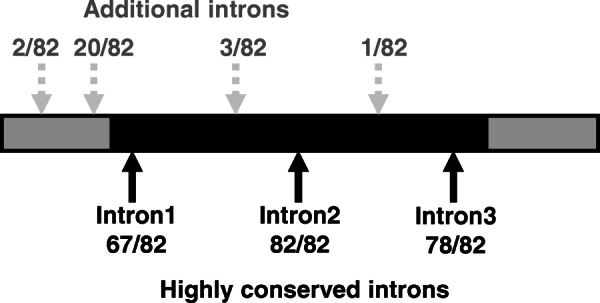
Simplified exon-intron organisation. Coding sequences (CDSs) and untranslated regions (UTRs) are indicated with black and grey boxes, respectively. Arrows indicate the positions of introns that are well conserved among 82 subclade IVa members from *G. max*, *M. truncatula*, and *L. japonicus*. Grey dashed arrows show the positions of introns found in some subclade IVa members. Details are provided in Additional file 1: Table S3

We confirmed that exon/intron structures are conserved among subclade IVa bHLH genes with some exceptions (Fig. 3). Most members had four exons and three introns. All 82 subclade IVa bHLH genes contained one intron within the HLH domain, but its length was highly variable (Additional file 1: Table S3). This conserved intron position corresponded to pattern D, as defined in a previous study [28]. *MtbHLH138*, *MtbHLH177*, *GmbHLH334*, and *LjbHLH014* lacked intron 3 and exon 4 (Additional file 1: Table S3), resulting in incomplete or absent ACT-like domains (Additional file 3: Fig. S3). As some members of groups 1, 2, and 3 gained or lacked introns (Additional file 1: Table S3), structural diversification may have occurred independently during their evolution.

Based on the highly conserved protein domains and exon-intron organisation across groups, we confirmed that groups 1, 2, and 3 were undoubtedly members of subclade IVa.

### Expression patterns of bHLH genes in each group

Using publicly available expression atlases of *G. max*, *M. truncatula*, and *L. japonicus*, we compared the expression patterns of homologous genes in each plant (Table 2). The orthologous genes in group 1 did not have a completely conserved expression profile across plant species. For instance, although *TSAR1* (*MtbHLH150*) was expressed more in leaves and petioles, the expression levels of its orthologous genes, *LjbHLH054* and *GmbHLH345*, were highest in nodules and flowers, respectively (Additional file 3: Fig. S4). Group 2 members were commonly expressed in nodules, except *GmbHLH116* and *GmbHLH334*, for which expression was not observed. Almost all genes in group 3 were expressed in underground tissues, namely roots and nodules. Three of the four genes in group 3 of *G. max* were also expressed in the pod shells.

**Table 2:** Tissues expressing subclade IVa bHLHs

	Name	Gene ID	Representative tissues expressing the gene
**Group1**	**TSAR3 homologues**		
	GmbHLH100	Glyma13g44570	Flower, pod shell (14–17 DAF)
	GmbHLH101	Glyma15g00750	Flower, pod shell (14–17 DAF)
	GmbHLH96	Glyma08g23060	Pod shell (7 DAF and 10–13 DAF)
	GmbHLH97	Glyma07g03050	Young leaf
	LjbHLH086	Lj3g3v0028570	Not available
		LotjaGi3g1v0005700	Immmature flower
	MtbHLH070	Medtr2g104590	Not available
	MtbHLH071/TSAR3	Medtr2g104650	24d seeds
	MtbHLH177	Medtr8g069740	Not available
	**TSAR2 homologues**		
	GmbHLH118	Glyma05g23530	Pod shell
	GmbHLH117	Glyma17g16720	Root hair, Root
	LjbHLH016	Lj0g3v0151119	Not available
		LotjaGi4g1v0240800	Nodules_10dpi, Root hair
	MtbHLH093/TSAR2	Medtr4g066460	Not available
		Mtr.9397.1.S1_at	Root, Bacterial and Fungal infections
	MtbHLH007	Medtr0246s0020	Not available
	MtbHLH092	Medtr4g066380	Not available
	MtbHLH094	Medtr4g067010	Not available
	MtbHLH004	Medtr0011s0210	Not available
	MtbHLH005	Medtr0011s0260	Not available
	MtbHLH009	Medtr0250s0040	Not available
	MtbHLH139	Medtr6g047570	Not available
	MtbHLH138	Medtr6g047550	Not available
	MtbHLH008	Medtr0246s0050	Not available
	MtbHLH113	Medtr4g098035	Not available
	**TSAR1 homologues**		
	GmbHLH345	Glyma18g48120	Young leaf, Flower, 1 cm Pod, Root
	LjbHLH054	Lj1g3v2883900	Nodule
	MtbHLH150/TSAR1	Medtr7g080780	Leaf, Root
**Group2**	GmbHLH114	Glyma13g32650	Nodule, Root, Seeds (10–13 DAF)
	GmbHLH115	Glyma15g06680	Nodule
	GmbHLH116	Glyma07g30420	Not expressed
	GmbHLH334	no correspondence	Not available
	LjbHLH032	Lj0g3v0292969	Root, Nodule
	LjbHLH152	Lj6g3v2171830	Nodule
	MtbHLH043	Medtr2g010450	Nodule (4d), Root
	MtbHLH107	Medtr4g092700	Nodule (4d, 14d, 10d)
**Group3**	GmbHLH110	Glyma17g16740	Pod shells
	GmbHLH111	Glyma05g23290	Root
	GmbHLH112	Glyma11g04690	Nodule, pod shell (14–17 DAF)
	GmbHLH113	Glyma01g40600	Nodule, pod shell (14–17 DAF)
	LjbHLH001^a^	Lj0g3v0034169	Not available
	LjbHLH014^a^	Lj0g3v0140069	Not available
	^a^	LotjaGi4g1v0185900	Root
	LjbHLH081	Lj2g3v1984450	Root, Nodule
	MtbHLH110	Medtr4g097920	Nodule (4d)
	MtbHLH111	Medtr4g097940	Nodule (4d)
	MtbHLH123	Medtr5g014640	Nodule (4d)

The expression of representative genes belonging to subclade IVa was determined using publicly available databases and summarised. ^a^LjbHLH001 and LjbHLH014 are found in the *L. japonicus* Miyakojima MG-20 accession, but both correspond to the same gene in the *L. japonicus* Gifu B-129 accession

## Discussion

One of the most diverse plant transcription factor families, bHLHs regulate many aspects of biological processes, including organ development, specialised metabolism, and the response to environmental stimuli [19]. Subclade IVa bHLH members appear to regulate specialised metabolism and defense responses [5, 19]. In this study, we showed that Fabaceae plants possessed a greater number of subclade IVa bHLH genes in their genomes than other fabids (Table 1, Fig. 1). *G. max* and *Glycine soja* had approximately double the number of total bHLHs and subclade IVa members compared to other Fabaceae, as they have experienced two whole-genome duplication events, doubling their genome size [35, 36]. Although the number of bHLHs in *M. truncatula* was similar to those of other Fabaceae plants, twice as many subclade IVa bHLHs were found in the *Medicago* genome (Table 1). Thus, *M. truncatula* likely duplicated its subclade IVa bHLHs during development of the hemolytic saponin biosynthesis pathway from the soyasaponin pathway (Additional file 3: Fig. S5).

Domain structures and exon-intron organisation were highly conserved among the 82 subclade IVa members derived from *G. max*, *M. truncatula*, and *L. japonicus* (Figs. 2, 3). Fabaceae subclade IVa bHLH proteins were clearly classified into three groups in the phylogenetic tree (Fig. 1). We found a strong bias in the number of Fabaceae bHLHs belonging to group 1, although no such bias was found in other fabids (Table 1). Group 1 may be a clade of transcription factors regulating saponin biosynthesis across a broad range of Fabaceae plants, as all MtTSARs and GubHLH3 were included in this group (Additional file 3: Fig. S2). Furthermore, the expression patterns of orthologous genes in group 1 were not conserved (Table 2), and the soyasaponin biosynthesis regulator, GubHLH3 was not the closest homologue of MtTSAR1 [22]. Thus, although the duplications of group 1 members apparently occurred in ancestral Fabaceae, their expression patterns and contributions to saponin biosynthesis may have differentiated after speciation. Therefore, we should search for candidate soyasaponin biosynthesis regulators among group 1 members.

Fewer members belonged to groups 2 and 3, but were highly conserved (Fig. 1, Table 1) and tended to be expressed in nodules and roots (Table 2). We confirmed the co-expression of *LjCYP93E1* (a soyasaponin biosynthetic gene) and *LjbHLH032* (group 2 subclade IVa bHLH) with a Pearson’s correlation coefficient of 0.797 (Additional file 3: Fig. S6). Furthermore, Fabaceae triterpene saponins likely play important roles in the rhizosphere, as reported in previous studies; increased saponin accumulation enhanced nodulation [16] and soyasaponins were the major component of root exudates [37]. These observations suggest that members of group 2 affect biological interactions in the rhizosphere through modulation of soyasaponin production. Generally, bHLH proteins form homo- and heterodimers that regulate the expression of target genes [18, 25, 32, 33]. The possibility that subclade IVa members in groups 2 and 3 also regulate saponin biosynthesis in Fabaceae is worthy of further investigation.

Fabaceae possessed more subclade IVa members, although there was no significant difference in the total numbers of bHLH genes between Fabaceae and non-Fabaceae (Mann–Whitney U test, *U* = 210, *p* = 0.1639). This suggested that other subclades in Fabaceae might have fewer genes. We roughly estimated how many genes were present in each subclade in selected species based on the phylogenetic relationships of the bHLH domains, and found no specific contraction in any subclade (Additional file 1: Table S4).

## Conclusions

In this study, we constructed a phylogenetic tree of full-length subclade IVa bHLH proteins from 40 plant species, mainly comprised of fabids. The results clearly indicated that subclade IVa bHLHs could be classified into three groups, and that Fabaceae plants contained a large number of group 1 members, including all saponin biosynthesis regulators identified to date. This information will help to uncover unidentified soyasaponin biosynthesis regulatory factors. On the other hand, no genes in groups 2 or 3 have yet been functionally characterised in Fabaceae. These genes are interesting targets for elucidating the evolution and functions of Fabaceae subclade IVa bHLH transcription factors.

## Methods

### Sequence retrieval

Representative protein sequences of *G. uralensis* were obtained from the *G. uralensis* genome database [38]. A total of 163 putative bHLH proteins were retrieved based on hidden Markov models (HMMs) of HLH domain (PF00010) downloaded from Pfam 32.0 [39, 40], using HMMER v3.3 software [41, 42]. The bHLH domain sequences and full-length sequences of bHLH proteins (only the primary isoforms) from other plant species were retrieved from PlantTFDB v5.0 [31, 43]. Subclade IVa members of selected species were identified using a BLAST search against all subclade IVa proteins of *A. thaliana* and *G. max* with an e-value threshold of <1e-50. The bHLH proteins selected are listed in Additional file 2.

### Phylogenetic tree analysis

Protein alignment of full-length bHLHs or bHLH domains was performed using Clustal Omega v1.2.3 [44] with the default settings. A Newick file was generated using FastTree v2.1.10 [45] with the default settings. The phylogenetic tree was visualised from the Newick file using MEGA X [46].

### Identification of conserved motifs and exon-intron structures

The conserved motifs of subclade IVa bHLHs from *G. max*, *L. japonicus*, and *M. truncatula* were predicted using MEME v5.1.1 [34, 47]. Exon-intron structures were retrieved from Phytozome v12.1 [48, 49] and the Legume Information System [50, 51].

### Expression pattern analysis

Expression patterns of bHLH genes were retrieved from *Lotus* Base [52, 53], Soybean eFP browser [54], Medicago eFP browser [55], and The *Medicago truncatula* Gene Expression Atlas [56, 57].

## Supplementary Information

**Additional file 1 Table S1.** Numbering of *G. max*, *M. truncatula*, and *L. japonicus* bHLH genes. **Table S2.** List of species used for phylogenetic tree analysis of subclade IVa bHLHs. **Table S3.** Exon-intron organisation. Genes with additional introns in their CDSs are indicated in red. The length of these additional introns is given in brackets. Introns within the HLH domain are highlighted in yellow. **Table S4.** Numbers of genes in each subclade.

**Additional file 2 Supplemental Data S1.** Protein sequences of 362 subclade IVa bHLHs used for phylogenetic tree analysis.

**Additional file 3 Fig. S1.** Phylogenetic tree of subclade IIIf and IVa bHLH proteins in *Glycine max* and *Arabidopsis thaliana*. **Fig. S2.** Detailed phylogenetic tree of subclade IVa bHLHs in fabids. **Fig. S3.** Predicted domains of subclade IVa bHLH proteins identified using MEME. **Fig. S4.** Expression patterns of TSAR1 orthologues. Data were retrieved from *Lotus* Base, Soybean eFP browser, and Medicago eFP browser. **Fig. S5.** Biosynthesis pathways for aglycones of soyasaponins and hemolytic saponins from *M. truncatula*. This figure shows representative aglycones of soyasaponins and hemolytic saponins. Cytochrome P450 monooxygenases have been found to oxidise different carbon positions of the β-amyrin backbone [cytochrome P450 enzymes (positions to be oxidised), *characterised in soybean]. Although the soyasaponin pathway is common among Fabaceae, only *Medicago* spp. acquired the hemolytic pathway. **Fig. S6.** Expression of *LjCYP93E1* and *LjbHLH032*. Data retrieved from *Lotus* Base. *Lj1g3v3555800*: *LjCYP93E1*; *Lj0g3v0292969*: *LjbHLH032.*

## Data Availability

The all data analyzed in this study are available in the publications and the websites cited in Methods section.

## Abbreviations

*A. thaliana*: *Arabidopsis thaliana*
bHLH: basic-helix-loop-helix
BLAST: Basic Local Alignment Search Tool
BIS: bHLH iridoid synthesis
*C. roseus*: *Catharanthus roseus*
*C. quinoa*: *Chenopodium quinoa*
CDS: Coding sequence
*G. max*: *Glycine max*
*G. soja*: *Glycine soja*
*G. uralensis*: *Glycyrrhiza uralensis*
HMM: Hidden Markov model
*L. japonicus*: *Lotus japonicus*
*M. truncatula*: *Medicago truncatula*
MeJA: Methyl jasmonate
MIA: Monoterpenoid indole alkaloid
TSAR: TRITERPENE SAPONIN BIOSYTHESIS ACTIVATING REGULATOR
TSARL: TSAR-like
UTR: Untranslated region
