# Supplementary material for: Insights into the diversification of subclade IVa bHLH transcription factors in Fabaceae
Source: BMC Plant Biol. 2021 Feb 23;21:109. doi: 10.1186/s12870-021-02887-w (PMC7901066; doi:10.1186/s12870-021-02887-w)
Supplement: Supplementary file 2 — Additional file 2 Supplemental Data S1. Protein sequences of 362 subclade IVa bHLHs used for phylogenetic tree analysis. [file 12870_2021_2887_MOESM2_ESM.doc]

**Supplemental Data S1.** Protein sequences of 362 subclade IVa bHLHs used for phylogenetic tree analysis.

>Cla000121-01_Cl

MEDNGFVNQWHKSSMDELGMLPLAATFGENLQHSYAQTNIDQKASLNHSHNAILGYVKQLKSDGWSSYQTTNHLPNSQVIAYPTASVSASNYTNQMDFTRPKEEVVCPQSISNLPSDMLVSQGSLAYQSHVTKSNRGTRSPSTNSRIPQAQDHILAERRRREKLSQRFIALSAIVPGLKKMDKASVLGDAIKYLKQLQEKVKILEEQTRRKDIESVVFVKKSQVFADGNNTSKEEDEPLPEIEARFCDKNVLIRIHCEKKKDVLEKTITEIEKLHLTIVNSSVMSFGSLALDITIIAQMDNEFCLTLKDLVKKLQSTLRSF

>Cla014072-01_Cl

MEISSSTRWISEWEIEDAPPFMDEFKIDPFDFSFDEFKFEEYSGGTDFYGWPVPPSAVVEDGGIKCQIYGSNYKTDWSSSSSHTISFDSSNCKSGNGSVNLGMAGYESVNIISESYWNNNEKSWKRSCCSMNRNNGLHAREHVIAERRRRQKLSQRFIALSALIPDLNKVDKASILGGAIRHVKELQERLKVVEEQATSKMSEAKSAVYVKRTNLQVSSTDDDTSSSDYDSFSGRPTPEIEARFSNKDVLIRIHCQNRKGCLSYLLDKIDNLNNLTILNSCALPFGQSNLDITIVAQVDVGFCMTGEDVVKNLRQALLDFN

>Cla014073-01_Cl

MDDLEMNPFDCTLEDLSNFQAFSDESYTSHVDLENSVQTPAAPPAKQARTTGSSRKIASMAASSSSSHIISFGNSHSSCNPKLEMGCELSGNIVDLSSVVAQPCNDNNNNNNTPNYYYSPRNHGVGIKRSAAAINNNRSPLLAQDHVIAERKRREKLSQRFVALSALIPDLKKMDKASILADAITYIKDLQERLKVANEQAAKGTMESVVFVNRSDDVSTIVVGDDSSEENSSSSDGAIPDIEARVSGKDVLLRIHGKKCKGCLSNILNQIEKLNLTVLNSSALPFGNFRLDITIIAQMDDDFSMTVKELVQKLRQASLEFV

>Cla014074-01_Cl

MMECSYEYETNNYTLDELNFPGSKKVNTLTSSQLISFGDYSAEIENKFDYGSCVNWVGVDKDKDFSFLISNQNNGNLIGFEEEGHGIKKAATTIMGPNNYGNGKEHVIAERRRREKLSQNFVALSALIPGLNKRDKASVLGGAIKYVKELQERLKWAEEEAADQKRVIKSVVFGPKRIDLDSDSDDKTSSWDENGGVFSVQPIPKIETRVLEKDVLVRIHCKKHKGCLTNILSQIEKLNLTIVNSCVLPFGHSRLDITIVAKMEVGFCMTARNLGKKLRETLVEFI

>MELO3C007678P1-02_Cm

MDEFKIDPFEFSLDALQFDDLYELPVPPYAVEEKPAKQVKVEKEEEEQAGAGGINKCKTTFVGNNYKGRDSSLSSSSRIISFENNWNHESVNNWNSNNGKRSCSMNRNAMNGREHVIAERKRREKLSQRFIALSALIPGLNKVDKASILGGAIRHVKELQERLKGVEEQTTSKTSEPQVPVVCVKRTTLQPSSSDDDTSSSDENSFSGRLRSTPEIEARFLNNDVLIRIHCHKRKGCLSYLLNRIQSLNNLTILNTCALPFSHSNLDITIVAQMDVGFHMTVEDVVKNLRQALLDFN

>MELO3C007679P1-02_Cm

MEISSANWLSEMELESSFMNDLEMNPFECTLEELSSFQTFSDESYTSHVDLDNNSVQTPTAPPPAKQARTSGSSRRIASMATSSSSSQIISFGNVELSSMVAQPSYDNKNNNKTPNYYCSPNKNHGVGIKRSVAAMNSNNRSPLVAQEHVLAERKRREKLSQRFVALSALIPDLKKMDKASILGDAITYIKDLQERLKVANEQAAKATVESVVFVNKSEDASTIVVSDDSSEENSSSSSDGAIPDVEARVSGKDVLLKIHCKKCTGCLSNILNQIEKLNLTVLNSSALPFGNFRVDITIIAQMDDDFSITVKELVQKLRQASLKFM

>MELO3C007680P1-02_Cm

MMECSYEYERMKKCPHDYTLDELNFPAKEKVNNLRSSSQLVSFGDYSGEIENYKFDNYGNLIGSGEKGHEIKKDYCSTIIMGQLPNRNRNHNNSEHVIAERRRREKIRQNFIALSALIPGLIKRDKASVLGGAIEYVKELQERLKWAEEEAEKQKSVIKSVVFVKRINLDSDDSENETFSLDENGDRSSVRSVPTIEIRVLEKDVMVRIHCKKRQGCYTSILSQIEKLKLTILNSCVSPFGESRLDITIVAEMEAGFCMTPMDVGKNLRETLVEFI

>MELO3C018555P1-02_Cm

MEDNGFVNQWHKSSMDDLGLHPLAAAFGENLQHSYAQTNIDQKASLNHSHNAILGYVKQLKSDGWSSYQTTNHLPNSQVVAYPTVSVSASNYTNQMDFTRPKEEVVCPQSISNLPSDMLISQGSLAHQSHTTKSNRGTRSPSTNSRIPQAQDHILAERRRREKLSQRFIALSAIIPGLKKMDKASVLGDAIKYLKQLQEKVKILEEQTRRKDIESVVFVKKSHVFADGNDTSKEEDEPLPEIEARFCDKNVLIRIHCEKKKDVIERTVAEIEKLHLIIVNSSVMSFGSLAFDITIIAQVITTHHLHNPPNT

>Cucsa.103610.1-03_Cs

DALQFDDLYDLPVLPYAVEERPAKRVKVEEDVEAGGGGINKCKTTFVGNNHKGDSSLSSSQIISFENNWNYESVKNWNCTNGKRSCSMNGREHVIAERKRREKLSQRFIALSALIPDLNKADKASILGGAIRHVKELQERLKVVEEQTTSKTSKPQSPVVCVKRTTLQPSSSDDDTSSSDENSFSGRLRSTPEIEVRFVNNDVLIRIHCHKRKGCLSYLLNKIQSFNNLTILNTSALPFSHSNLDITIVAQMDVGFHMTVEDVVKNLRQALLDFN

>Cucsa.103620.1-03_Cs

MNDLEMNPFECTLEELSSFQTFSDESYTSHVDLDNSSVQTPAAPPPAKQARTSSGSSRRISSMATSSSSSQIISFGNIEMSPMVAQPSYDNNNNNNKTSNYYCSPNKNHGVGIKRSAAAAMNSNNRSPLVAQDHVLAERKRREKLSQRFVALSALIPDLKKMDKASILGDAITYIKDLQERLKVANEQAAKATVESVVFVNKSDDASTIIASDDSSEENSSSSSDGAIPDVEARVSGKDVLLRIHGKKCKGCLSNILNQIEKLNLTVLNSSALPFGNFRLDITIIAQMDDDFSMTVKELVQKLRQASLEFM

>Cucsa.103630.1-03_Cs

MMECSYEYERMKKYPHDYTLDELNFPAMEKVNNSRSSGQLISFGDYSGEIENYKFDDYGNLIGFEEEEHEIKKDYCSTIIMGQLPNHNHNNNNSEHVIAERRRREKIRQNFIALSALIPGLIKRDKASVLGGAIKFVKELQERLKWAEEKEKEQKRVIKSVVFVKTINLDSDFDNETFSLDENGGRFSVRSVPTIETRVLEKDVLVRIHCKKHKGCYTSIVSEIEKLKLTIVNSCVFPFGQSRLDITIIAEMEAGFCMTPMDLGKKLRETLIEFI

>Cucsa.218800.1-03_Cs

MEKSSMVVFSEMGMEDNGFVNQWHKSSMDDLGLHPLAAAFGENLQHSYDQSNIDQKASLNHSHNAILGYMKQLKSDGWSSYQTTNHLPNSQVVAYPTVSVSASNYMNQMDFSRPKEEVVCPQSISNLPSDMLISQDSLAHQSHTTKSNRGTRSPSRNSRIPQAQDHILAERRRREKLSQRFIALSAIVPGLKKMDKASVLGDAIKYLKQLQEKVKILEEQTRRKDIESVVFVKKSHVFPDGNDTSKEEDEPLPEIEARICDKNVLIRIHCEKKKDIIEKTIAEIENLHLTIVNSSVMSFGSLALDITIIAQMDNEFCLTLKDLVKNLQSTLRSF

>Aradu.394BE-04_Ad

MLGSINDETFSPKLSSSSSILSLRSQILSFDNSDSLSSSPSNNTTEFYELKNTLNQRQDSVETPNNNNKKSDSLNTQNVEAKSTSTLGKRSPSHAHDHIIAERKRREKISQSFIALAALVPGLKKMDKASVLGDSIRYVKELKERLAVLEEESKKTKAKKAEQLHGHGAAVFSSLCEETIDGSLPQVEAREWGQQVLLRIHCWKEEGILVRILSEIQSLQLMLLNSSVLSFGDSILDITIIVQAGEGYNLTLNELVKNLRTATLKLMS

>Aradu.5Q1VY-04_Ad

MEELNGLATNWLSDLDIDDYELFSECNLKKFLDEDEENQFDEMVMSAVVGEEEEEEEERPTKQLRTCSSSSITNYVSSNSSSSSSPTSQILSFENSYNPSSQFYGFEFDATPNDKVSPQPQVGNNNDKNKLEQTRKPQSQGSKRQAAHSHDHIMAERKRREKLSQSLIALAALIPGLKKMDKASVLGDAIKYVKELQGRLKVLEEENKREVESVVIVKNPRFITSSSDDDSSSCDDDTLEADGEAALAHVEARVAAAEKEVLLRIHCKKQKGIYVKLLSEIQSLHLYVVHSSVLPFGDSVLDITIVAQMGTEYKLSIRDLVRNLRVATLKTMSSS

>Aradu.83N8C-04_Ad

MMQISSTNKYLPEFGMDESVFFHNQYPMMDSSTITSSPSPWPQILDDIDFNQIQESFSSASSPKSYTSNKRFNSSSAAGTTMPQSSFSPIERPTKQQKNAYSHGISTNTTTTTSNEFMHSNASSSVASYNHRHQQLYNNNPSSDQGNHVLRPKTESVCSENLDFATVVSHADYLSGYDKANKGSAAAATTRNPTQAQDHVIAERKRREKLSQRFIALSAIVPGLKKMDKASVLGDAIKYLKQLQEKVKILEEQVAEKTVESAIFVKRSILFAEDNGSSSEENPDQKIPEIEARISGKDVLIRIHCDKHSGIVPKIINEIEKHDLSVQSSSFLPFGNNSLDITIVAQRI

>Aradu.C74HT-04_Ad

MESSSPSWLHDLEMEDDYNNFFPKEYRMNNSIDDYDEDLFFAHDMGNSIMFESPSNNNNYSSVDETSFDDERPSKFLKTSTNTDNNSYYSSLSPNFSSLSSSSITSFQPQILCFDNSNSSLNNNDNTTQLYDLDYTTLNTETKLKEVVNGSSKNQNLVTKSSSKGSKRSPSNAQDHIIAERKRREKISQSFIALAALLITNIRILTIQIDKVTVLENAIKYVKDLKIRLTTLEQENNEKIKEIVEEPSVIVLNKRLPHDNESDDESVIVDNTSNHDSSSLLHHVEARVSGQHVLIRIQCQKHKGILVKLLSEIQGHNLLVLNSSALPFGDSVLDITIITRV

>Aradu.DP2D5-04_Ad

MISTEESWTTWLCDLEEEDYSFINGIIAAPNNYSNSQMSNNDNERPSKLLKSTTPTPRRRRTTGSAGTGRSPQHAHDHIIAERMRREKISQQFIALSALIPGLKKMDKATVLGDAIKYVKQLQEQVKVLETESKRKSAESVVYVEKSEVCGEEDVSVSDTWSNSGGDGNSSYEVSKAVSRSVLPEVEARVSEKNVLIRIHCEKHKGVLMHILKLIDKLHLSVLNTTSLPFGASIVDITITAEMDDKFSLSAKELARNIRVGVLQSM

>Aradu.GY22L-04_Ad

MEQCWENWPLHTEIEHHDHGDFFLSLEQCQKPPDDDDDFLREILQMPPDFIDNHSTVDLVTTGTAESAEGKRPRTFILSFDNSTIIPAMAEQQQPLGVGAGAATTTPLPSLSKKRNLLQKPQQARIPVPVPVPQPQPGAAKRSRNDSQIVDHIMAERKRRQQLTQMFIALSATIPGLKKTDKASILGEAINYVKQLQERVRELEKRNNDNKRGPTEPVIFLNKTQLLCRNNEDSTSEEEEEEEEEEEVEDWRSKEEKQVLPDVEARMLEKEKEVLIEIHCEKENGIEVKILEQLENLHLSVTGSSVLPFGNSTLGITIIAKMGDAYTMTLHDLLTNLRQLLLINNTTDPY

>Aradu.HK2E0-04_Ad

MDISSLRGLTDLEIMEDPTYLHQWHLSSIYEPTLLPIAAAFGETLQQHSFSHPSFNSKTSMETSLSNIDRPTKHLKNNSWCPTKTPSEAQFASCSTLLSFVDPNYINQLEVVKSKDEMVCPKMNDTTPKDMNFQGTLGNQYVFEASQATKHVGPRSRLSQPQDHIIAERKRREKLSQRFIALSALVPGLQKTDKASVLGDAIKYLKQLQEKVKALEEEQNKRKAVESVVFVKKSQLSNDAEDASLEYEGIFHEALPEIEARFCDKNVLVRIHCEKNRGVVEKSINQIEKLHLKVTNSSAMTFGSWALDITIIAQMEKGFCMTVKELVRNLRSAFASII

>Aradu.LYC6U-04_Ad

MSQNSEVRSHQKRLMEEVEIHVSNGGVVQEVGERLSLPKLMMMMMMSKSNSSKSIVNSGNTAKKARRSSSECLDHIVAERKRRQEITQRFIALSATIPHLKKIDKVSILSEAIAYIKQLKEQGKKLEEESRKKNRTVVESVSLVNKRHDAACELVEARALEKQVLIRIHCHGHKAVPQVFSHLTNLDLSIVSTSVLPFGTCAIDITIVAQMGEKYRASMKDLVQSLRLAIPLC

>Aradu.M594W-04_Ad

MGEPCHNYWYSDMGIQDDDIFNQRYKINSSLIVDEDHIIREIMDHHHHQPAFSSESDNSHSPTNNNQIKGGGSGNTSSFVFNNNNQHAPLLLDMKASTSTSSPRSYILSFHDSTVIATTAAAAATPPPPLPSLETYNNNEKRPYQHIEVALENQAKKVRSSSETLDHIMTERKRRRELTERFIALSATIPGLKKIDKSTILSEAISHVKQLKQRVKELEEQKKKISVESVSFIIRKSHLMNGTNNKDDEKGAINNKAATSEALLPTVEARVLKNDVLIRIHCMKQSGIMLKILRHLKSFDLSAISNSVLPFGNSTLDITIIAQIGDKFNVTMNDLVKNLRLSILESPNDDEEPHNSN

>Aradu.Q2I1J-04_Ad

MEEEEEENDDTIIMNNYSLNEEQEFLMKIFNGRPDFSSPESSEHYYYSSPNNNNNNSYNNNDPNNCDTSPNNNNSSVSFEDTRVQKSKSSNSSSSTYLLSFEPNSFKGRSRGDDGFELFEASMNKNDEGGTTKKRKGKTVDHIIAERKRRQDLTRSIIQLSATIPGLKKMDKAHVIRESLSYIKILQDRVKELENQIKDRRVDSAIFIGRSQDSLSTDKSTISCEITSDNNNGGGGFNESSLEIEAKVMEKEVLIRIQCEKQKNNIIMLKIHAFLDKLHLSIASNSVIPFGTSTLVIITIVAEMDNGGKFSMTMDELVKSLREDLMETNNNAW

>Aradu.S0KU9-04_Ad

MMEIASSNYLAEFGIEEYSSSFQEYPMMMNSFEEMLDKFEMDMQSMSSAYSETKPPPHQLQSPFNTAMPSRSASPSPPKLISFEAPSLPNSSNIKNPNLMMDDHIHFSAFFNHDNPPHKVLPATARNPIQAQEHVIAERKRREKLSQRFVALSAMVPGLKKMDKASILGDAIKYVKQLQERVQFLEEEKARKKTMVESGVAVKRCFVFVEDEDNNENEISAAAALLDGNCNTLPEIKARVSGKDVLIRIHCHKQECKNSRGAREAAILSVLEKHNLTVHTTTSLPFGNDTLDITILAQMKKECSIRTKDLVGSLRVALTQFS

>Aradu.WC9V5-04_Ad

MLVGEDQHKKSTKQQRGGGAMKVLICHERILGRVDYLMEISSELGIMEDPNSFLWHLSSIDTCATTLAVFGDSLQKNNPLFCNSNLMNSKISMMETTTSPTTTIIERPAKQLRSNNTSNWSSHINKTPESHFVGSCSNNLLSFVDNTNHHHHQLGLVMKPKVEIMSSSSPNNIDTQGTTLLGNNNNHHHNHENYLFKESSCHEAKNFGQRPKLSSHQPHDHIIAERKRREKLSQRFIALSALVPGLKKMDKASVLGDAIKYLKQMQEKVSALEEEQKKKKTVESVVMVKKSQLCNDDEDSCSSETEPLPEIEARFCERNVLIRVHCEKKKGVIENTIIQIEKLHLKVINSSVLTFGNFALDITIIAQMDMEFSMTVKELVKNLRSAFSTFM

>Aradu.X1TYZ-04_Ad

MLQPSFSSENSESDNLQNTVGSSSSPVIEEVPQDLTAENSNNKNKKNNTMKQPRRSSSSSSSLRCSSPRRYILSFDNSTMTPATPNQLQEDPYYANNNNNNNNNNNNHKNKKNKDPSSCDYSSPKRVVPICTNNSENKKEEGAITTKRARSSSQTMDHIMAERKRRQELTERFIALSATIPGLTKTDKASILRAAIDYVKQLQEQVQELEKKNKKRSRESVILVKKSKSNNEKILLEESTIISSETRRSEDGGTGLPDIEARFMGKDVLIEIHCEKENGIEMKILNQLENLHLFVTGSSVLPFGNSALGITIIAKMGDSCEMTVNDVVRNLRQVFLKAHQNSHGI

>Ahy009091-05_Ah

XCNLKKFLDEDEENQFDEMVMSAVVGEEEEEEEERPTKQLRTCSSSSITNYVSSNSSSSSSPTSQILSFENSYNPSSQFYGFEFDATPNDKVSPQPQVGNNNDKNKLEQTRKPQSQGSKRQAAHSHDHIMAERKRREKLSQSLIALAALIPGLKKMDKASVLGDAIKYVKELQGRLKVLEEENKREVESVVIVKNPRFITSSSDDDSSSCDDDTLEADGEAALAHVEARVAAAEKEVLLRIHCKKQKGIYVKLLSEIQSLHLYVVHSSVLPFGDSVLDITIVAQMGTEYKLSIRDLVRNLRXGYAENDVVVIAGT

>Ahy009616-05_Ah

MDDFSWEHLHLQLEMDDENMNNNNGVDDDNNEDDDEFLREILMLQPSFSSENSESDNLQNTVGSSSSPVIEEVPQDLTAENSNNKNKKNNTMKQPRRSSSSSSLRCSSPRRYILSFDNSTMTPATPNQLQEDPYYANNNNNNHKNKKNKDPSSCDYSSPKRVVPICTNNSENKKEEGAITTKRARSSSQTMDHIMAERKRRQELTERFIALSATIPGLTKTDKASILRAAIDYVKQLQEQVQELEKKNKKRSRESVILVKKSKS

>Ahy016096-05_Ah

MEQCWENWPLHTEIEHHDHGDFFLSLEQCQKPPDDDDDFLREILQMPPDFIDNHSTVDLVTTGTTESAEGKRPRTFILSFDNSTIIPAMAEQQQPLGVGAGAATTTPLPSLSKKRNLPQKPQQARIPVPVPHPQPGAAKRSRNDSQIVDHIMAERKRRQQLTQMFIALSATIPGLKKTDKASILGEAINYVKQLQERVRELEKRNNDNKRGPTEPVIFLNKTQLLCRNNEDSTSEEEEEEDEEEEEVEDWRSKEEKQVLPDVEARMLEKEKEVLIEIHCEKENGIEVKILEQLENLHLSVTGSSVLPFGNSTLGITIIAKMGDAYTMTLHDLLTNLRQLLLINNTTDPY

>Ahy020163-05_Ah

MISTEESWTTWLCDLEEEDYSFINGIIAAPNNYSNSQSQMSNNDNERPSKLPKSTTPTPRRRRTGSAGTGRSPQHAHDHIIAERMRREKISQQFIALSALIPGLKKMDKATVLGDAIKYVKQLQEQVKVLETESKRKSAESVVYVEKSEVCGEEDVSVSDTWSNSGGDGNSSYEVSKAVSRSVLPEVEARVSEKNVLIRIHCEKHKGVLMHILKLIDKLHLSVLNTTSLPFGASIVDITITAEMDDKFSLSAKELARNIRVGVLQSM

>Araip.007DK-06_Ai

DNEAGNNGGGVVQEVGERLSLPKLMMMMMMSKSNSSKSIVNGGNIAKKARRSSSECLDHIMAERKRRQEITQKFIALSATIPHLKKIDKASILSEAIAYIKQLKEQGKKLEEESRKKNRTVIESVSLVNKRHGAYELVEARALEKQVLIRIHCHGHKGVPQVFNHLTNLDLSVVSTSVLPFGTCALDITIVAQMGEKYSASMKDLVQSLRLAIPLC

>Araip.13D8C-06_Ai

MEELNASATNWLSDLDIDDYELFSECNLKKFLDEDEENQFDEMVMSAVLGEEEEEEEERPTKQLRTCSSSSITNYVSSNSSSSSSPTSQILSFENSYNPSSQFYGFEFDATPNDKVSPQPQVGNNNDKNKVEQTRKPQSQGSKRHAAHSHDHIMAERKRREKLSQSLIALAALIPGLKKMDKASVLGDAIKYVKELQGRLKVLEEENKREVESVVIVKNPRFITSSSDDDSSSCDDDTLEADGEAAVAHVEARVAAAEKEVLLRIHCKKQKGIYVKLLSEIQSLHLYVVHSSVLPFGDSVLDITIVAQMGTEYKLSIRDLVRNLRVATLKTMSSS

>Araip.7P91S-06_Ai

MGEPCHNYWYSDMGIQDDDIFNQRYKINTSLIVDKDHIIREIMDHHDYHHQPAFSSESDNSHSPTNNNRSGGSGNTRGFVFNNNNNNGSMSMFSQQHQHAPLLLDMKASTSTSSPRSYILSFHDSTVIATTAAAAATPPPPPSLETYNNNGKRPYQHIEVPLENQAKKVRSSSETLDHIMTERKRRRELTERFIALSATIPGLKKIDKSTILSEAISHVKQLKQRVKELEEQRKKISVESVSFIIRKSHLINGTTNNKVDDEGAINNKAATSEIALLPTVEARVFKNDVLIRIHCMKQSGIMLKILGHLKSFDLSAISNSVLPFGNSTLDITIIAQVYIYIYAQY

>Araip.865PM-06_Ai

MAERKRRQQLTQMFIALSATIPGLKKTDKASILGEAINYVKQLQERVRELEKRNNDNKRGPTEPVIFLNKTQLLCRNNEDSTSEEEEEEEEEEVEDWRSKEEKQVLPDVEARMLEKEKEVLIEIHCEKENGIEVKILEQLENLHLSVTGSSVLPFGNSTLGITIIAKMGDAYTMTLHDLLTNLRQLLLNIDQQHY

>Araip.I1L37-06_Ai

MDHIMAERKRRQELTERFIALSATIPGLTKTDKASILRAAIDYVKQLQERVQELEKQNKKRSRESVILVKKSKNINNEKILLEESTIISTSETRRSEDGGTGLPDIEARFMGKDVLIEIHCEKENGIEMKILNQLENLHLFVTGSSVLPFGNSALGITIIAKMGDSCEMTVNDVVRNLREVFLKAHQNSHGI

>Araip.J3ZJD-06_Ai

MDISSLRGLTDLEIMEDPTYLHQWHLSSIDEPTLLPIAAAFGETLQQHSFSHPSFNPKTSMETSLSNIDRPTKHLKNNSWCPTKTPSEAQFASCSTLLSFVDPNYINQLEVVKSKDEMVCPKMNDTTPKDMNFQGTLGNQYVFEASQATKHVGPRSRLSQPQDHIIAERKRREKLSQRFIALSALVPGLQKTDKASVLGDAIKYLKQLQEKVKALEEEQNKRKAVESVVFVKKSQLSNDAEDASLEYEGIFHEALPEIEARFCDKNVLIRIHCEKNRGVVEKSINQIEKLHLKVTNSSAMTFGSWALDITIIAQMEKGFCMTVKELVRNLRSAFASII

>Araip.JIB5P-06_Ai

MKISMLVGEDQHKKSTKQQRGGGAMKVLICQHERILGRVNYLMEISSELGIMEDPNSFLWHLSSIDTCATTLAVFGDSLQKNNPLFCNSNLMNSKISMMETTTSPTATIIERPAKQLRSNNTSNWSSHINKTPESHFVGSCSNNILSFVDNTNHHHHQLGLVMKPKVEIMNSSSPNNIDTQGTTLLGNNNNNHHNHENYLFKESSCHEAKNFGQRPKLSSHQPHDHIIAERKRREKLSQRFIALSALVPGLKKMDKASVLGDAIKYLKQMQEKVSALEEEQKKKKTVESVVMVKKSQLCNDDEDSCSSETEPLPEIEARFCERNVLVRVHCEKKKGVIENTIIEIEKLHLKVINSSVLTFGTFALDITIIAQMDMEFSMTVKELVKNLRLAFSTFM

>Araip.K0K3F-06_Ai

MEKSNTPMDTSAASWLSELEIDEDYNFFPDLDFDLVDEEDFLSHELIASEDLQGKSALQDQSLSAECNSKELSNCCTDEMMSFEEMLRNINDETFSPKLSSSSSQILSFDNSDSLSSSPPNNTTQFYELKNSLNQRQDSVETPNNNNNKKSDSLNTQNVEAKSTSTLGKRSPSHAHDHIIAERKRREKISQSFIALAALVPGLKKMDKASVLGDSIKYVKELKERLAVLEEESKKTKALPTVVLNKAEQLHAHGAAVFSSLCEEETIDGLPQVEAREWGQQVLLRIHCWKEEGILVRILSEIQSLQLMVLNSSVLSFGDSILDITIIVQAGEGYNLTLNELVKNLRMATLKLMS

>Araip.LC4KN-06_Ai

MISTEESWTTWLCDLEEEDYSFINGIIADPNNYSNSQMSNNDNERPSKLLKSTTPTPTRRRRTGSAGTGRSPQHAHDHIIAERMRREKISQQFIALSALIPGLKKMDKATVLGDAIKYVKQLQEQVKVLETESKRKSAESVVYVEKSEVCGEEDVSDTWSNSGGDGNSSYEVSKAVSRSVLPEVEARVSEKNVLIRIHCEKHKGVLMHILKLIDKLHLSVLNTTSLPFGTSIVDITVTAEMDDKFSLSAKELARNIRVGLLQSM

>Araip.PVV4Q-06_Ai

MMEIASSNYLAEFGMEEYSSSFQEYPMMMNSFEEMLDKFEMDMQSMSSASPECYSNSETKPPPHQLQTPLNTITTTTTTAAMPSRSASPSPPKLISFEAPSLPNSSNIKNPNLMMDDHIPFSAFFNYDNPPHKVLPATARNPVQAQEHVIAERKRREKLSQRFVALSAMVPGLKKMDKASILGDAIKYVKQLQERVQFLEEEKARKKSMVESGVAVKRCFVFVDDEDNNENEISAAAALLDGNCNTLPEIKARVSGKDVLIRIHCHKQECKNSSRGAREAAILSVLEKHNLTVHTTTSLPFGNDTLDITILAQMNKEYSIRTKDLVGSLRVALTQFS

>Araip.SB6JF-06_Ai

MDESVFFHNQYPMMDSSTITSSPTPWPQINLDDIDFNQIQESFSSASSPKSYTSNKRFNSSFSPIERPTKQQKNAYSHGISTNTTTTTSNEFMVPKASSSSSSQIISFEQHSNASSVASYNHHHQQLYNNNPSSDQGNHVLKPKTESVCSENLDFASVVSQADKANKGSAGAATTRNPTQAQDHVIAERKRREKLSQRFIALSAIVPGLKKMDKASVLGDAIKYLKQLQEKVKILEEQVAEKTVESAVFVKRSILFAEDNGSSSEENPEIEARISGKDVLIRIHCDKHSGIVPKIINEIEKHDLSVQSSSFLPFGNNSLDITIVAQVNINFNFSLINLL

>C.cajan_00681-07_Cc

METALVDFERPTKHHQNMSWNPSKNAQTSETEFVSFPNPFSFVGSNVISPLGLVKAMDEMDCPITISSTTPLESTSQGTLSQKIGTRSKLTHPQDHIIAERKRREKLSQQFIALSSLVPGLQKTDKASVLADAIKYLKQLQEKVRALEEEQNMKKNVESVVIVKKSQLSNDVNNNSLEYHGQFDEALPEIEARFCERNVLIRVHCGKSKGVVEKTIQEIEKLHLTVTNSSAMIFGRCSLDLTIIAQMDMEFCMGVKDLVRNLHSAFTSFM

>C.cajan_02946-07_Cc

MDDHEVNECLCHANPFDEEFLREILPQPQEALVTTPTSSQYAEDHKPITSSATTYILSFEKSAALLPADPDSCSAWKENSQSQLLKEILPLPSSRVNQGTKKTRSASESLDHLMSERNRRQELTRKFIALAATIPGLKKMDKAHVLREAINYVKQLQERVDELEEDIQKNDVESAITITKSHLCVDDKCNDLALVEVEARVLGKEVLIKIHCAKQKGVLLKIMSQLERLHLYISTSNVLPFGNTLDITIIAQMGEKYKLVMKDLVKELRQVAMNEAM

>C.cajan_12990-07_Cc

MSNSSQDDKNKIFGESPANTLKTGTSNSANTEYLSQKNDSSLSYILSFDNVVNPAPVLNIDSTTMKPKDGKVARNQNKEPKNNNSVSLSRSPLHARDHIIAERKRREKISQQFIALSALIPGLKKMDKASVLGDAIKHVKELQEQVKLLEEQNKRKRVESMVYVEKSKLSWDEDVSDTSSNSGDGNSYGPSKTNASSLPEVEARVSEKHVLIRIHCDKQRGLFMNILKEVENLHLSVMNSSILLFGTSKLDITIVAEMEQEFGLSVKELARNLRVGLMQFM

>C.cajan_12992-07_Cc

LEYPTFFDQYPMDSFACPPLDDFDFESFSGSPESNSSYQFNSESTPNCFPVESLDQSYSPARPITKRLKKFSTLNTTCAGDLTPHKVSASPYSQLISFGHFNAPSGAPKQFDNYVKPKIENTYSENMDFSAFVSQGSYEDKSFLSSDNLTNQPVITTRNPIQAQEHVIAERKRREKLSQRFIALSAILPGLKKMDKASVLGDAIKYVKQLQERVQTLEEQAAKRTVGSSVLVKRSILFADDDDSDSHFDHSLPEIEVRVSGKDVLIRTQSDKHSGLAAVILSELEKLHFMVQSSSFLPFGNNKIDVTIIAQMNKEKCMTAKDLLGRLREALSQLI

>C.cajan_18198-07_Cc

MEDSLENWISQMDEVLNESDRAFDEEEFLRGILEHEREHPSNSSTNSSVSIEEATTSLRGRSGTLKSNSSNSIKSFKTSCATYLLSFDNSSAEPITHIEPSPKGLGLGCSNKRTAVKELEEWKPQPKKKIRRSSDSQHHIIAERKRRQELTGSIIALSAIIPGLKKMDKAYVLGEAINYTKQLQERVKELENQNKDKRVDSATLVRKSQSNDNGETNTESLFEVEVRVLDEEVFIGIHCEKQKDTVFKIHALLGKLHLSVNTSSVLPFGSSTLVINIIAQMDEGYKMTMDELVKKLREYLLEVYDSNNGPC

>C.cajan_24883-07_Cc

MEDPTLFHQYPMDPFAFQLEDVDFESLSASPMSSSSHKRFNSESTQNSSLTQSPEQSVAPARPTKQPKTWSAYGSDMMAPKVSSSKIISFENSNASSVSSRQFYNLDAAKLKKPKIETGYGENFDFTAMVSQGLYDDNSFLDYDNREKKLMAATTTTRNPIQAQDHVIAERKRREKLSQRFIALSAIVPGLKKMDKATVLEDAIKYVKQLQERVKTLEEQAVDKTVESAVFIKRSVVFTGDESSSSGEESDQSLPEIEARISGKQVLIRIHCDKHSGQAATILKELEKHNLTVQSSSFLPFGNNTLDISIVAQMNKEYSLTAKDLIRSLSQCLRQL

>C.cajan_24887-07_Cc

MSSLATTSHHTKDHIIAERMRREKISQQFIALSALIPDLKKMDKVSVLGEAIRYVRELKGQVKLLEEQSKRKSEDSLVFVKRPQDEDVSDTSSNSCEFGNFDDSSKTNLSLPEVEARVSKKNVLIRILCKKENVVLVNIFREIEKLHLSVISSSALSFGSSVLDTTIVAEMEDEFNMNVEELARNLRVGLMQFV

>C.cajan_24893-07_Cc

MSNSSTDESNFDFERPTKLLKTTSSSCNSDSSNITKTLSPKLSPSSSFSSFQSQILSFDNPNSSSASNTQFYGFDCALNPTQTDLVSVPVPQLGKQRLPTQTPKGSPKTQNFETKPSHGKRSPAHAQDHIMAERKRREKLSQSFIALAALVPGLKKMDKASVLGDAIKYVKELKERLAVLEEQSKKTRAESVVVLNKPDLSGDDDSSSCDESIGADHSVSESLFEVESRVSGKEMLLRIHCQKQKGLLVKLLAEIQSYNLFVVNSSVLPFGDTILDITIVAQMGESFNLTTKELVKNLRLAAFKAMA

>C.cajan_28075-07_Cc

METSPTGNERPAKQLRNNNWNHNKSQQISDTQFASCSNLLSFVNSNYTSELGVVKPKVEMVCPKINNSTLADMLISQGTLGNQNYVFKASQEAKNIETRPKLSQPQDHIIAERKRREKLSQRFIALSALVPGLKKMDKASVLGEAIKYLKQMQEKVSALEEEQNRKRTVESVVIVKKSLLSSEAEDSSSSDTGGTVDEALPEIEARFCERNVLIRIHCEKNKGVIDKTISEIEKQHLKVINSSAMTFGSFILDITIIAQMDTEFCMTVKDLVRSLRSAFSYFV

>C.cajan_34336-07_Cc

MVPATPEPSSNNSTTLPAKRALGPEAVARPNQGAKKVRSSSQTVDHIMAERRRRQELTERFIALSATIPGLNKTDKASVLRAAIDYVKELKEQIQELEKQDKTRSLLKKRDPSNIPEIEARVLGKEVLIEIHCEKQNGIQFILLDHLENLHLTVTASSVLPFGNSALSITITAQMGEAYKMTVDDLVKNIRQVLPDSHVLSDSDPY

>C.cajan_34338-07_Cc

MTERKRRRELTERFIELSATIPGLKKIDKATILSEAITHVKRLKERVRELEEKCKRTKVESVSFVHPRSLIATDIGTTSGEMDSDECYKTKEALPTVEARVFKKDVLLRIHCKIQSGILIKILDHLNSLDLCTTSNNVMPFGSSTLDISIIAQMGDKFSVTMNDLVKNLRLAILQSSEVQQ

>C.cajan_37903-07_Cc

MAERKRRQQLTERFIALSATIPGLKKTDKSSILCEAINYVKQLQERVTELEERNNRVKESMIILKKTSPCVNEDTITTSGETNSEDSYSRASEMLPDVEARVMENEVLIEIHCEKEDGVQLKILDQLENLDLSVTASSIMPFGNSTLGITIIAQMGDAYGMTVNDLVKNLRQVLLSHMNNNTDPY

>C.cajan_40927-07_Cc

MPCNLSFEDSTLVPNVPKKTCQYFHGEHSQEKPQNIKPKKGRSCSQTHEHIMAERKRRENITKMFIALSALIPGLKKMDKASVLSTAIEYVKHLQQRVKVLEQEKKRKIETIGCFKTNKTNVADDYVSCTYDVLDDKPIKICPKVEARVSGKDVLIKVMCEKQKGIVGKLLAKVEAHDLSVVCTNVLPFGNSALIITTIAKVHTIASIHVKKYILKLNKIVSPKQKIILTF

>C.cajan_42628-07_Cc

MDDGCDIFNEHDIVSAEEELKHNIGNASCLEENTGNTSSEKPMFAFHNLSFEDTIEVPNITSKSKRIEPENGKCNKRVRNSSETNDHVIAERKRRENLTRLFIALSAIIPGLKKMDKLSILNNTIDRVKYLQNRIKVLEEENQKRRMESMTTLANKKPNVNVSDNFFGISNGLDRPSKTFPTVQVSISTKDVIIKVICDKRNGIVRKLLATLAIHNLSVVCSNVLPFGSYALNISIIAKVDRQFIMTMDDYLLKNLKEDLLKCCILQQ

>C.cajan_44447-07_Cc

MDSLISREVGDDVNEFSFDDEEFGFGGGDDEGDSFSETIDPKMTDTKKRQQELTKRFLALSATIPGFKKMDKTSILDKASSYVRQLEQRVRELEQEVQSNICSNNNGSTTSNEVNSNYHYSEANEISPEVKVRVLQKDVLIIIHCEKQKGIMLKVLSYLENINLSVVNSSVLRFGKSTLDITITAQMGDGYKMSVDELVKTLRVAILTQ

>C.cajan_45328-07_Cc

MNDDSACTNATIAAIGPKEECYNSYLKSREEESGSCYELELEGREKSRGTKRARSSYETQYHIMSERKRRQDIAQKFIALSATIPGLKKINKASVLGEAINYMRQLQQRIAMLEKGINNNLSMKSLIITKSCETNLCTYYRANEVLPEVEARGLEKEILIRIYCEKRKGIMIKLLALLKQVHLSIAYSTVLPFGNSFLNIIIIAQMSEKYKLSVKDLVMTLKQGF

>XP_004485645.1-08_Ca

MSSHMEISSIRGLPELGIMEDPNFLHQWNHHISSIDTFGDALQKHPNFNTKTSMETLLAAGSERPMKQLKNNSWNYNNSPQTSDTHQYDNSCCNLVSFVDSNYTSNQLGLLKPKSEMVCPKIDNATLANMLINQGNLGNQNHVFKAFQKAKDVDTRPNKLSQAHDHIVAERKRREKLSQRFIALSALVPGLKKMDKASVLGDAIKYLKQMQEKVNVLEEEQKRKKTVESVVIVKKSQLSNDGEDSCSDTDNSTFDETLPEIEARFCERNVLIRLHCLKSQGVIEKTISEIEKLNLKVNNSSALTFGNFTLDITIIAQMDVGFCMTLKDVVRKLRSAYSSFM

>XP_004486628.1-08_Ca

MDQSCQNSPLHLEVCDLENMAEQCQNSIDDDDEFLRNIFLQQPEEAKSSSESENQLQYLQLQNNNNRSEKRVHNSKMTTPRTFIISFDKSTIIPAATVEVEEEPNKRMERRSGSKLPNSPLSENKKSMDSKSKANQGGKKSRSDSQYLDHIIAERKRRLELTQKFIALSATIPGLKKTDKTTILGEAISYVKVLKERVRELEERNKMKTESTIILNKNDFCSNDTNLEDWCELLPDVKVRVLENEVLIEIHCEKQNGIEIKILDLLENNLHLLVTASSVLPFGNSTLGITIIAQMGVAYKVTVNDLVKPLRQLLLNIRTNKTDPY

>XP_004486629.1-08_Ca

MEDWWENWFSDLDMNGDNSSLKLNECEMNSCCNYKARATEGRKRVRSCWEIEDHVMAERKRRHEMAERLVALSSIIPGLKKIDKASVLGEAINYVKQLKGRIAILEQQSNNKSKRTKCTKSIISFTQSQYHPPNHNHNLDSNQVLPQIEAICIESEKEVLFIRIQCEKCKSILFTLLVILQNMHLSISSSSVLPFGKNTLNIAIIAKMGEEYKITGEELVKKLRQDLMELYEMQHHVIHASVSKEL

>XP_004489860.1-08_Ca

MSNSSGDVNNFERPSKTLKTNPSNTGYPSSQREDSSFPYILYFNNEDHGKSLTSKGSLENQRKETKRNIEENKKTDSITRSSQHNKDHIIAERKRRQNISQHFIALSALIPGLKKMDKASVLRDAIKHVKQLQEQVKLLEEKNQRLQNVEYVVYVESNSGNGKRSLPEVEARMSEKNVLIRIHCEKQKGVLVNIIKEIENLNLSITSATSLLFGTTKLLDITVIAEMDEEFSLCVQELVRNLRVSFLQFME

>XP_004492885.1-08_Ca

MEDLLESLSSYMEMEDDELNQNNSSIDDEQEFLKDIMLEQPECESYSYIFSNKIQNNNSTNVAIINVEGNATSPTNSILPFDEKSNSSNSIMSLERCVCSPATYLLSFDNSSVEPIIEQMSNKRSHDGVEFEPKVNQATKRVKRESEIQDHLMAERKRRKELTESIIALSAIIPGLKKMDKCYVLNEAINYTKHLQQRIKELENQNIDKRVKDPAIFIWKSQASSNKSTTSTYCERNTELLLEVEARVLEKEVLIKIHCENQNDIVLKIHELLEKFNLTITTSSILPFGASILVINIFAQMDEENSMTMDDLVKNLRKHVLEAHDRQ

>XP_004504883.2-08_Ca

MENISFIRGFPDLEILEDPSFFLHQWHSNSIDESNSLPIAAAFGDTLQHHSYIYPNFNHRTSVETAQTLEAQFISYPNLLSFDDSYQINQFGLVKPKDEMVCPKNISKNSSDMISKGTLEAKKIATRPKLSQPQYHIIAERKRREKLSERFIALSALVPGLQKMDKATVLGDAIKYLRQLQEKVRALEEEQNMKKNMESVVVVKKSQLRDDVENSSSESDGSFDEELPEIEARFCDRNVLIRVHCEKSNGVLEKIIHEIERLHLKVTNSSVMTFGSCALDITIIAQMDMEFCMTVKDFVRNLRSAFTSFM

>XP_004511793.1-08_Ca

MEKLNTSSNELATSWLSNLLMEEIDDCNLFQQCQPNLFDDEDFISHDIASVLHQEENLQQQQQPLSSESYSSYNLERPNKKLKTKNTLQDLSPVSSPSSTTSQIMSFEKSKSFSSINEVVMFQTQKGSLQKNNFVETMNSQGQGTKRSMANNQEHIIAERKRREKLSQSLIALAALIPGLKKMDKASVLGDAIKYVKELQERLKVLEEKKKDSPIESVVTVNKPPLSCESSSEEVASGNNEHLPHVEVRVSDKDVLIRIHCHKQMGLLLKILVEIQNLHLFVVNSSVLPFGDSILDITIVAQKGTGYKLSRNDLVKNIRVAALRSMS

>XP_004511794.1-08_Ca

MTSTYESWTSWLCDLEPEDYNFVNESNTNKVHESFHFHHEIDIITPLPQDHNSDSHYSSTMSNSSGDANSFERPSKTLKTNPSNTGYPSSQKKDSSFPYILSFNNENPEPEPILNIDSSSTLKPKAKSLNHGKSLTSNKGSLENQKKETKRKKNDSFTRSSQHNQDHVLAERKRREKLSQQFIALSALIPGLKKMDKASVLSDAVNHVKQLQEQVKVLEEKNTRFKNVESVVYVEKNKNKKSYSHEDVSDTSSNSGYGNCYHESETMPEVEARVSEKNVLIRIHCEKKKEVLVKIMKEIENLHLSVTSSSTLQFGNTNLDITVIAQMDEEFSLSVQELARNLRVGLLKFMEL

>XP_004511795.1-08_Ca

MEDLTFFHQYPIDSLAYPFDNFHSLYESDSSNDNNYFNYDKTTPNCFPLENLDLPRPTKKIKTSNSCSYSSPQLISFEPLNAPQQFYNLDYSDLKPKIEKITGLVSQGSYEEKILSNYDNRANQTMRNSTQAQEHVMAERKRREKLTRSFIALSALVPGLKKMDKASVLGDAIKYVKQLKERLQSLEEQGTKKKAIIVKRSFILPDDNNDIESSKSNQTLPEIEVRVSAKDVLIKIQCDKENGLASTLLGHLQNHNLTLQTTTFLPFGNNILDITILAQMNKENCVTAKDLIGSLRKALLVHN

>XP_004515539.1-08_Ca

MEESWENFHLPTDQLDCGDDYFIDNCDNINTDGDDFLREILLQTPQTFSSESEISEYSFQQVQNDSVTVNVNGVVEVVGNMVKSNSSNSIVSQKQEDLNPKKVPPRRCSLPKTYILSFDNSTMIPATPEPCLDFEGKRDSPLVSNKRSQETKNGERKTNERNGVKRPRSGSQTVDHIMAERKRRQELTERFIALSATIPGLSKTDKASILRAAIDYVKQLQERVNELEKEDKNVGVASVMVLKKTNLCGNNDNEDTNSCETNCDDDCNNILPEIEARVMGKEVLIEIHCKKQNGIELKLLNHLENLQLFVTGSSVLPFGKSAISITIIGQMSEGYKVTGNDLVKSIRKVLLKPQMVCGSDPY

>XP_004516755.2-08_Ca

MEDLWEKWFSSLEMDGDKSPYLDEDMFLVDNMEQTNFFLESANSNINIDNVCVTTIQENMLNNSNTSNSLISQEYNYASNSQQEPSQYILSFEKSIVELSPNSAIATCSSIMGQKTTLNNNVSELPKAKERTKSFRSSSETQDHILAERKRRQVITERFIALSAIIPGLKRTDKAYILREAINYVKQLQEKVKDLENPNKMKKGDSLIFIKNSQASTTEETTSCEEKIDNSKKELPKVEARVIEKEILIEIHCEKQKDIVVRLMVLLQNLHLSLACSSVLPFGNSILKVTIIAQMNDEYCMTMNDLVKTLRQDLLESHDNQKYIFKRVQ

>XP_012573661.1-08_Ca

MEEINNTPMNTSETNKWLSDLEIDEYNLFPEESNLNFLDADEDEFVSHDIASVFEEQTKQQCLTSECTSTTLSNSFTDETCFESFDFDFDFERQTKQLKTIDNDNNIIDTFSTKVSSSSSNSSFQSQILCFDPSINTQQNEVVSVSQPQLGTKGSSKNQNLESQNKRSRANSHDHIMAERLRREKLSVSFIALGALIPGLKKMDKATVLADAIKYVKELKERLEVLEEQRNKRKADSVEKQTKADLCSDDEYSSSDESIEGAAESQFQVDAKVSGKEILIRIHCKKQKGLLVKIISEIQRFQLFVVKNSLLPFGESILDITVIAQMGEGYNLTTKELVKNLREAALKFMSLS

>XP_012573662.1-08_Ca

MIQISSTNYQPELFGMEDPTFFDDQEYTMDSFAFHFDDIDFKPFSASPESFSSHNSNHKRFNSESPNLSVASVQPTKQLKTTWNNTYGSDSMNKNIIPKASNSSPSSKIISFDFDHSNTSSVSSQQFYNMVKKPKTEIAYGKNLNFEAVISQGDYDNKLENKVSTTTTTQRNSIQARDHVMAERKRREKLSQKFIVLSSMIPGLKKMDKATILEDAISYLKQLKERVNTLEEQAAVDRTVESAVFAKRSILFADDDHNSSCDENADQSVPKIEARVSGKDMLLRIYCDKHNGRAATAILCELEKHHLTIQSSCAFPFGNNYLDITIVAKMNKEYCLTIKDLIRSISQVLRQLI

>XP_012573663.1-08_Ca

MAFASKESTTIFFHHNATTIQQTDSNLFCSRQTRLGLYDSAKAKGGLLTLGLLANNKNKNYNMTSIDESWTNWLCDTEPDDYSLINQLDTNTDNVSNLESSNLERPSKLLKKGSSSTYSYILSFENENPQPIKVEQALNPKTKVMNSKNEHKRVNIQEINKKNYSFTRSTTNHTPDHIIAERIRREKISQQFIALSALIPNLKKMDKASVLGDAIKYVKQLKDQVKVLEEQSKRKQSVESIVAVKKLSQLFVDEDVSDTSSYCCNGNSDETLKRNLLLPEVEARLSGKSVMIRILCEKDKVVMVNVYRAIEKFHLSVINASSFSFGSSVLAITIIAQMEDEFNISVQKLAKNLSIELEKLKEW

>Glyma.01G197900.1.p_GmbHLH113-09_Gm

MEIAYNYYLPELQEIEDPTFFDQYQMDSFACPLDDFDFESFSGSPESNSSYHFNSESTPNCFPAESHDQSFTPARPTKRLKNTCASDFISHKVSAFSSSQLISFGHFNAPSPSHASQQFQNLDFDEKASSENMDFAAFVSQGSYEDKSFLSSDNRTNQVGITTRNPIQAQEHIIAERKRRENISKRFIALSAILPGLKKMDKASVLGDAVKYVKQLQERVQTLEEQAAKRTLGSGVLVKRSIIFADDETSDSHCEHSLPEVEVRVSGKDVLIRTQCDKHSGHAAMILSELEKLYFIVQSSSFLPFGNSKTDVTIIAQMNKENCMTAKDLLGRLRQALVGHN

>Glyma.01G198000.1.p_GmbHLH109-09_Gm

MTTREESWTSWLCDLEPEDYNNFVHQSDSNGVGGIFPSSKDSSSASQTEHSSTVSNSSEDDKVFGERPAKTLKIGTSNSSNTEFLSQKKDSSPSYIIFSDNVNQLQAPTLKPKGKVACHGRKGSLENQNFGSVSRSPHHAKDHIIAERMRREKISQQFVALSALIPDLKKMDKASVLGDAIKHVKQLQEQVKLLEEKNKRKRVVESVVYVKKSKLSAAEDVFNTFSNSGDGNSYDISETKTNESFPEVEARVLEKHVLIRIHCGKQKGLFINILKDIENLHLSVINSSILLFGTSKLDITIVAEMDEEFSLSVKELARKLRIGLMQFM

>Glyma.01G198100.1.p_GmbHLH120-09_Gm

MENNNTSMDASEASWLSDLLLQETEDGCNLFRQCHLETLLDDDEELLSHEIASAFENLQQPLSSESNTSYSEAPMNSFTEETSFEKPIKQPKTNASSWNSSFTKHFSLSSSPSSPTSKILSFENSNSSPPNPNNTDQFHGIVVSSALSPKQIKTKGASVSLPHTRKRLSENQNFEAESPKGHRSYKSPSHVRDHIIAERKRREKLSQSLIALAALIPGLKKMDKASVLGDAIKYVKELQERMRMLEEEDKNRDVESVVMVKKQRLSCCDDGSASHEDEENSERLPRVEARVLEKDVLLRIHCQKQKGLLLNILVEIQNLHLFVVNSSVLPFGDSVLDITIVAQMGTGYNLTINDLVKNLRVATLKSMS

>Glyma.03G105000.1.p_GmbHLH98-09_Gm

MEDSWENWISSLEMGEDNNSNGQSHTNSLDRDKLLTGIDLEPPDFSFHQSDHHHHHTYTMSYNHHHSNNNDFPTTSTMGSSSLSYDEDASFDERHGKMLKCNSSNSINISQDVANSHIPSSSSAPSKSTFILSFENSTVEPALHDRVPNYYNNSPNKHFEATCSSLLSSEITLISSDHVITKPKAKQGAKKYRTSSEIKDHIMAERKRRQDLTERFIALSATIPGLKKTDKAYILQEAITYMKQLQERVKVLENENKRKTTYSKIFIKKSQVCSREEATSSCETNSNYRSTPPPLPQVEARMLEKEVLIGIHCQKQKDIVLKIMALLQNLHLSLASSSVLPFGTSTVKVTIIAQMGDKYGMTVNDLVKRLRQDLLKSHDIQESHSKECQI

>Glyma.03G105700.1.p_GmbHLH121-09_Gm

MDESWLKWVSILDTNDYHLFSEYDMNSVEEQLEGENSVDPGELSWENHSYSYLTKESTTLLSNSSSLEEINTGFDMTSLKQDASPQKPKSCILSFEDSTLVPINPKKTCQIYDHGEHSKETQEKPHNRKPLKRGRRFSQTLDHILAERKRRENISRMFIALSALIPDLKKMDKASVLSNAIEYVKYLQQHVKDLEQENKKRKTESLGCFKINKTCDDKPIKKCPKVEARVSGKDVLIRVTCEKQKDIVLKLLAKLEAHNLCIVCSNVLPFGNSALSITSIAMMDHEFSMTVDTYDLVKMLTEELLECCKLQR

>Glyma.05G110600.1.p_GmbHLH111-09_Gm

MMQISSTMYMPEFGMEDPTLFHQYPMDNSVLFQLEDLDFESFSASPKNSSSPKRFNSESTQNSSLTQNPEQYSVTPPRPTKQNKTVSTTWSAYNTHDMMAPKASSSSSSKIISFENSNASSVTSQQLYNVDAASKVKKPKSETGYGENLDFAAAAASQSVYDNNSFLDHYDTREKKAAASLTRNPTQAQDHVISERKRREKLSQRFIALSAIIPGLKKMDKATVLEDAIKYVKQLQERVKTLEEQAVDKTVESAVFVKRSVVFAGDDSSDNDENSDQSLPKIEARISGKEVLIRIHSDKHSGGAAAILRELEKHHLTVQSSSFLPFGNNTFDITIVAKMNMDYCFTAKDLIRSLSRCLRQL

>Glyma.05G110700.1.p_GmbHLH108-09_Gm

MEESWTSCLCDMEPDDYSFIGQSDIKVDDVNGCLVSPHDVATALLEKNQQSLFSRESHSSAAESGLERPFKLPKREQLSQKIASSSTPSSYILSFDNMNPPTIKVESASKPGTKVVNLEKALPSKNEPTRPQENKKMGSFARSSHHTQDHIIAERMRREKISQQFIALSALIPDLKKMDKVSLLGEAIRYVKQLKEQVKLLEEQSKRKNEESVMFAKKSQVFLADEDVSDTSSNSCEFGNSDDPSSKANFLSLPEVEARVSKKNVLIRILCEKEKTVLVNIFREIEKLHLSIIYSSALSFGSSVLDTTIVAEMEDEFNMGVKELARNLRVGLMQFM

>Glyma.05G110900.1.p_GmbHLH118-09_Gm

MMEELNKPMDGSATSWLSDLEMDDYNLFPDECPLNLNLFDDQEFLSQDIASALQEQTQTLQQSLSSECPSKTVSNSSTDETTFDFERPAKLLKTTTSSSCCNSDSSTITKSLSPKLSPSSSFSSFQSQILSFDNPNPTSSSNTTTQFYGFDCTLNPTQNEMVSVSVPNMRKPRFPTQTAKGSPKNQNFETKTSHGKRSPAHAQDHIMAERKRREKLSQSFIALAALVPGLKKMDKASVLGDAIKYVKELKERLTVLEEQSKKSRAESVVVLNKPDLSGDDDSSSCDESIGADSVSDSLFEVESRVSGKEMLLRIHCQKQKGLLVKLLAEIQSHHLFVANSSVLPFGDSILDITIVAQMGESYNLTTKELVKNLRVAALKILS

>Glyma.07G027100.1.p_GmbHLH97-09_Gm

MGEPCQQYWFSDMEIRDFDFFNQSQNHKIGSLDRDEDRIFREIMMHQPVFSSDSDTHSPKILSKSIHGGDSGYNTYTALANNKSSNSNLVMKSNSSNFIFSSQHAPPEKPSTATVASPTAYILSFGDSTLVAATRQNYGGKQPYHEEVVVGSGGVCLPSKGVSEKHDVEPTTANQTTKRSRSSAETLDHIMTERKRRRELTERFIALSATIPGLKKIDKATILSEAITHVKRLKERVRELEEQCKRTKVESVSFVHQRPHITTDKGTTSGAMNSDEWCRTNEALPTVEARVFKKDVLLRIHCKIQSGILIKILDHLNSLDLSTISNSVMPFGSSTLDISIIAQMGDKFKVTMNDLVKNLRLALLQSSEVQ

>Glyma.07G027200.1.p_GmbHLH102-09_Gm

MEESWENWHLHMETGCENDFLGKCHGTDDDEFFRDILQQPPPAAFSSESESDHSFLAVQNTSSIPNDGAVLSPRNYLVKSSSSNSLVSQNASKRPRRLSSPRTYILSFDNSTMVPATPETRPRSSNNSPLPAKRALESPGPVARRPNQGAKKIRTSSQTIDHIMAERRRRQELTERFIALSATIPGLNKTDKASVLRAAIDYVKQLQERVQELEKQDKKRSTESVIFIKKPDPNGNDEDTTSTETNCSILPEMEARVMGKEVLIEIHCEKENGVELKILDHLENLHLSVTGSSVLPFGNSALCITITTQMGDGYQMTVNDLVKNLRQLFSKSHVLSDSDPY

>Glyma.07G027300.1.p_GmbHLH331-09_Gm

MLSSLPDMMEDSWENWLSHLGMNDDFSCTNAVSSEEMVRLNNDSSNVVAAIGPKVEYTDSYLKSSEEKLGSCYWRKRGVENHELEAKARDNERGTKRARTSSETQYHVMSERKRRQDIAEKFIALSATIPGLKKVDKATVLREALNYMRQLQQRIAVLEKGSNNKSIKSLIITKSRLCSASCETNSNEVLPQVEARGLEKEVLIRIYCEKRKDIMLKLLALLKDVHLSIASSSILQFGNSILNIIIIAQMSEKYNLTVNDLVKTLKQIF

>Glyma.07G027400.1.p_GmbHLH105-09_Gm

MEESGENWPSDNSYLDEVNDEFGSEDEEGGSFSGMGETDRKKRKRELAERFLALSATIPGFTKTDKTSILANASSYVKQLQQRVRELEQEVQSNVSSNEGATSSCEVNSSNDYYSGGGPNEILPEVKVRVLQKDVLIIIHCEKQKGIMLKILSQLENVNLSVVNSSVLRFGKITLDITIIAKMGEGYKMTVDELVKTLRVAIS

>Glyma.07G116700.1.p_GmbHLH99-09_Gm

MEDSWQNWISSLEMGDDNSNGQSHINSLDQDKLLTGIVLEPEPPNFSFHQSVHHHHSSTMSQNNHHHSNNNVLVPTTSTMGSSTLSYDEDASFDERHGKMLKCNSSNSITISQDIVANSQIPSKSTFILSFENSTVEPALHDRPNYNNSPKHFEATCSSLLSSEITLNSDYIITKSEAKQGAKKHRTSSEIKDHIMAERKRRRELTERFIALSATIPGLKKTDKAYILREAITYMKQLQERVKELENENKRKTTYSRIFIKKSQVCSREEATSSCETNSYRSTPPLPQVEARVLENEVLIGIHCQKQKDIVLKIMALLQSFHLSLASSSVLPFGTSTLKVTIIAQMGDKYGMAVNDLVKTLRQDLLKSHDIQKSYSKECQI

>Glyma.07G117500.1.p_GmbHLH123-09_Gm

MEESPERWLRFLEMDECNLFGHMHSLVEVFKGENLVQQEKSCKSHPCDSNITKESTTILLRNPINDSSSLEKNPGFDNRSSLKQHASQDEPLFSSSSMPYTLSFEDSTAVPYVLNKTCQCYHGENSKETQEEPKNNRKSKRGRSSSEIQDHIMSERKRRENIAKLFIALSAVIPVLKKTDKASVLKTAIDYVKYLQKRVKDLEEESKKRKVEYAVCFKTNKYNIGTVVDDSDIPINIRPKIEARVSGKDALIKVMCEKRKDIVAKILGKLAALNLSIVCCNVLPFANSALNITCIAQMDHEFTMTLDDLVKILTEDLFDCCN

>Glyma.07G117600.1.p_GmbHLH122-09_Gm

MDEPWLKWVSILDTNDYHLSTECDMNSVEEPLEGENSVDPGELSWENHSPNSYLTKERTTLLSNSSSLEEINTSFDMTSLKQHASPQKPKSCILSFEDSTSVPITSKKTCQLYHGEHSKETQEELPNRKPLKRDTSFDHIMAERKRRENISRLFIALSALIPGLKKMDKASVLYNAIEHVKYLQQRVKDLEKDNKKRKTESVGCFKINKTNVADNVWACDDKPIKICPKVEARVSGKDVVIRVTCEKQKNILPKLLAKLEAHNLSIVCSNVLPFGNSALSITSIAKMDHEFSLTVDTYDLVKTLTGELLECSKMQR

>Glyma.07G185300.1.p_GmbHLH116-09_Gm

MEISSIRGLPDDMEQEIMEDATFLHQWHLSSIDDPNLLPIAAAFGETLQHHAFSTYPNFNPKTSMETTLADDERGTKHHRNISLNPNSKSAQTSSETQFVSFPNLFSFVDSNHTTPPPDTISQGTLGNHNNYVFKACQEAKKTGKRYKHSQPQDHIIAERKRREKLSQRFIALSALVPGLQKTDKASVLGDAIKYLKQLQEKVNALEEEQNMKKNVESVVIVKKCQLSNDVNNSSSEHDGSFDEALPEIEARFCERSVLIRVHCEKSKGVVENTIQGIEKLHLKVINSNTMTFGRCALDITVIAQMDMEFCMGVKDLVRNLRSAFTSFM

>Glyma.08G063900.1.p_GmbHLH334-09_Gm

MEDPTFLHQWHLSSIDDLNLLPIAAAFGETLQHHAFTYPNSNPKTSMETALADIERDTKHHNKNNITQTSSETQFVSFPNLFSFVDSNQTTPPDSISQGTLLGNHNNYVFKACQEAKKTGKRYKHSQPQDHIIAERKRREKLSQRFIALSALVPGLQKTDKASVLGDAIKYLKQLPEKVKALEEEQIMKEILELC

>Glyma.08G215300.1.p_GmbHLH106-09_Gm

MEESGENWPSDSYLDDFVNDDLGFDDEEFGSEDDSFSGMGETDRKKRQRELTERFLALSATIPGFKKTDKTSILANASSYVKQLQQRVRELEQLQEVQSNVTSNEGATSSCEVNSSSNDYYCGGGGPNEILPEVKVRVLQKEVLIIIHCEKHKGIMLKILSQLENVNLSIVNSSVLRFGKSTLDITIVAQMGEGYKMTVGELVKTLRVAILTH

>Glyma.08G215400.1.p_GmbHLH327-09_Gm

MLSLLPDMMEDSWENWLSHLEMNDDFGCTNAVSFDEMVRFNNDSSNVVAAIGPKVEYTDSYLKSSEEEFGTSCYWPKRGVENNHELEAKTIRDNDRGTKRARTSTETQYHVMSERKRRQDIAEKFLALSATIPGLKKLDKATVLREALNYMQQLQQRIAVLEKAGGNKNKSIKSLIITKSRLCSASCETNSISEVLPEVEARGLGKEVLIRIYCEKRKGIILKLLALLKDLHLSIASSSVLPFGNSILNIIIIAQMSEKYNMTVNDLAKSLKQIF

>Glyma.08G215500.1.p_GmbHLH103-09_Gm

MEEPWENWHLHMEMDCGNDFSGKCHGTHDDEFLRDILLQQPPAAFSSESESDHSFRPVVQNTTITNDGAVKSNSSQISSKRPRPLSTPRTYILSFDNSTMLPASPEPRLRSSNNNSPWPPESPGPEPRRPITGGAKKTRTSSQTIDHIMAERRRRQDLTERFIALSATIPGLSKTDKASVLRAAIDYLKQLQERVQELEKQDKKRSKESVIFNKKPDPNGNNNEDTTTSTETNCSILPEMEVRVLGKEVLIEIHCEKENGVELKILDHLENLHLSVTGSSVLPFGNSSLCITITAQMGDGYQMTMNDLVKNLRQVLSKSHLLSDSDPY

>Glyma.08G215600.1.p_GmbHLH96-09_Gm

MGEPCQKYWFSDMEIQDIDFFNQSHNHKIESLDHDEDQIFREIMHQPVFSSDSDTHLPSKIQSKSIHGGVSSGYNTYTVLANNKSNNSNLAMKSNTSNFIFSSQLHAPPEKLPATASTSPTAYILSFDDSTVVAATRQNYGEKQPYHQEVVLGSGGACLPSKGVSEGHDFEPKAKPTTKRSRSSAETLVHIMTERKRRRELTERFIALSATIPGLKKIDKATILSEAITHVKRLKERVRELEEQRKKTRVESVSFVHQRSHIATVKGTTSGAMNSDECCRTNEALPTVEARVFKKDVLLRIHCKIQSGILIKILDHLNSLDLSTISNSVMPFGSSTLDISIIAQMGDNFNVTTMNDLVKNLRMTLSQSSEVQVQQ

>Glyma.11G043600.1.p_GmbHLH119-09_Gm

MDDASEASWLSDLMLKETEDCNLFRHSHLETLLDDDDELLSHEIASAFENLHQPLYSETFERSTKQPKTNASSSPSSPTSKILLSFDNSSDSAALSPNQIKNKGVSVSVSLPQTRKRSSENHNFQTESPKGPRSYKSPSYARDHIIAERKRREKLSQSLIALAALIPGLKKMDRASVLGNAIKYVKELQERLRMLEEENKVMVNKAKLSCEDDIDGSASREDEEGSERLPRVEARVSEKDVLLRIHCQKQKGLLLKILVEIQKFHLFVVSSSVLPFGDSILDITIVAQMEKGYNLTINDIVKNLRVATLKSMS

>Glyma.11G043700.1.p_GmbHLH112-09_Gm

MMEIANHYYLPELGIEDPTLFDQYPMDSFACPLDDFDFESFSGSPESNSSYQFNSESTPNCFPAESPDQSFVPARPTKRLKTFNTSNTCASDIISHKVSASSSSQVISFDHFNAPSDASSLQFHRNLDFDVKPKIEKASSGNMDFAAFVSHGSYVDKTFLSSDTNQVGITSRNPIQAQEHVIAERKRREKLSQRFIALSAILPGLKKMDKASVLGDAIKYVKQLQERVQTLEEQAAKRTAGSRVLVKRSILFADDENSDSHCEHSLPEIEVRVSGKDVLIRTQCDKHSGHAAMILSELEKLHFIVQSSSFLPFGNNNTDVTIIAQMNKENCMTAKDLLGRLRQALKQFI

>Glyma.13G253500.1.p_GmbHLH114-09_Gm

MIDFNACTWDVVLFDILIRLIASAVFHFMRTSHMEISSIRGLPEMGIIEDPNFLHHWQLNSIDTTSLTGAPFGEILQKHSFSDNSNFNPKTSMETSPTGIERPAKQLRNNSWNHNKSQQQTPETQFASCSNLLSFVNSNYTSELGLVKPKVEMVCPKINNNTLADMLISQGNLGNQNYLFKASQEAKKIETRPKLSQPQDHIIAERKRREKLSQRFIALSALVPGLKKMDKASVLGEAIKYLKQMQEKVSALEEEQNRKRTVESVVIVKKSRLSSDAEDSSSSETGDTFDEALPEIEARFYERNVLIRIHCEKNKGVIEKTISEIEKLHLKVINSSALTFGSFILDITIIAQMDMEFCMTVKDLVRSLRSAFSYFV

>Glyma.13G368500.1.p_GmbHLH100-09_Gm

MEENPWGSWSSNMEMDDEDVNESLRETNQFDEEFLRDILQQPEEGGEDLKNSSIMSSLFPTTYILSFDKSAAELLPTETDHRDYSSSQLPSSSNSRANHGTNKKPRSASESLDHIMSERNRRQELTSKFIALAATIPGLKKMDKAHVLREAINYVKQLQERIEELEEDIRKNGVESAITIIRSHLCIDDDSNTDEECYGPNEALPEVEARVLGKEVLIKIYCGKQKGILLKIMSQLERLHLYISTSNVLPFGNTLDITITAQMGDKYNLVVNDLVKELRQVAMMKSCDVQQ

>Glyma.13G368600.1.p_GmbHLH337-09_Gm

MGGDISFFSEEHPNPTLYCVANETHVQTGAKRGRSSWETPTRDHIMSERKRRQLMAERFIALSAIIPGLKKIDKASVLSEAINYVKQLKGRIAVLEQESSNKKSMMIFTKKCLQSHPHCEKNSNHVLPQLQVEAIGLELEREVLIRILCEKPKGIFLKLLTLLENMHLSIVSSNVLPLGKNTLNITIIAQMGEEYNMTGDELMSKLTQDLFKLYEVPQ

>Glyma.13G368700.1.p_GmbHLH329-09_Gm

MEQSWENWPLDMGMEEDRDLRDEEEFLRNILSEPSESEMVTVGSAKRPPRTYILSFDNSTIIPATPEPTSRKRTRQPQNLNLEPIKPNPNTQPGKRGRSCSQTLDHIMAERKRRQELTQKFIALSATIPGLKKTDKSSILGEAIDYVKQLQERVTELEQRNMRGKESMIILKKSEVCNSSETNSEDCCRASEMLPDVEARVMENEVLIEIHCEKEDGVELKILDHLENLQLCVTASSVLPFGNSTLGITIIAQMGDAYKMKVNDLVPKLRQVLLNRMNVNTDPY

>Glyma.15G005000.1.p_GmbHLH104-09_Gm

MEQSYSWENWALDKEMGEDEEEFLRDILSKPAFSSESESQAPVVSCSAKSKRAPMTYILSFDNSTITPAPSSPPTLEAQPGKRAKRASHIMAERKRRQQLTQSFIALSATIPGLNKKDKSSMLGKAIDYVKQLQERVTELEQRKKRGKESMIILKKSEANSEDCCRANKMLPDVEARVTENEVLIEIHCEKEDGLELIKILDHLENLHLCVTASSVLPFGNSTLSITIIAQMGDAYKMKVNDLVKKLRQVLLNHTNVNTNPY

>Glyma.15G005100.1.p_GmbHLH101-09_Gm

MEENPWGSWSADMEVDDEVVNESLCHTNLFDEEFLRDILQRPQEGAPNLNNSSIMSSLCAQDIKPTTLFPTTYILSFDNSAALLPTETDRDFSSSKLPSSNSRANHGIKKPGSASESLNHIMSERNRRQELTSKFIALAATIPGLKKMDKAHVLREAINYVKQLQERVEELEEDIQKNGVESEITITRSHLCIDDGTNTDECYGPNEALPEVEARVLGKEVLIKIHCGKHYGILLEVMSELERLHLYISASNVLPFGNTLDITIIAQMGDKYNLVAKDLVKELRQVAMMKSCYVQQ

>Glyma.15G061400.1.p_GmbHLH115-09_Gm

MWLSFFHEFGDISLIASAVFHCMRSSHMEISSIRGLPELGIIEDPNFLHQWQLNSIDTTSLKGAAFGDILQKHSFSDNSNFNPKTSMETSQTGIERYAKQLGDNSWNHNKSQQQTPETQFASCSNLLSFVNTNYTSELGLVKPKVEMACPKIDNNALADMLISQGTLGNQNYIFKASQETKKIKTRPKLSQPQDHIIAERKRREKLSQRFIALSALVPGLKKMDKASVLGEAIKYLKQMQEKVSALEEEQNRKRTVESVVIVKKSQLSSDAEDSSSETGGTFVEALPEIEARFWERNVLIRIHCEKNKGVIEKTISEIEKLHLKVINSSALTFGSFILDITIIAQMDMEFCMTVKDLVRSLRSAFSYFV

>Glyma.17G155900.1.p_GmbHLH117-09_Gm

MMEELNKPMDGSATSWLSDLEMDDYNLFPDECPLNLNMFDDREFLSQDIASAFQEQTQTLQQQSLSSECPSKTVSNSSTDETTFDFERPSKLLKTTTSTSSNFDSSTITKTLSPKLSPSSSFQSQILSFDNTNTQFYEFHCTLNPTQNEMVSVSVPQKGKPRFPTQTPKGSPKYQNFETKTSHAKRSPAHAQDHIMAERKRREKLSQSFIALAALVPGLKKMDKASVLGDAIEYVKELKERLTVLEEQSKKTRAESIVVLNKPDLSGDNDSSSCDESIDADSVSDSLFEVESRVSGKEMLLKIHCQKQRGLLVKLLAEIQSNHLFVANSSVLPFGNSILDITIVAQMGESYNLTTKELAKNLRVAALKILS

>Glyma.17G156000.1.p_GmbHLH107-09_Gm

MTSMEESWTSWLCDMEPDDYSFMGQSDIKVDDVNGTLASPHDVATALLEKNQQSSFSTVESLSSAAESGLERPFKLPKIEQLSQKTASSSTPSSYILSFDNTNPPPVTVESASKPGTKVLNLEKVLPSKNEPRRVVTQQNKKMGSFAGSSHHTQDHIIAERMRREKISQKLIALSALIPDLKKMDKVSVLGEAIRYVKQLKEQVKVLEEQSKRKNEESVVFAKKSQVFPADEDVSDTSSNSCEFGNSDDISTKATLSLPEVEARVSKKSVLIRILCEKEKAVLVNIFREIEKLHLSVVNSSALSFGSSVLDTTIVAEMEDEFNMGVKELARNLRVGLMQFM

>Glyma.17G156100.1.p_GmbHLH110-09_Gm

MMQISSNMYMPEFGMEDPTLFHQYPMDSSSALFQLEDLDFESFSASPKNSSSHNKRLFNSESTQNSFHKSPHEQYSVAPPRPTKQNKTVMSTTWSAYNAHHHHHDMVAPKASSSSSSKIISFDQHSNASSVTSQQLYNNVDAAAKVKKPKIETGYGENLDFSAAAAAASQSICDNNSFLDHYDNQDKKAAASTTRNPTQAQDHVIAERKRREKLSQRFIALSAIVPGLKKMDKATVLEDAIKYVKQLQERVKTLEEQAVDKTVESAVFVKRSVVFAGVDSSSSDENSDQSLPEMEARISGKEVLIRIHCDKNSGGAAAILRELEKHYLTVQSSSFLPFGNNTLDITIVAKMNNDYCLTAKDLIRSLSQCLRQL

>Glyma.18G246000.1.p_GmbHLH345-09_Gm

MEDSLENWISLLEMEEYHDGSFDEEEFLREILKEEQQPENLWPQRDQKLSNTSPTVSFDEAISFCGSGTLHKSNSSNSIKSLKRSSSSVSPATTTYLLSFDTSSAKPITLKPSPKLDLALGSSNKRSTSTVTVVKDGCEFEPLMMPQSQARKKVRRSCETQHHIIAERKRRQELTGSIIALAATIPGLKRMDKAYVLREAVNYTKQLQERVKELENQNKVDSATFIRKSQASSHCETNKEISLFEVEARVLDEEVLIGIHCEKQKDIVFKIHALLGKLHLSTTSSTVLPFGTSTLIINIIAQMN

>KHM99702.1-10_Gs

MMEELNKPMDGSATSWLSDLEMDDYNLFPDECPLNLNLFDDQEFLSQDIASALQEQTQTLQQSLSSECPSKTVSNSSTDETTFDFERPAKLLKTTTSSSCCNSDSSTITKSLSPKLSPSSSFSSFQSQILSFDNPNPTSSSNTTTQFYGFDCTLNPTQNEMVSVSVPNLRKPRFPTQTAKGSPKNQNFETKTSHGKRSPAHAQDHIMAERKRREKLSQSFIALAALVPGLKKMDKASVLGDAIKYVKELKERLTVLEEQSKKSRAESVVVLNKPDLSGDDDSSSCDESIGADSVSDSLFEVESRVSGKEMLLRIHCQKQKGLLVKLLAEIQSHHLFVANSSVLPFGDSILDITIVAQMGESYNLTTKELVKNLRVAALKILS

>KHN01297.1-10_Gs

MKSNSSNFIFSSQHAPPEKPSTATVASPTAYILSFGDSTLVAATRQNYGGKQPYHEEVVVGSGGVCLPSKGVSEKHDVEPTTANQTTKRSRSSAETLDHIMTERKRRRELTERFIALSATIPGLKKIDKATILSEAITHVKRLKERVRELEEQCKRTKVESVSFVHQRPHITTDKGTTSGAMNSDECCRTNEALPTVEARVFKKDVLLRIHCKIQSGILIKILDHLNSLDLSTISNSVMPFGSSTLDISIIAQMGDKFKVTMNDLVKNLRLALLQSSEVQ

>KHN01298.1-10_Gs

MEESWENWHLHMETDCENDFLGKCHGTDDDEFFRDILQQPPPAAFSSESESDHSFLAVQNTSSFPNDGAVLSPRNYLVKSSSSNSLVSQNASKRPRRLSSPRTYILSFDNSTMVPATPETRPRSSNNSPLPAKRALESPGPVARRPNQGAKKIRTSSQTIDHIMAERRRRQELTERFIALSATIPGLNKTDKASVLRAAIDYVKQLQERVQELEKQDKKRSTESVIFIKKPDPNGNDEDTTSTETNCSILPEMEARVMGKEVLIEIHCEKENGVELKILDHLENLHLSVTGSSVLPFGNSALCITITTQMGDGYQMTVNDLVKNLRQLFSKSHVLSDSDPY

>KHN01299.1-10_Gs

MLSSLPDMMEDSWENWLSHLGMNDDFSCTNAVSSEEMVRLNNDSSNVVAAIGPKVEYTDSYLKSSEEKLGSCYWRKRGVENHELEAKARDNERGTKRARTSSETQYHVMSERKRRQDIAEKFIALSATIPGLKKVDKATVLREALNYMRQLQQRIAVLEKGSNNKSIKSLIITKSRLCSASCETNSNEVLPQVEARGLEKEVLIRIYCEKRKDIMLKLLALLKDVHLSIASSSILQFGNSILNIIIIAQMSEKYNLTVNDLVKTLKQIF

>KHN01301.1-10_Gs

MEESGENWPSDNSYLDEVNDEFGSEDEEGGSFSGMGETDRKKRKRELAERFLALSATIPGFTKTDKTSILANASSYVKQLQQRVRELEQEVQSNVSSNEGATSSCEVNSSNDYYSGGGPNEILPEVKVRVLQKDVLIIIHCEKQKGIMLKILSQLENVNLSVVNSSVLRFGKITLDITIIAKMGEGYKMTVDELVKTLRVAIS

>KHN01614.1-10_Gs

MDEPWLKWVSILDTNDYHLSTECDMNSVEEPLEGENSVDPGELSWENHSPNSYLTKERTTLLSNSSSLEEINTSFDMTSLKQHASPQKPKSCILSFEDSTSVPITSKKTCQLYHGEHSKETQEELPNRKPLKRDTSFDHIMAERKRRENISRLFIALSALIPGLKKMDKASVLYNAIEHVKYLQQRVKDLEKDNKKRKTESVGCFKINKTNVADNVWTCDDKPIKICPKVEARVSGKDVVIRVTCEKQKNILPKLLAKLEAHNLSIVCSNVLPFGNSALSITSIAKEKDNTSLK

>KHN01615.1-10_Gs

MHSLVEVFKGENLVQQEKSCKSHPCDSNITKESTTILLRNPINDSSSLEKNPGFDNRSSLKQHASQDEPLFSSSSMPYTLSFEDSTAVPYVLNKTCQCYHGENSKETQEEPKNNRKSKRGRSSSEIQDHIMSERKRRENIAKLFIALSAVIPVLKKTDKASVLKTAIDYVKYLQKRVKDLEEESKKRKVEYAVCFKTNKYNIGTVVDDSDIPINIRPKIEARVSGKDALIKVMCEKRKDIVAKILGKLAALNLSIVCCNVLPFANSALNITCIAQGQ

>KHN01621.1-10_Gs

MEDSWQNWISSLEMGDDNSNGQSHINSLDQDKLLTGIVLEPEPPNFSFHQSVHHHHSSTMSQNNHHHSNNNVLVPTTSTMGSSTLSYDEDASFDERHGKMLKCNSSNSITISQDIVANSQIPSKSTFILSFENSTVEPALHDRPNYNNSPKHFEATCSSLLSSEITLNSDYIITKSEAKQGAKKHRTSSEIKDHIMAERKRRRELTERFIALSATIPGLKKTDKAYILREAITYMKQLQERVKELENENKRKTTYSRIFIKKSQVCSREEATSSCETNSYRSTPPLPQVEARVLENEVLIGIHCQKQKDIVLKIMALLQSFHLSLASSSVLPFGTSTLKVTIIAQMGDKYGMAVNDLVKTLRQDLLKSHDIQKSYSKECQI

>KHN01700.1-10_Gs

MMEELNKPMDGSATSWLSDLEMDDYNLFPDECPLNLNMFDDREFLSQDIASAFQEQTQTLQQQSLSSECPSKTVSNSSTDETIFDFERPSKLLKTTTSTSSNFDSSTITKTLSPKLSPSSSFQSQILSFDNTNTQFYEFHCTLNPTQNEMVSVSVPQKGKPRFPTQTPKGSPKYQNFETKTSHAKRSPAHAQDHIMAERKRREKLSQSFIALAALVPGLKKMDKASVLGDAIEYVKELKERLTVLEEQSKKTRAESIVVLNKPDLSGDNDSSSCDESIDADSVSDSLFEVESRVSGKEMLLKIHCQKQRGLLVKLLAEIQSNHLFVANSSVLPFGNSILDITIVAQMGESYNLTTKELAKNLRVAALKILS

>KHN01701.1-10_Gs

MGQSDIKVDDVNGTLASPHDVATALLEKNQQSSFSTVESLSSAAESGLERPFKLPKIEQLSQKTASSSTPSSYILSFDNTNPPPVTVESASKPGTKVLNLEKVLPSKNEPRRVVTQQNKKMGSFAGSSHHTQDHIIAERMRREKISQKLIALSALIPDLKKMDKVSVLGEAIRYVKQLKEQVKLLEEQSKRKNEESVVFAKKSQVFPADEDVSDTSSNSCEFGNSDDISTKATLSLPEVEARVSKKSVLIRILCEKEKAVLVNIFREIEKLHLSVVNSSALSFGSSVLDTTIVAEMEDEFNMGVKELARNLRVGLMQFM

>KHN01702.1-10_Gs

MMQISSNMYMPEFGMEDPTLFHQYPMDSSSALFQLEDLDFESFSASPKNSSSHNKRLFNSESTQNSFHKSPHEQYSVAPPRPTKQNKTVMSTTWSAYNAHHHHHDMVAPKASSSSSSKIISFDQHSNASSVISQQLYNNVDAAAKVKKPKIETGYGENLDFSAAAAAASQSICDNNSFLDHYDNQDKKAAASTTRNPTQAQDHVIAERKRREKLSQRFIALSAIVPGLKKMDKATVLEDAIKYVKQLQERVKTLEEQAVDKTVESAVFVKRSVVFAGVDSSSSDENSDQSLPEMEARISGKEVLIRIHCDKNSGGAAAILRELEKHYLTVQSSSFLPFGNNTLDITIVAKMNNDYCLTAKDLIRSLSQCLRQL

>KHN08182.1-10_Gs

MDESWLKWVSILDTNDYHLFSEYDMNSVEEQLEGENSVDPGELSWENHSYSYLTKESTTLLSNSSSLEEINTGFDMTSLKQDASPQKPKSCILSFEDSTLVPINPKKTCQIYDHGEHSKETQEKPHNRKPLKRGRSFSQTLDHILAERKRRENISRMFIALSALIPDLKKMDKASVLSNAIEYVKYLQQHVKDLEQENKKRKTESLGCFKINKTCDDKPIKKCPKVEARVSGKDVLIRVTCEKQKDIVLKLLAKLEAHNLCIVCSNVLPFGNSALSITSIAMVGAHHYYNDVFRRDQFHVP

>KHN13036.1-10_Gs

MDSFACPLDDFDFESFSGSPESNSSYQFNSESTPNCFPAESPDQSFVPARPTKRLKTFNTSNTCASDIISHKVSASSSSQVISFDHFNAPSDASSLQFHRNLDFDVKPKIEKASSGNMDFAAFVSHGSYVDKTFLSSDTNQVGITSRNPIQAQEHVIAERKRREKLSQRFIALSAILPGLKKMDKASVLGDAVKYVKQLQERVQTLEEQAAKRTAGSRVLVKRSILFADDENSDSHCEHSLPEIEVRVSGKDVLIRTQCDKHSGHAAMILSELEKLHFIVQSSSFLPFGNNNTDVTIIAQMNKENCMTAKDLLGRLRQALKQFI

>KHN13037.1-10_Gs

MDDASEASWLSDLMLKETEDCNLFRHSHLETLLDDDDELLSHEIASAFENLHQPLYSETFERSTKQPKTNASSSPSSPTSKILLSFDNSSDSAALSPNQIKNKGVSVSVSLPQTRKRSSENHNFQTESPKGPRSYKSPSYARDHIIAERKRREKLSQSLIALAALIPGLKKMDRASVLGNAIKYVKELQERLRMLEEENKVMVNKAKLSCEDDIDGSASREDEEGSERLPRVEARVSEKDVLLRIHCQKQKGLLLKILVEIQKFHLFVVSSSVLPFGDSILDITIVAQMEKGYNLTINDIVKNLRVATLKSMS

>KHN19045.1-10_Gs

MEISSIRGLPEMGIIEDPNFLHHWQLNSIDTTSLTGAPFGEILQKHSFSDNSNFNPKTSMETSPTGIERPAKQLRNNSWNHNKSQQQTPETQFASCSNLLSFVNSNYTSELGLVKPKVEMVCPKINNNTLADMLISQGNLGNQNYLFKASQEAKKIETRPKLSQPQDHIIAERKRREKLSQRFIALSALVPGLKKMDKASVLGEAIKYLKQMQEKVSALEEEQNRKRTVESVVIVKKSRLSSDAEDSSSSETGDTFDEALPEIEARFYERNVLIRIHCEKNKGVIEKTISEIEKLHLKVINSSALTFGSFILDITIIAQMDMEFCMTVKDLVRSLRSAFSYFV

>KHN22139.1-10_Gs

MEEYDDVLNESHHGSFDEQEFLREILEEQPENLSPQRENICSTSPTNSTVSFDEAISFNYASGSTLHKSNSSNSIKSLKRSSSSSVSPASTTTTTTTTYLLSFDTSIVEPITLKPSPKLDVVKDGSEFDALMPQTQPKKKVRRSCETRHHIIAERKRRQELTGSIIALSATIPGLKKMDKAYVLREAVNYTKQLQERVKELENQNKVDSATFIRKSEASSDKNTGNCETNKEISLFEVEARVLDKEVLIGIHCEKQKDIVFKIHALLRNLHLSTTSSTVLPFGTSTLIINIIAQMNGEYSMTKDDLVKKLREYLLEVYDMQ

>KHN22140.1-10_Gs

MEDSRMDLLSQWEMGDDDFFNNQIHMISFTEEKSSKDMIFQQAFFSSSSSSSSLQSNLKPCSPFPTGGYVLSFDKPVVEPLYPKKHHSPKSLAQSQYCVEPKAFPRTRPRVHILAERKRREELTKSIVALSATIPGLKKTDKVNVVREAVSYVKQLQERVKELENQKRKESMNSIILNKHRPLSINDQATHGFVDVNEELLEVKVTVLDKEVLIGIYCEKQRQRLLKILSLLDNLHLSITHTSVLPFGTSTLKITIIAQMMKVISMTSQNLWNVERQLIGIMNSVLLEKH

>KHN22417.1-10_Gs

MDDWLSDQDMGGDNSFFSEEHRNPTLYCVANETRNQLRRSKDTDTGSHYVREKKETANGRDIHSAFSYYTWLEQVGQGLLGAGEDRMEQSYSWENWALDKEMGEDEEEFLRDILSKPAFSSESESQAPVVSCSAKSKRAPMTYILSFDNSTITPAPSSPPTLEAQPGKRAKRASHIMAERKRRQQLTQSFIALSATIPGLNKKDKSSMLGKAIDYVKQLQERVTELEQRKKRGKESMIILKKSEANSEDCCRANKMLPDVEARVTENEVLIEIHCEKEDGLELIKILDHLENLHLCVTASSVLPFRNSTLSITIIAQMGDAYKMKVNDLVKKLRQVLLNHTNVNTNPY

>KHN22418.1-10_Gs

MEENPWGSWSSDMEVDDEVANESLCHTNLFDEEFLRDILQRPQEGAPNLNNSSIMSSLCAQDIKPTTLFPTTYILSFDNSAALLPTETDRDSSSSKLPSSNSRANHGIKKPGSASESLNHIMSERNRRQELTSKFIALAATIPGLKKMDKAHVLREAINYVKQLQERVEELEEDIQKNGVESEITITRSHLCIDDGTNTDECYGPNEALPEVEARVLGKEVLIKIHCGKHYGILLEVMSELERLHLYISASNVLPFGNTLDITIIAQMGDKYNLVAKDLVKELRQVAMMKSCYVQQ

>KHN31892.1-10_Gs

MEQSWENWPLDMGMEEDRDLRDEEEFLRNILSEPSESEMVTVGSAKRPPRTYILSFDNSTIIPATPEPTSRKRTRQPQNLNLEPIKPNPNTQPGKRGRSCSQTLDHIMAERKRRQELTQKFIALSATIPGLKKTDKSSILGEAIDYVKQLQERVTELEQRNMRGKESMIILKKSEVCNSSETNSEDCCRASEMLPDVEARVMENEVLIEIHCEKEDGVELKILDHLENLQLCVTASSVLPFGNSTLGITIIAQAPTFTPFHFSSIDEKISCVVVDGGLVMNNPAVAVVTHVLHNKCDFPSVNGMEDLLVLSIGNRAQAKRMNNAGECSTSTVVDIALDGVFETTDQMLGNAFCWNRTDYVRIQDEEILHAVHRWEIVLVVEDVRDGSCCQIVHHQAIVDGGEVEGSEEARGRRRCEVRSSQQ

>KHN31893.1-10_Gs

MGGDISFFSEEHPNPTLYCVANETHVQTGAKRGRSSWETPTRDHIMSERKRRQLMAERFIALSAIIPGLKKIDKASVLSEAINYVKQLKGRIAVLEQESSNKKSMMIFTKKCLQSHPHCEKNSNHVLPQLQVEAIGLELEREVLIRILCEKPKGIFLKLLTLLENMHLSIVSSNVLPLGKNTLNITIIAQMGEEYNMTGDELMSKLTQDLFKLYEVPQ

>KHN31894.1-10_Gs

MDDEDVNESLRETNQFDEEFLRDILQQPEEGSEDLKNSSIMSSLFPTTYILSFDKSAAELLPTETDHRDYSSSQLPSSSNSRANHGTNKKPRSASESLDHIMSERNRRQELTSKFIALAATIPGLKKMDKAHVLREAINYVKQLQERIEELEEDIRKNGVESAITIIRSHLCIDDDSNTDEECYGPNEALPEVEARVLGKEVLIKIYCGKQKGILLKIMSQLERLHLYISTSNVLPFGNTLDITITAQMGDKYNLVVNDLVKELRQVAMMKSCDVQQ

>KHN33594.1-10_Gs

MRSSHMEISSIRGLPELGIIEDPNFLHQWQLNSIDTTSLKGAAFGDILQKHSFSDNSNFNPKTSMETSQTGIERYAKQLGDNSWNHNKSQQQTPETQFASCSNLLSFVNTNYTSELGLVKPKVEMACPKIDNNALADMLISQGTLGNQNYIFKANQETKKIKTRPKLSQPQDHIIAERKRREKLSQRFIALSALVPGLKKMDKASVLGEAIKYLKQMQEKVSALEEEQNRKRTVESVVIVKKSQLSSDAEDSSSETGGTFVEALPEIEARFWERNVLIRIHCEKNKGVIEKTISEIEKLHLKVINSSALTFGSFILDITIIAQMDMEFCMTVKDLVRSLRSAFSYFV

>KHN38012.1-10_Gs

MEESGENWPSDSYLDDFVNDDLGFDDEEFGSEDDSFSGMGETDRKKRQRELTERFLALSATIPGFKKTDKTSILANASSYVKQLQQRVRELEQLQEVQSNVTSNEGATSSCEVNSSSNDYYCGGGGPNEILPEVKVRVLQKEVLIIIHCEKHKGIMLKILSQLENVNLSIVNSSVLRFGKSTLDITIVAQMGEGYKMTVDELVKTLRVAILTH

>KHN38013.1-10_Gs

MLSLLPDMMEDSWENWLSHLEMNDDFGCTNAVSFDEMVRFNNDSSNVVAAIGPKVEYTDSYLKSSEEEFGTSCYWPKRGVENNHELEAKTIRDNDRGTKRARTSTETQYHVMSERKRRQDIAEKFLALSATIPGLKKLDKATVLREALNYMQQLQQRIAVLEKAGGNKNKSIKSLIITKSRLCSASCETNSISEVLPEVEARGLGKEVLIRIYCEKRKGIILKLLALLKDLHLSIASSSVLPFGNSILNIIIIAQMSEKYNMTVNDLAKSLKQIF

>KHN38014.1-10_Gs

MEEPWENWHLHMEMDCGNDFSGKCHGTHDDEFLRDILLQQPPAAFSSESESDHSFRPVVQNTTITNDGAVKSNSSQNSSKRPRPLSTPRTYILSFDNSTMLPASPEPRLRSSNNNSPWPPESPGPEPRRPITGGAKKTRTSSQTIDHIMAERRRRQDLTERFIALSATIPGLSKTDKASVLRAAIDYLKQLQERVQELEKQDKKRSKESVIFNKKPDPNGNNNEDTTTSTETNCSILPEMEVRVLGKEVLIEIHCEKENGVELKILDHLENLHLSVTGSSVLPFGNSSLCITITAQMGEGYQMTMNDLVKNLRQVLSKSHLLSDSDPY

>KHN38016.1-10_Gs

MFNESLFSAILISIYYQRYSFSIISLESNPQSQLPYTFFHSLPMGEPCQKYWFSDMEIQDIDFFNQSHNHKIESLDHDEDQIFREIMHQPVFSSDIDTHLPSKIQSKSIHGGVSSGYNTYTVLANNKSNNSNLAMKSNTSNFIFSSQLHAPPEKLPATASTSPTAYILSFDDSTVVAATRQNYGEKQPYHQEVVLGSGGACLPSKGVSEGHDFEPKAKPTTKRSRSSAETLVHIMTERKRRRELTERFIALSATIPGLKKIDKATILSEAITHVKRLKERVRELEEQRKKTRVESVSFVHQRSHIATVKGTTSGAMNSDECCRTNEALPTVEARVFKKDVLLRIHCKIQSGILIKILDHLNSLDLSTISNSVMPFGSSTLDISIIAQMGDNFNVTTMNDLVKNLRMTLSQSSEVQVQQ

>KHN40506.1-10_Gs

MEDSLENWISQLEMEEYHDGSFDEEEFLREILKEEQQPENLWPQRDQKLSNTSPTNSTVSFDEAISFCGSGTLHKSNSSNSIKSLKRSSSSVSPATTTYLLSFDTSSAKPITLKPSPKLDLALGSSNKRSTSTVTVVKDGCEFEPLMMPQSQARKKVRRSCETQHHIIAERKRRQELTGSIIALAAAIPGLKRMDKAYVLREAVNYTKQLQERVKELENQNKVDSATFIRKSQASSHCETNKEISLFEVEARVLDEEVLIGIHCEKQKDIVFKIHALLGKLHLSTTSSTVLPFGTSTLIINIIAQMNGENSMTMHDLVKKLRDYLLEVYDMQ

>KHN40507.1-10_Gs

MGGDDFFNIQSHGHMSPFNEEEYSSGERDMIVQQQVFSPNLSSSSLQSNLELPYSSSPTGNVLSFDNPLVGSLHPQNDSAKSMEQSHCVEPKAYPRTNAQVHILAERKRREELTRCIIALSATIPGLKKTDKVTVLREAVNYVKQLQERVKELENQMRKECVNSIILTKKALICKNDRVEELLEVKVTVLDKEVLIGVHCEKQRKSLLKILSLLNNLHLSITSTSVLPFGSSTLKITIISQMDDEYNMTLDDLVRTLRQRILSLKSHDT

>KHN42602.1-10_Gs

MEIAYNYYLPELQGIEDPTFFDQYQMDSFACPLDDFDFESFSGSPESNSSYHFNSESTPNCFPAESHDQSFTPARPTKRLKNTCASDFISHKVSAFSSSQLISFGHFNAPSPSHASQQFQNLDFDEKASSENMDFAAFVSQGSYEDKSFLSSDNRTNQVGITTRNPIQAQEHIIAERKRRENLSKRFIALSAILPGLKKMDKASVLGDAVKYVKQLQERVQTLEEQAAKRTLGSGVLVKRSIIFADDETSDSHCEHSLPEVEVRVSGKDVLIRTQCDKHSGHAAMILSELEKLYFIVQSSSFLPFGNSKTDVTIIAQMNKENCMTAKDLLGRLRQALVGHN

>KHN42603.1-10_Gs

MRREKISQQFVALSALIPDLKKMDKASVLGDAIKHVKQLQEQVKLLEEKNKRKRVVESVVYVKKSKLSAAEDVFNTFSNSGDGNSYDISETKTNESFPEVEARVLEKHVLIRIHCGKQKGLFINILKDIENLHLSVINSSILLFGTSKLDITIVAEMDEEFSLSVKELARKLRIGLMQFM

>KHN42604.1-10_Gs

MENNNTSMDASEASWLSDLETEDGCNLFRQCHLETLLDDDEELLSHEIASAFENLQQPLSSESNTSYSETPMNSFTEETSFEKPIKQPKTNASSWNSSFTKHFSLSSSPSSPTSKILSFENSNSSPPNPNNTDQFHGIVVSAALSPKQIKTKGASVSLPHTRKRLSENQNFEAESPKGHRSYKSPSHVRDHIIAERKRREKLSQSLIALAALIPGLKKMDKASVLGDAIKYVKELQERMRMLEEEDKNRDVESVVMVKKQRLSCCDDGSASHEDEENSERLPRVEARVLEKDVLLRIHCQKQKGLLLNILVEIQNLHLFVVNSSVLPFGDSVLDITIVAQMGTGYNLTINDLVKNLRVATLKSMS

>KHN46510.1-10_Gs

SYILSFDNMNPPTIKVESASKPGTKVVNLEKALPSKNEPTRPQENKKMGSFARSSHHTQDHIIAERMRREKISQQFIALSALIPDLKKMDKVSLLGEAIRYVKQLKEQVKLLEEQSKRKNEESVMFAKKSQVFLADEDVSDTSSNSCEFGNSDDPSSKANFLSLPEVEARVSKKNVLIRILCEKEKTVLVNIFREIEKLHLSIIYSSALSFGSSVLDTTIVAEMEDEFNMGVKELARNLRVGLMQFM

>KHN47568.1-10_Gs

MMQISSTMYMPEFGMEDPTLFHQYPMDNSVLFQLEDLDFESFSASPKNSSSPKRFNSESTQNSSLTQNPEQYSVTPPRPTKQNKTVSTTWSAYNTHDMMAPKASSSSSSKIISFENSNASSVTSQQLYNVDAASKVKKPKSETGYGENLDFAAAAASQSVYDNNSFLDHYDTREKKAAASLTRNPTQAQDHVISERKRREKLSQRFIALSAIIPGLKKMDKATVLEDAIKYVKQLQERVKTLEEQAVDKTVESAVFVKRSVVFAGDDSSDNDENSDQSLPKIEARISGKEVLIRIHSDKHSGGAAAILRELEKHHLTVQSSSFLPFGNNTFDITIVAKMNMDYCFTAKDLIRSLSRCLRQL

>KHN47580.1-10_Gs

MEDSWENWISSLEMGEDNNSNGQSHTNSLDRDKLLTGIDLEPPDFSFHQSDHHHHHTYTMSYNHHHSNNNDFPTTSTMGSSSLSYDEDASFDERHGKMLKCNSSNSINISQDVANSHIPSSSSAPSKSTFILSFENSTVEPALHDRVPNYYNNSPNKHFEATCSSLLSSEITLISSDHVITKPKAKQGAKKYRTSSEIKDHIMAERKRRQDLTERFIALSATIPGLKKTDKAYILQEAITYMKQLQERVKVLENENKRKTTYSKIFIKKSQVCSREEATSSCETNSNYRSTPPPLPQVEARMLEKEVLIGIHCQKQKDIVLKIMALLQNLHLSLASSSVLPFGTSTVKVTIIAQMGDKYGMTVNDLVKRLRQDLLKSHDIQESHSKECQI

>Glyur000001s00000014.1_GubHLH3-11_Gu

MEEMNTPXNASAASSWLSDFDIDEYNIFPEECHLKFLDADDEVEEFLSRDIASALQGPTLQQQSLTSECTSTTTLSNSFTDETSFDLDQXPTKMPKTISSGSITENFSPKLSPSTSSPISSFHQSQILSFDNPNSSPTQFYGFDRGTLNPPKQNEAVPXLGNTHFSTDQNPKGSSKNQNCETKTSHGTKRSPAHAXDHIMAERKRREKLSQSFIALAALVPGLKKMDKASVLADAIKYVKELKERVVVLEEQSKKTKAESVVVVTKPENCSDDDCSSCDESVEAAGGSESSLFQAEARVSGKEMLIRIHCQKQKGLLVKIMAEIQTLHLFVINSSVLPFGDSILDITIIAQMGEGYNLTIKELIKNLRMATLKSMS

>Glyur000001s00000016.1-11_Gu

MTSMEESWTDWLCDMDPDDYSFINQSGINVGDIGSLASQHDIAAALQEENPQRLSFXTENTSTMSNSSVGTESSXERPASKLLKTQHFSQKKASSPSSGSYILSFDNAINPPPIKVEPALKPETKVLNSKNEPRRATQKSNCKKTESLSXSIYHTPDHIIAERMRREKISQQFIALSALIPNLKKMDKASVLGDAIKYVKELKEQVKVKEEEXSKRKNMESAVPVNKYQPQLSADEDDTSSNSCNNNGNSSDDISKKNLSLPEVEARVSGKNVLIRXLCEKEKAVLVNIFTEIEKLHLLVISCSSLSFGSSVVAITIIAEKMEDDEFNMGVEKLANNIRGMEADPTTLFHQEYHPMDYYAFQLDDIDFKSLSASPDQSYSSHMGFNSEISSTQNSFPVESPDQSVASAARPTKQLKTTYWNAYGTDNSXNHNNNNINPKASSSSSSKIISFENSNTTSSVTSHNSQKFYNNTGAKVVKPKSETTTGYGENLDFSAGFFHGENYDDKSFLNYDKRENKAATNTIRNQTQARDHVIAERKRREKLSQRFIALSAILPGLRKMDKATVLEDAISYLKELQEXVKTLEEQAVDRTVESAVFVKRSVLLADDDGSSCDENSDXSSLPEIEARVSGKDVLIRIHCDKHSGRAATAILNELEKNHLTVQSSTVLPFGNNTLDITIVAQMNKEYCMTIKDLIRSLSQALRQLI

>Glyur000212s00017507.1-11_Gu

MVMEKLNTSTDASATSWLSDLLLEEIEDCNLFQQCHGPNLLGGDEEELLSHDIVSALQQPLSSESYSSXPVSNSETLMNSPTEETSLERPTKQLKTKHTTTLPHELSPSSSSPSPSPSSPCTSQILSFDNSNSPSSANTTHFYGFDNCTLNPKQNEVVPLTPAQQLRNMNFTGQTRKGSSENQNLETVNPQGQGTKRSVAHAQDHIIAERKRREKLSQSLIALAALIPGLKKMDKASVLGDAIKYVKGLQERLKLLEEQNKNGAVESVVVVNKPQLSSDDHSSTSSDDGSEAASGGNNNNNNSEAQPLPHVXARVSEKDVLLRIHCXKQKGLLLKILVEIQNLHLFVVNSSXLPFGDSILDITIVARMDTGYNLTXNDLVRNLRVAASRSMIIDPNSVSQVTDIETAASKNDINGGNLDKLVEPEDYNFINQSNTNEVEGSFPSHRXVVVAPLQQEHNLQQGSFSAGSHXSSSTMSNSSGDVSSYIERPSKTLKTGPSNYSANNTXYLPQKKDSHSSLSYILSFNNESSEPILNIDSTLKPKGKVVNNHGRSLPSKGSLKKEPKSSXQESKKTDSVARSHHHHAQDHIIAERKRREKISQQFIALSALIPDLKKMDKASVLGDAIKHVKQLQEQVKLLEEQAKRKSIESVVYVEKSKSSAAEEDVSDDTSSNSGKGNSSCDPSKTTNLSSQMPEVEARVSEKNVLIRIHCENQKGVLMNXIKEIENLHLSVTNSSTLLFGTSKLDITIIAEMDEEFSLSVKELARNLQGMEDSTLFHQYPMDYSLDDFDFQSTFSVSPETESDDQSFYRYFNFETTPNCFPVSESXDDESVALAPASRPTKKLKSNDWIVPNTNTKPKPSSSPSAQLISFEHASQQFYNLQDSGIKPKVVKKGCNENTGFXALVSQGSYEDKCFSNYDNGANQQVATTTTCRNPTQAQEHMDKASVLGDAIKYVKQXQEKVQTLEEKAAKKKAGSTVLVKRSIIFTDDIDSSSNDSNCEQPLPEIEVRVCGNDVLIKLQCHKHSGPAATVLRELEKLHLTVHSSSFLPFGNNIVDVTIVAQMKKENCMTAKDLLGSLRQALRQFNQFD

>Glyur000270s00013292.1-11_Gu

MEISSIKGLHELGIIEDPTFLHQWHLSXIDTTSLTAAAAFEEALQKHSFSNYYNPNFNXKSCMETSPAAGIERPNKQFKNNSWSPHHGITSETNYASNLLSFSDHSNYTSQQLGQVVKPKVEMVCNPKMDNNTSSITLANMLVSQQGALLGNNQNYVFNKACQLDQDKKIETTHPNKHSQSQAHDHIVAERKRREKLSQRFIALSALVPGLKKMDKASVLGDAIRYLKQMQEKVSALEEEQKRKKTVETVVIVKKTQLSINNDADEDSFSDTAGGTFNETLPEIEARFCERNMDMGFCMTVKDLVRNLRSAFSSFM

>Glyur000319s00018968.1-11_Gu

EMGGDNNNSFGQSHNIMNYSLDEGGKLILTEIEQQQQPSSNFSFEGTYHSSSTMQHNATFNINNVEIGATTTTSTIDNSSSLSNKETNFDQGHDNNNMLIKSNNNKYSKFKPAAALPSHSHQTRYILSFEKSTVELPAVAALHRLASPDQPPPGATAATCTLMNDHVIISDEPANDAKQGSSCCSRRFRSSSETKDRIVAERKRRQVLTERFIALSATIPGLKKSDKAYVLREAINYVKQLQGRVKELENKNKSKXADSAISIKKSQEEYCNTIEEIITSYEDNSNNSTGHGHRLIKKAALYLPQVEARVLEKEILIGIHCEKQKDIVLRIMALLQNLHLSLATSSVLPFGTSTLKVTIIAQIMEVSTSRLVMRLDDSTIQLEEEDDVHNFLQAQ

>Glyur000363s00015360.1-11_Gu

MEISYIRGLPDQEIMEDPFSLHQWHLSSIDESSFLPIAAASGETLQHNSLNYPNFNPRTWVEERTTKHVKNNSWNTSKTAQASETQFVSCPNLLSFVDSNHMNHPKDVEMVCPKINCTIPSDMISQGILENQNYVKTMSSRPKLSQPQDHIIAERKRREKLSQRFIALSALVPGLQKMDKASVLGDAIKYLKQLQEKVRALEEEQNMKKNVESVVVVKKSQLSNDVENSSSESGCPFDVELPEIEARFCDRNVLIRVHCEKSKGVVEKAIHKIEKLHLKVTNSSVMTFGSCALDITIIAQMDMEFCMTVKDFVRKLRSAFTSFM

>Glyur001390s00030766.1-11_Gu

MEHNPWAKWSSSDLEIMDHDVNECLCHTXTFDEEFLRDILQQPQEYSSTTTTAPVATTTNNTHSQLVQQNAAEKTIITSSSSSSPTTFILSFDKSTALLPTHDDEHDIPIPNSSVWKEVSPQLPQLGINHHHPTNSLSLCSSRPNQAGTNKKAXSASETLDHIISERNRRQELTRKFIALSATIPGLKKMDKAHVLXEAINYVKQLQERVKELEEDMKKNGGVESVITIARSHLCIEDDTATEEECYGLNEALPEVEARVLGKQVLIKIHCGXQKGILLNIMSQLERLHLSITTTNVLPFGNTLDITIIAQ

>Glyur001390s00030769.1-11_Gu

DMNGDNSFNQYQTNSLVNIEEHGRTETTHPLYFSSSNVVCVPSLKQGYNLEPLIGAQYSSSSSSCSYHHXYVSLEEEKRSFEVPINMRATTSRGTKRGRSSWETQEDHIMSERKRRQEMAERFVALSAIIPGLKKIDKASVLREAINYVKQLQERIAVLEQETSKRKSTTSIISIRKSHSQSHXSRETNSNVNYLGSNRVFPEIEAIXIETEKELLIRIHCEKQNGILLKLLALLENMHLSIASSSVLPFGENTLNITIIAQLRAKD

>Glyur001827s00038939.1-11_Gu

MEEPCQNWFSDMEIQDDVLLINQHHKIDHSLIDEDHIFREIIMHQPSSAFSNSDDTELSHSPTXQKCGDHSNNNNLMINNSNSSNINSFNMFSPPSQHAPQNIKPATATATSPTSFILSFGDSTVVAATRETCGGKRPFHDKVIGGGGGTTPPPPPPPSKRVAEKHDLLRENQSVAKRSRSSTETLDHIMAERKRRRELTERFIALSATIPGLKKLEEQSKKACIESVSFIRKSNLTTTEKGTPSSAMNSDDCHRTNETLPTVEAR

>Glyur002002s00040221.1-11_Gu

MEESGENWPSDSDLEMGDDHVKDFIFEDEEFGGGGGEEGHSSVDPMMGETERIKGRQQELTERLLALSATIPGLKKMDDTSILDKASNYVKQLQERVRELEQEVEFHICSNNNNNNKGTTTSNCEVNSEVKARVLQKEVLIIIHCESQKGIMLKILTQLENLHLSVVNNSVLRFGKSTLDITIIAQMGDGYNMTVDELVKTLRIVILTQ

>Lj0g3v0034169.1_LjbHLH001-12_Lj

MMQISSTNYLPELGMEEPTYFHQFPMDSSYAEFQDLEFESFTASPDESYSSGNKRFNSESPDYSFAPARPTKQPKTETTWSAYGTDLIASKASSSSSSPKIISFEHSNVSSVTSSQPFHNMGAANVVKPKMESGFGENLDFAAVISQGAYDDKSFLYNENKLAATATIRNQVQARDHVLAERKRREKLSQRFIALSAVLPGLKKMDKATVLEDAISYLKQLKEQVKTLEEQVADKTVETAIFLKRSVVFGDDDGSFSDENSDQSLPEIEARVSGKDILIRIHCEKHHGKAVSAILVELEKHHLTVQSTSILPFGNNTLDITIVTQMKKEYNLTTKELIKSLSQSLKQCA

>Lj0g3v0069559.1_LjbHLH004-12_Lj

MRNSSSARSPHQAQDHIVAERKRREKISQQFIALSALIPGLKKIDKASVLGDTIQHIKQLQEQVKLLEEQSKRINAESVVYVDKSKSEDVSDTSSNNVSLPEIEARVLENKVLIRIHCEKQEGVLMNILREIDNLDLTVINSSTLHFGASKLDITIIAEMDDEFKLSVKELARKLRVGLLQLM

>Lj0g3v0140069.1_LjbHLH014-12_Lj

MEEPTYFHQFPMDSSYAEFQDLEFESFTASPDESYSSGNKRFNSESPDYSFAPARPTKQPKTETTWSAYGTDLIASKASSSSSSPKIISFEHSNVSSVTSSQPFHNMGAANVVKPKMESGFGENLDFAAVISQGAYDDKSFLYNENKLAATATIRNQVQARDHVLAERKRREKLSQRFIALSAVLPGLKKMDKATVLEDAISYLKQLKEQVKTLEEQVADKTVETAIFLKRSVVFGDDDGSFSDENSDQSLPEIEARVSGKDILIRIHCEKHHGKAVSAILVELEKHHLTVQSTSILPFGNNTLDITIVTQVNILINLVIFPCYRTIIYSIKIGIIGKFK

>Lj0g3v0151119.1_LjbHLH016-12_Lj

MMLVVDDKEFLSNDDIASVLQKQTLQQSLSSDCTSITLSNSFTDETSFDFADTPSITENFSPELSPSSSISSFKSQILFLDNTNSSPATNTTTQISGFDSSTLNPKQNETVLVPLPQLGNTHISTQNSKGSSKNQNFETKSSHGKRSPAHHHDHIMAERKRREKLSQSFIALAALVPGLKKMDKSSVLTDAIKYVKELKERLAVLEEQSKKTKVESVVVPINKPAPELCGGDNDDESLVQVESRVSEQDMLIRILCKKQKRLLTKILAEIQSFQLSVVNSSILPFGDSMDITIIAQMGEGYDLTAKELVKKLRVAILKFMS

>Lj0g3v0186939.1_LjbHLH021-12_Lj

MVESGENWPCDHSVLEVGGGGDDDDVKDLCFEDEFIGDDNDDGHSFAEETVDPLTDRKERQRELSEKLLALSATIPSMKKMDNTSILDKASNYVKQLQERVRELEQEVDQSHIRSNKGTTSCEVNSENYRTGGRTNENFPEVRVRVLQKEVMVIIHCEKQKGIMLKILSHLENLHLSVANTSVLQFGKSTLDITIIAQMGDEYNMTVDELVKTLRVAILTQ

>Lj0g3v0292969.1_LjbHLH032-12_Lj

MEISSITGLPDQEMMGDPTLLHQWHSDDSSLLPMAAAFGETLQHHSFTYPNFNLITPMNQFQLGSNHMSQFQLGLQKLKDEMGCPTINITTPLDMISQGNLEDQNYVFKTCQEDHKKILTRPKLSQPQDHIIAERKRREKLSQQFIALSALVPGLQKMDKASVLGDTIKYLKQLQEKVRALEEEQNLKKNVQSVVIVKKSQLGSFDKELPEIEARFCERNVLIRVHCEKSKGVVEKTIHEIEKLHLKVINSTALTFGSCALDITIIAQMDVGFCMTVKDLVRKLRSAFTSFM

>Lj0g3v0312929.1_LjbHLH036-12_Lj

MEESWENWHLHPEMDCDDDYFANKSNMEEDEEFLRQILLQAPAFSSESESNNSLHASSTVASEEIIGNMVKSHSSNSIVSSQQGPKKPRGSSSPKAYILSFDNSTIIPANPEGKLLGSNKGSTRGTKRCSDSSSALPNKLQQVQNTESEKTRANNQGTKRARTTSQTIDHILAERKRRQELTERFIALSATIPGLSKTDKASVLRAAIDYVKQLKERVQELEKQEKKNGVEKVIVIKKPDLRGNEDTTNSGDIITTSSDHDDCSTILPEIAARALGKEVLIEIHCEKENGTELKILDHLENLHLSVNGSSVLPFGNSALSVTVIAQMGDAYGMTMNDLVNSLRQLLLKSQMSSDSDPY

>Lj1g3v2883900.1_LjbHLH054-12_Lj

MAHLLENLVSHMEMDDDIFNQSDNSFDEQGFLREILQLPDFSPQSDETSSPCPYFKIQNNISVDGVTSPPNSISTLSLEETGFERNDPLHKSHSSNSIMSLENNLNSPTTYLLSFDKSSVEQITHESSPDHNSVLRSNNRTRGSELTPGMHQATKKVRRSGTPHHIMAERKRRKELTQNIIALSATIPGLKKTDKVYVLREAINYTKQLQERVKELENQNRDKNVDSATFTMKSQDSSNKSTTYCETNRESLPIGVKARVLEKEVNVGIHCEKQKDIVHNIYVLLEKIHLSVTCSSVLPFGTSLIINIIAQMDDEYNMSIDELVKNLKEYMLEACGVQ

>Lj2g3v1984450.1_LjbHLH081-12_Lj

MDCFACPYDDFDMHSFSTLPDSDSSYQCFNYETTPNFVPAEVSNYSVASAKPTSDMIMPSSSPIPSQLISFNHFNNPALVSQGASEDSIFSNYGNPPNQFAARTPTQTQEHIIAERKRREKLSQNFIALSAILPGLARMDRGSILGVAIMYVKQLQEQVQTLEEQAAKEGVGSALLVKRSVLFISDDSLTKSDKIFDNHCYKSLPEIRVRVSGNDVLIKIHCHKQSGYETIILGEIEKHDHLTVHSFSFLPFGNTIIDATIIAKMNKENCKTSKDIVRSLQQALKQII

>Lj2g3v1984550.1_LjbHLH082-12_Lj

MMSPEGESWTSWLCDLEPEDCNFLNESDTNWAGEISFPSEAQEQNLQRSFTIDSNHSHSTTTSTFKTEHCSSTMSNSSSGEDDNTTSERPSKTLKMSNASPSYILSFSNENPAHILNFDSTLKPKSKALTVNVNHHGNRSLESQKKETKRKVQEGEKARSPHQAQDHIIAERKRREKISQQFIALSALIPGLKKMDKASVLGDAIQHVKQLQEQVKLLEEQSQRKSSESVVYVEKSKSSVVDDDVSLSDTSSNSDNSNNFSLPEVEARVSENKVLIRIHCEKQEGVLMNILKEIHNLNLSVINCSTLLFGTSKLDITIIAEMDDGFRLSVKELARKLRVGLLQFM

>Lj2g3v1984620.1_LjbHLH083-12_Lj

MEKLNTPTEASSSCWLSDLLLQEMDDCNFFQQCNPNLLGDEEFLSHDIVSAFQSDNLQQQQQPLSSESYSSYPVSNSETVWNSSTDETCFERPAKQQKTKHVLPDQSNSSSPSSPTSQILSFENTHLYGLDCSLNPNLQNEGVSVSTPQLRNVNFPAQNRKGSTQNQNFETITNPQGKGSKKSHGQDHIIAERRRREKLSQSLIALAALIPGLKKMDKASVLGDAIKYVKVLKERLRLLEEQNKNRAMESVVVVNKPQISNDDNSSSSCDDGTIIGSEEALPHVEARVSEKDVLLRLHCKKQKGLLLKILFEIQNLHLFVVNSSVLPFGDSILDITIVAQMGAEYNLTINELVKNLRVAALRSMSS

>Lj3g3v0028570.1_LjbHLH086-12_Lj

MESPWKNCLPNVEMEDGDSFNINNEEHLFLEEILQQFALSSETGSLANNLLNSTDGGSKGVAIDESQPAPETHDDQAGGRRERRKGKSSSGHLDHAVAERKRRLELTQGFIALSALIPGLKKIDKVSILKEATTYVKQLQNRVKEMEEQSAKKIIVESMSVLNDAGFHYDQGIVPYNMNSDNRHKLNDAALPEVEARVMEKQVLIRIHCDGNQDILLKTLSHVTSLDLSIVSHTMLPFRKISLDVTIIAQMGDNYKITLKDLVRSLRKVLLESTDMK

>Lj3g3v0028580.1_LjbHLH087-12_Lj

MMEQSWENFPLHQMEILGSSDEDEDEFLRDILLPPDLSPDHESQSLVVQKKLQPRTFILSFDNSTILPASATSERGTHSNSKSSNNSSLSSRRKRNLEKNHVSLEPKLPSTTTQRGKKNRSDSQTTDHVMAERKRRQDLTEKFIALSATIPGLKKTDKTSIVHGAIDYVKQLKERVTELEQQGNGRGKESMIVLKKMNLSANREDTPTTSSETNNSEDLCRPRELFPDVKARVLENEVLIVIHCERENGIELKILDLLENLRLCVTGSSVLPFGNSTLSITIIAQIGDACENTTVPDIVDNVRQALLNDVTTDRFRPQLSY

>Lj3g3v0030630.1_LjbHLH088-12_Lj

MEAQRGDIIMRQHPETLSSCRTQTQTHHHSMYSSNCLDNLGFKGQNSGPHLGTKRIRSSSEIQDHVMAERKRRQEMAERFIALSAMIPGLKKIDKASVLGEAINYVKQLQERIAVLEEEGNKKKKSTTNSLILIKKSQSYPHNSDDSDQVLPEVEAIGLDSESEKELLIRIHCEKRKGILLKLLALLENVHLSVASSNVLPFGKNTLNITIIAQMGEEYKLTGEELVKVLREGLLK

>Lj6g3v2171830.1_LjbHLH152-12_Lj

MHMEISSLPEQGLIEDPNFLHQWRFGSIDPPSLTASAAFGEALQKHSFTNNSPNFNHKTSTDTQYASCQNLISFADSSHHMNQLGLVRPKEEMESPKIDNTALANMLISQGAFMNQNYAFKACQEDKKIGACHKLSQPHHEHVIAERKRREKLSQRFIALSALVPGLKKMDKASVLGEAIKYLKQMQEKVSALEEEQKRKKTVESVVILKKSLCNDAEDTSSDNGGEFDDTLPEIEARFCERNVLIRLHCLKNKGIIEKTISEIEKFHLKVINSSALTFGSFALDITIIAQMDMGFCMTGKDLVRNLRSALSSLM

>Medtr0011s0210.1_MtbHLH004-13_Mt

MEEINSTAMNVSSSSSWLSDLEMDEYNLFAKECNLNFLDTSVEDFLSHDITINVFQEQNKQQCLTSGSTSTTTLSNTFSDETKLDCFDFNIDKTIMEMKTIDHSDKINETFTQKRSSSFQVQIPSFDSPPNSPTTSSQQYPTLNSIQNERVSVSPTELENKNHSTKTSKTSKTKRSRANNGEDHIMAERKRREKLTQSFIALAALVPNLKKMDKFSVLVDTIKYMKELKKRLEVLEEQNKRKKTESHVILTKPDLCSEDDSSSFDEHNESVVGSIFQVEAKVLGKYMLIRIQCKEYKGLLVKIMVEIQRFQLCIVNSSVLPFGDSIFDITIIAQLGEGYNLSIKELVKNIRKEALKFMSSR

>Medtr0011s0260.1_MtbHLH005-13_Mt

MEEINSTAMNVSSSSSWLSDLEMDEYNLFAKECNLNFLDTSVEDFLSHDITINVFQEQNKQQCLTSGSTSTTTLSNTFSDETKLDCFDFNIDKTIMEMKTIDHSDKINETFTQKRSSSFQVQIPSFDSPPNSPTTSSQQYPTLNSIQNERVSVSPTELENKNHSTKTSKTSKTKRSRANNGEDHIMAERKRREKLTQSFIALAALVPNLKKMDKFSVLVDTIKYMKELKKRLEVLEEQNKRTKTESHVVLTKPDLCSEDDSSSFDERNESVVGSIFQVEAKVLGKYMLIRIQCKEYKGLLVKIMVEIQRFQLYVVNSSVLPFGDSIIDITIIAQLGEGYNLSIKQLVKNIRKEALKFMSL

>Medtr0246s0020.1_MtbHLH007-13_Mt

MEEINNTPMNVSEETSKWLSDLEMDEYNLFPEECNLNFLDADEEEFLPQEQTQQQCLSSESNSTTFTNSFTDETNFDSFDFDFEIERPTMELNTIFSDNSIIETISPKLSPSSSNSSLHSQILSFDNLPNSPATNTPQFCGLTPTLISKSKQNKTVLVSPPQIRNIHVSTQNPIGLSKNQNFATKTSQTKRSRANADDHIMAERKRREKLSQSFIALAALVPNLKKMDKASVLAESIIYVKELKERLEVLEEQNKKTKVESVVVLKKPDHSIDDDDDDDDNSSCDESIEGATDSSVQVQARVSGKEMLIRIHCEKHKGILVKVMAEIQSFQLFAVNSSVLPFGDSIDITIIAEMGERYNLSIKELVKNLHMAALKFMSSKITD

>Medtr0246s0050.1_MtbHLH008-13_Mt

EKVGEYIFDEECYLNLLDADVVEEFLSRDMASVIAFEEQRETLQQCLTTECISTTLSETFNGETSFESFDFDFENPTKKLKFFDRSDNITKNFSQQLSASPSTFQSSQIPSLPNLGNTHFSALQTSKESSKNQNVETKTSQSKRSSAHVKDHIMVERKRREKLGQAFIALATLIPDLKKKDKASVLADTIKHIKELKERLAILEEVGKNTKEDQSMMVCNKPDHCCETESVGDGTAIKVAAKVSGKKMLIRIHCQKHDGLLVKVITEIQSFQLLVVNNRILAFGDSFHDITVIAEIGEGYNLTIKELVRNLRMAALKFMSS

>Medtr0250s0040.1_MtbHLH009-13_Mt

MNEYNLIAEECNPNFLIDVEELLSHDIANEINETFSPKLSSDSSFQFQMPCFDNNPNSSTTEMENMNHSTQTSDESSRKRSRGNHGHDHIMAERKRREKLTQNFIALAALVPNLKKVDKYSVLVDAIKYLKELKKRLEELEEQNEKTKIESQVIVTKPGIYSDDNSSTCDESIHSVVGLPFQVEARVLGKYILIRIQCQEHKGLIVKIMVEIQRFQLFVVNGSVLPFGDSLIDVTIIAQMDEGYNMSIKELVKNLRMAVLKFMST

>Medtr2g010450.1_MtbHLH043-13_Mt

MSSHMEISSIRGLPEMGIMEDPNFLHHFNNHLSSIDTNNLTASAFGDALQKHILSNNPNFNNKTCMETSPTGNERPAKQLRNNSWNYNNSPPTSDTQYDNCCSNNLLSFADLNYTNQLGLLKPKSEMVCPKIDNTSTLANMLITQGNLFGNQNHVFKAVQEAKDIENRPNKLSQAHDHIVTERKRREKLSQRFIALSALVPNLKKMDKASVLGEAIRYLKQMEEKVSVLEEEQKRKKTVESVVIVKKSQLSMNEAEDRADTNNSTYDETLPEIEARFCERSVLIRLHCLKSQGVIEKIMSEIEKLHLKVINSSSLTFGNFTLDITIIAQMDVGFCMTVKDLVRKIRSAYSSFM

>Medtr2g104490.1_MtbHLH065-13_Mt

MEESWEHWPLHSELGDVEVLLGQYCHQTSADDEEEFLRDIILQQPVTYSGSISSSNEMDGSDCSKKKLHPSSSPFTPRTFILSFDKSTIIPATTTPESEEVSIPKSRSNNNKRSLEPKAKASNQTGKKSRSGSQYLDHIMAERKRRLELSQKFIALSATIPGLKKMDKNYILGEAISYVKLLQERVKELEDQNKNSKESTIILKKTDMCVSEDTTSNSDQDCCKSPLFDVKARIMENEVLIQMHCEKENDIEIKIYNVLENLDLFVTASSVLAFGTSTLGFTIVAQMGEGYKTTVNDLVKTLWQVLNSKTDPF

>Medtr2g104500.1_MtbHLH066-13_Mt

MGVEYLLEMCHEEKEFIRDFMAQPVAGAAVAATESSSEMIVVSDESPELLPPSTSSRACIISLDNSAAIPLPAVMSKSKPPRCPTKKRTSERGKGEKKNIKTLDHAMGERKRRLELAHKFIQLSTIIPRSNKTNKASIVAGATNYVEQLQKRVKELEAQQNKRGKEPMILFNKENSCEMNLDNCFRPNELLPDVKVKVSENNILIYINCEKENGIQHKILDMLQNLHLFVTSTSVLPFGNSTLAITIIAQMGDAYKVTQMDLVDNIRQFMLKDAT

>Medtr2g104530.1_MtbHLH067-13_Mt

MDMDWLESMCHQDGEFLGEYMAQQSSIVAAGAAAATEINASPPELLPITSILSSNNSMATILGKSKPPCYPKKRTSSNLNFESKANGTGLAKEEKIIRSKSKTLFHTLAERRRRLELAHKFTELSAIIPRSKKTDKASIVQGAINYVEKLQKRVMELEVQQNKRGKEPIILLNKENSCEMNLDNYLRPINNFLPDVKVKVLENNILIYINCEKENGIQHKILDMLQNLHLFVTSTSILPFGNSTLGITIIAQMGDAYRMTMMDLVDNIRKLLVNGANRRT

>Medtr2g104550.1_MtbHLH068-13_Mt

MEQTWENWPLHSEMVDVENMQGQYCHQTSTDDEEEFLRDIILQQPVTYSGSSLSSSSEMDGSDSSGKKLHLLSTPSTPRTFILSFDKSTIIPATTTPESEEVPRTKSRSNNNKRSLEPKAKASNQTGKKSRSGSQCLDHIMAERKRRLELSQKFIALSATIPGLKKMDKTSILGEAINYVKILQERVKELEERNKRNNESTIIHKSDLCSNEHNNTSNDTNSDQDCCKSSLPDVKARVLENEVLIEIHCEKENGIEIKILNLLENLHLIVTASSVFPFGNSTLGFTIVAQMGDEYKMKVNDLVKTLQQVLLNMRS

>Medtr2g104560.1_MtbHLH069-13_Mt

MENWFSNMAMKGTDNSFNEKHPTLIGHCSNSVPHAANATHGKNKRVRSSWEIQGHIMSERKRRQEMAERFIQLSAMIPGLKKIDKVSVLGEAINYVKELKERISMLEQQYYERNKSTKSIISIRKFQSHPLNDNLDSNHVLPEVEAIGIESEKELLLIKINCEKREGILFKLLSMLENMHLYVSTSSVLPFGKNTLNITIIAKMGEEYRITIEELMTKLKQDLLKLYDM

>Medtr2g104590.1_MtbHLH070-13_Mt

EMEECLSHTNNIFDEEFLKDILNQTPQDQSINVSHSTQHAHHDSLPLSSSKADHGSNSRKTRSASETLDHIITERNRRRELTRKFIELSAFIPGLKKTDKVHVLGEAVKYVAQLQERVKELEEDIKKKGAGSLITITRSHLLDDNDTAMGEMNTKECYRHNETFPELEVRVLGKELQILHLSITTTNVLPFGNTLNITIIAQMGDKYKLTVEDLVKKLRVVATLQACDDVQQ

>Medtr2g104650.1_MtbHLH071_TSAR3-13_Mt

MEEHLCHTNNTFDEEFLRDILYQIPQDQFNVPIATTDLVNNSSINVSQHAEEMPTNSLSIPTTEQHHDSLPLSSSTANQGSNSKKPRNTSDTLDHIMSERNRRQLLTSKIIELSALIPGLKKIDKVHVVTEAINYMKQLEERLKELEEDIKKKDAGSLSTITRSRVLIDKDIAIGEMNTEECYGRNESLLEVEARILEKEVLIKIYCGMQEGIVVNIMSQLQLLHLSITSINVLPFGNTLDITIIAKMGDKYNLTIKDLVKKLRVVATLQVSHNVQFHI

>Medtr4g009110.1_MtbHLH087-13_Mt

MEESWENFHLDMELDCGDDYFIDDCNNLNIDGDDFIREILLQTPEGLISSESDHSFFHVQTDTVTVNVNGGVEVVGNTVKSKSSNSIVSQQHQPQEQEQQHGLKSKKVPRKSSSPKTYILSFDNSTMIPATPEPCVNLSSRNKRSRESTQKAEVKTNQQINGVKKGRSSSQCIDHIMAERKRRQELSEKFIALSATIPGLSKMDKASLLREAIDYVKQLKEHVEELEKQDKNVGVTPVMVLRQPYSCGINEYTNSGETSCGDDCNHHILPDIEARVIGKEVLIEIHCEKQNGIELKLLNHIENLQLFVTGSSVLPFGKSAISITIIARMGDECIVTMNDLVKSIRQVLLKP

>Medtr4g009540.1_MtbHLH088-13_Mt

MEESWENFHLDTELDCGDDYFIGDDFLQDILLQTPPQSLMYSESENNSVTVNVNGGVEFVGNMIKSNSSNSIVSQQHQPQEQEQEHGLKSKKVPRRSSSPKTYILSFDNSTMIPATPNYKNKRSHESNQKSEMKINQQNGVKRGRSSSQCIDHIMAERKRRQELSEKFIALSATIPGLSKTDKASILREAIDYVKQLKERVDELEKQDKNVGVTPVMVLRKPYSCGNNNYNEDTNSSETSCDGDCKNNILPEIEAKVIGKEVLIEIHCEKQNGIELKLFNHIENLQLFVTGSSVLPFGKSAISITIIAQMGGGYKVTVNDLVKSIRKVLLK

>Medtr4g066380.1_MtbHLH092-13_Mt

MEEINNPAMKVSSISSWLTDLEMDEYNIFAEECNLNFLDADVGGFLSQDISNVFQEQNKQQCLSLGSTSTNDLSKSFIHETIDNSDKINKSLSPNLSPSFQFPIPKENETISMSPTELENMNHSTETSKGSLENENLETKTSKSKRPRAHGRDHIMAERNRREKLTQTFIALAALVPNLKKMDKLSVLVDTIKYMKELKNRLEVVEEQNKKKNKSSTKPCLCSDEDSSSCDDSVECVVGSPFQVEARVLGKQMLIRIQCQEHKGLLVKIMVEIQRYQLFVVNSSVLPFGESTLDITIIAQLGEGYNLSTKELVKNVRMAVLKFLGEAYVTPS

>Medtr4g066460.1_MtbHLH093_TSAR2-13_Mt

MEEINNSAMKVSSSISSWLSDLEMDEYNIFAEECNLNFLDADVGGFLSNDISNVFQEQNKQQCLSLGSTFHETIDNSDKNNESLSPSFQFQVPSFDNPPNSSPTNSKENIETIPLSPTDLENMNHSTETSKGSLENKKLETKTSKSKRPRAHGRDHIMAERNRREKLTQSFIALAALVPNLKKMDKLSVLIDTIKYMKELKNRLEDVEEQNKKTKKKSSTKPCLCSDEDSSSCEDNIECVVGSPFQVEARVLGKQVLIRIQCKEHKGLLVKIMVEIQKFQLFVVNNSVLPFGDSTLDITIIAQLGEGYNLSIKELVKNVRMALLKFTSS

>Medtr4g067010.1_MtbHLH094-13_Mt

MEEINNSAMKVSSSISSWLSDLEMDEYNIFAEECNLNFLDADVGGFLSQDISNVFQEQNKQQCLSLGSTSTNDLSKSFIHETIDDSDKISESLSPNISPSFQFQIPNSPPSNSKENETISVSPTELENMNHSTETSKGKRPRAHGRDHIMAERNRREKLTQTFIALAALVPNLKKMDKLSILFDTIKYMKELKNRLEVVEEQNKKTKESSSTKPCLCSNEDSSSCDDSVECVIGSTFQVEARVLGKQMLIRIQCQEHRGLLVKIMVEIQKYQLFVVNSSVIPFGDSILDITIIAQLGEGYNLSIKELVKNVRMALLKFIHHNMNMSTCN

>Medtr4g092700.1_MtbHLH107-13_Mt

MMDLIDSLGIFTLTSFDFKVSFLMENISFIRGFPDQEMMEDPLLLHHQWHLSSINESNSLPIGSAFGDTSQHHSYVYPNFNPRTSMETAQTLETQFVSYPNLLSFVDLNQLNQLGLVKPKDEMIGSQNNNATSSDMISQGTFETKKVATRPKLSLPQDHIIAERKRREKLSQRFIALSALVPGLQKMDKVTVLGDAIKYLKKLQEKVKVLEEEQNMKKNVEFVVVVKKYQLSNDVENSSAESGDPFDEELPEIEARFCDRNVLIRVHCEKIKGVVEKTIHKIEKLNLKVTNSSFMTFGSCALDITIIAQMDVEFCMTVKDLVRNLRSVFTSFI

>Medtr4g097920.1_MtbHLH110-13_Mt

MIQISSTNYMPEFGLMEDTTLFSDHEYQMDSYAFQFDDMAYFKSFSESPQESTYSSHTNINNKRIHSESTQNSSFPTQSPDQSVASATPPTKLLKASPKIISFDYSNNDSKVKKPKTEIGYGENLNFGSVISQGDYYKRENKVSAVNRNPIQAQDHVMAERRRREKLSQRFISLSSLLPGLKKMDKATILEDAIKHLKQLNERVKTLEEHVADKKVESAVFMKRSILFEEDDRSSCDENSDQSLSKIEARVSGKDMLIRIHGDKHCGRTATAILNELEKHHLSVQSSSILPFGNNYLDITIVAQMNKEYCLTMKDLIRSISQVLRQLI

>Medtr4g097940.1_MtbHLH111-13_Mt

MIQISSTNYMPEFGLMEDTTLFSDHEYQMDSYAFQFDDMAYFKSFSESPQESTYSSHTNINNKRIHSESTQNSSFPTQSPDQSVASATPPTKLLKASPKIISFDYSNNDSKVKKPKTEIGYGENLNFGSVISQGDYYKRENKVSAVNRNPMQARDHVIAERKRREKLSQRFIALSSILPGLKKMDKATILEDAIKHMKQLQERVKTLEEQVADKKVESAVFVKRSILFDNNDSSSCDENSDQSLPKIEARVSGKDMLIRIHGDKHCGRSAAAILNLLEKHHLTVQSSSILPFGNNYVDITIVAQMNKEYCLTIKDLVRSINQVLRQLI

>Medtr4g097950.1_MtbHLH112-13_Mt

MKTEHLSQKTASYVFSFENENPPPRKVEPALKPKTKVVNSKNGPRRVKNNESNKKNGSFSKSTTTHHTPDHIIAERIRREKISQLFIALSALIPNLKKMDKASVLGDAIKYVKELKEQVKMLEEQSKSVEPVVVVKKLSELSSDEDVSDTSSNSCNGNSDETSKTNLSLPEVEASLSGKNVLIRILCEKDKAVMVNVYREIEKLHLLVINASSFSFGSSALAITIIAQMENELNMSIQQLAKKLRVGLVQLN

>Medtr4g098035.1_MtbHLH113-13_Mt

MEQINNTPMNISADSSLLSDLEKVGEYIFDEECYLNLLDADVVEEFLSRDMASVIAFEEQRETLQQCLTTECISTTLSETFNGETSFESFDFDFENPTKKLKFFDRSDNITKNFSQQLSASPSTFQSSQIPSLPNLGNTHFSALQTSKESSKNQNVETKTSQSKRSSAHVKDHIMVERKRREKLGQAFIALATLIPDLKKKDKASVLADTIKHIKELKERLAILEEVGKNTKEDQSMMVCNKPDHCCETESVGDGTAIKVAAKVSGKKMLIRIHCQKHDGLLVKVITEIQSFQLLVVNNRILAFGDSFHDITVIAEIGEGYNLTIKELVRNLRMAALKFMSS

>Medtr5g014520.1_MtbHLH120-13_Mt

MEKLNSSSNSSSTSWLSDLLLEEMEIEGCDLFQQCEQNLFGEKEFLSNDIASIFQQEENFQHQPLLSSESYSSSYPLEKNFERPNKKLKKNTSSPSSTTSQILSFDCTLNTKKNKVVPLSQTELPQNRKGSLQKQNIVETIKPQGQGTKRSVAHNQDHIIAERKRREKLSQCLIALAALIPGLKKMDKASVLGDAIKYVKELQERLRVLEEQNKNSHVQSVVTVDEQQLSYDSSNSDDSEVASGNNETLPHVEAKVLDKDVLIRIHCQKQKGLLLKILVEIQKLHLFVVNNSVLPFGDSILDITIVAQMGIGYNLTRNDLVKKLRVAALRAMS

>Medtr5g014560.1_MtbHLH121-13_Mt

MEKLNTSSNASSTSWLSHFLFEENEIEGCDLFQQCEQNLFGEEELISHDIASIFQQEENLQQQPLLLFESYSSCTLETNLERDNKKLKTNNTLHEVVPVSQTQLPQNQNIVETKNTQGQGTKRSVAHDHQDRIMAERKRREKLSQCLITLAALIPGLKKMDKASVIGDAIKHVKELQERLRVLEEQNKNSPIEFVVTLNKPKLNYESWSDDGSKAASANNETLPHVEAKILGKDVLIRIQCQKQKSFLLNILVEIQQLHLFVVNNNVLAVGDSIHDITIIAQMGTGYNLTKNDLVKSVQAAVLRSMS

>Medtr5g014600.1_MtbHLH122-13_Mt

MTSNHESWTSWLCDLEPEDYNIINEINIATLPQENNNLQQSFSGGSHCSHTTSNTMSNSSGDVNKTNPSNIGYPSLPKKDSSHSYILCFGNENPESMLNIGSTLKPKGKVSNHGKSLASKGSLENQKKGPKRNIQESKKTDSAARNAQDHIIAERKRREKISQKFIALSALLPDLKKMDKASVLGDAINHVKQLQEKVKLLEEKNQKNNVESVSMVYVEKTKSYSSDEDVSETSSNSGYGNCCHTHTSKPSRSLPEVEARVSEKNVLIRVHCEKHKGALMNIIQEIENLHLSVTSSSALLFGTTKLDITIIAEMDEKFSLSVQELARKLRVVLL

>Medtr5g014640.1_MtbHLH123-13_Mt

MMQIMSPIYVPELGMEDLNFFNQYTMDSLASPTLFDNFGFHFDDNETTPNCFPVETHPDDQTRPTKKIKTSITPSSQSSPQLISFEHSSSTPIASKQFYNLDYSDVKPKVGKRCNENKDFLPALVSQGSYEDQKIFSNYDNQANQTRNTAQAREHVMAERKRREKLTRSFIALSAIVPGLKKMDKASVLGDATKYMKQLQARLQTLEEQAEDNKKAGSTVQVKRSIIFTNNNDDDSNSNNQPLPEIEVRVSSKDVLIKIQCDKHSGRAATVLGQLENLNLTVHSSTFLPFGNNIVDVTIVAQMNKENCVTAKDLLGSIRQALIIQN

>Medtr6g047550.1_MtbHLH138-13_Mt

MEEINNTTMNVSSTNGWLSDLEMDEYNLFAEESNLNFLETDVVEFLSHDIGNVFLEQNKQQCFTSGSTPTSLCNSSSYETNLDSFDFDFDKPNMELKTIDHIHSNKINETFSPKLSPSNSSIQFQIPSFDNTPNSPTTNSSQLCGLDPTFNSKQNSEIKTSKSKRSRTLHGQDHIMAERKRREKLTQNFIALAALVPNLKKVDKYSVLVDTIKYLKELKKRLKVLEEQNEKTKIESLVVVLTKQAFATMTTPPHVMRVLIVLLIYHFKWKQES

>Medtr6g047570.1_MtbHLH139-13_Mt

MEEINNTTMNVSSTNSWLSDLEMDEYNLFAEESNLNFLDTDVVEFLSHDIGNVFQEQNKQQCFTSGSTPKSLSNSSCYETNMELKTIDHSNKVNETFSPKLSPSNSSLQFQIPSFDNSPNSPTTKSSQLCGFDPTLNSKQNLETKTSKSKRSRTLHGQDHIMAERKRREKLTQNFIALAALVPNLKKVDKYSVLVDTIKYLKELKKRLIILEEQNEKTKIESPIILTKPGLCNNDNSSSCDESIDCVVDLPFQVEARILGKYMLIRIQCQEHIGLLVKIMVEIQRFKLFVVNNSVLPFGDSILDITIIAQLDEGYNMCIKELVKNLRMEALKFMSS

>Medtr7g080780.1_MtbHLH150_TSAR1-13_Mt

MEDSLENLISYMEMEDDVILNQSSTTTFDEQEFLKDIILEEPECIELSSYLCSNKTKDNSTTIINVEGDATSPTNSILSFDETSLFCGDYENVETNHKSNNSNSIKSLERSCVSSPATYLLSFGNSSIEPIIEPMSHKTKRRTDESRGVKEATKKVRRSCETVQDHLMAERKRRRELTENIIALSAMIPGLKKMDKCYVLSEAVNYTKQLQKRIKELENQNKDSKPNPAIFKWKSQVSSNKKKSSESLLEVEARVKEKEVLIRIHCEKQKDIVLKIHELLEKFNITITSSSMLPFGDSILVINICAQMDEEDSMTMDDLVENLRKYLLETHESYL

>Medtr8g009010.1_MtbHLH162-13_Mt

MGYDKSHFYLDEDKFQREIVQHQPSFSFESGNSQSSTIQYMNNIVDNVCVTNTVHENIAQEYTWATNSQQLPPSQYILSFENSTMQPSPNSDIATCSSIMVQETTTLNNNVSSELPKIIKKRTKNLRSSCEMQDHIMAERKRRQVLTERFIALSATIPGLKKTGKVYILQEAINYVKQLQERVKKLEKEVPRVEARVIDKEILIGIHCEKQKDIVVRLMALLQNLHLSVASRSSVLPFGSSTLKVTIIAQMDDEFCMSINDLIKVLRNISWSHVRIKRIFLNQLLFMSALKLVENFVWWVFVCLQRKILPKKQSKGEIIL

>Medtr8g009020.1_MtbHLH163-13_Mt

MGGDKSPSLDEDKFQLKNMQQSCFSFEGDNSISQSSTIQYINNNIFDNVCVIPTIQENILKSSNSSNSLISQEYNCPTNSQQIAPSQYILSFENSIMKPSPNSATFSSIMVPKTTLNNNIVSELPKTIKKRTKNLRSSSEIQDHIMAERKRRQVLSERFIALSATIPGLKKTDKAYILEEAINYVKQLQERVNELENHTKRKRDSIIFIKKSQPCIVDKEKSTSCEENSDNDDHRYYSKKEVPRVEARVIDKEILIGIHCEKQKNIVVRLMALLQNLHLSLASSSVLPFGSSTLKVTIIAQMDDEYCMSMNDLVNNLRQNLMESRDNNDNIS

>Medtr8g069740.1_MtbHLH177-13_Mt

MEECLCHTNNTFDEEFLRDILYQTPQDQFSVPIATTGLFNNSSTNVSHSTQHAEEKQANSLPMPTTSILSLDKSVTLLPPTEQHHDSLPLSSSMASQGSNSKKPRSASETLDHIMSERNRRQLLTRKIIELSAFIPGLKKIDKVHVVTEAINYVKQLEERVKGLGEDIKKKDTGSVSTITRSHILIDKDTAIDEMKTEECYGRNESLVEVEARVLGNEVLIKIHCGMQEGIVVNIMSQLQLLHLSVTTTNVLPFGNTLDIAIIAQASNY

>Phvul.002G007400.1-14_Pv

MDASEPNWFSDFETEDCNLFRQCQLEIEDDEELLSLEIASALENFPPPQHQQPFSSESHTSYSETSNLVRPAKQLKTNLSSWNSSPITDKHFSPNISSSTSSSPTSQIMLPVENTQLHGIVSAVLSPQQNKGVSVSPPETRKRSSENPNYETKSPKSQGSFKTSGHGGDHIIAERKRREKLSQSLIALAALIPGLKKMDKASVLGDAIRYVKELQERQKTLEENKNRDDVGSVVMVNKARLSYRDGCGRASLPRVEARVSEKDVLLRIHCQKQKGLLLKILVEIQNLHLFVVNSSVLSFGDSIIDITIVAQMGTDYKLTINELVKNLRRATLKSMS

>Phvul.002G007500.1-14_Pv

MMEIASTNYFPELEMEYLSFLDQYQMDSIACPLDDFDFESFSGSPESNSSYQLNSESTPNCFPAESPDLSVTPPRPTKRIKTFNTFKTCASHLISQNVSASPSSQIISFGHVNASPRGSHQFHNLHGRPKIENPSSENMDFTDFVSQGSYEGKSFLSSDNRTNLVGITTRNPIQAQEHVIAERKRREKLSQKFIALSAVLPGLKKMDKASVLGDAIKYVKQLQERVQTLEEKTAKSTTGSSVLLKRSILFADDENSDSHCDHSLPEIEVRVTGKDVLIRTQSDKHSGRAAKILSELEKLHFIVQSSSLLPFGNNNIDVTIIAQMKENYMAAKDLLGRLRQALKQVDGA

>Phvul.002G216700.1-14_Pv

MEDPNFLRQWHLSSIDDPNLLPVAAAFGETFQHHAFTYPDFNPKASMETTLTDIERPTKHHKNISWNPNKSAAQTSDTQFVSFPNLVSFMDSNHISPLGLVKANEMACPITNSTTSLDTISQGILGNHNYLFKACQETKKIGRRSKIYQPPDHIIAERKRREKLSQRFIALSALVPGLQKTDKASILGDAINYLKQLQEKVKALEEERNMKKTVESVVILKKPQLSNDGNTSSSEYDGPLFDGTIPEIAARFCERNVLITVHCEKTKGVVEKTIHEIENLHLKVTNSNAMTFGRCAIDITIIAQMDMEFCMEVKDVVRNLRSAFTSFM

>Phvul.003G231000.1-14_Pv

MMEESNKPMDEASAPSWLSDLDMDDYNLFPDECAMNLNLFDDQEFLPQDIVNALEKQTQTLQQSLSSECPSKTVSNSSTDDASFDFERPAKLLKTTSSSCCNSDSSTITNNLSPKLSPSTSFSSFQSQILSFDNSNSSPPNNTTQFYGFDLNPTQNEMVSVSVPQPTKSRLPNQTPKWSSKNQNFETKPSSHAKRSPAHAQDHIMAERKRREKLSQSFIALAALVPGLKKMDKASVLGDAIKYVKDLKERLAVLEEQSKKTRGESVVVLNKPDLSGDDDSSSCDESIDADSVSDSLFELESRVSGKEMLLRIHCRKQKGLLVKLLAEIQRNNLFVINSSILPFGDSIIDITVVAQMGENYNLTTKGLVKNLRVAAHKIMS

>Phvul.003G231100.1-14_Pv

MTSMEESWTSWLCDMEPDDYSFISQSDIKVEDMIGSLASPCDIATALEENPQSSFSQESHSSVTESGIEERPLKLLKKTCTSNSAKTDQLSQKKASTPSSYILSFDNTNPPTAKVESLVKPETKVNRGIALPSKNEPKRVTQESKKTGSSARSSHHTQDHIIAERMRREKISQQFIALSALIPDLKKMDKVSVLGEAIRYVKELKEKVKVMEEQGKRKSQEPVMRGKKSQVCATADEDVSDTSSNSCELGNSDDPSSKTNLSLPEVEARVSKKNVLIRILCEKEKVVLVNIFREIEKLHLSVINSSALSFGSSVLDTTIVAEMEDEFKMSVKELAGNLRVGLMQFM

>Phvul.003G231200.1-14_Pv

MTQISSTIYLPEFGMEDPTLFHQYPMDSFPFQLDDFDFESFSASPKGSSSHKRLSSESTQNSSLTQSAETSVAPPRPTKQPKTTMSTWSAYGTDMMAPKAASSSSSKIISFDKSNASSVSSQQSQQFYKLDAAKLLKKPKIEIGYGESLDFAAVASQSSYDNDCFLGYDKEEKKAAVTMARNPTQAQDHVIAERKRREKLSQRFIALSALVPGLKKMDKATVLEDAIKYVKQLQERVKILEEQTVDKTVESAVFVKRSVVFTRDDSSYSDENSNQSLPEIEARISGKEVLVRLHCDKHSGRAAAILRELEKHNLTVQSSSFLPFGNNTLDITIVAKMDKDYCLTAKDLIRNLSQCLRQLS

>Phvul.005G181300.1-14_Pv

MDFLLSPAFYSPMEQAWENWSLQMEKGDDGCKDLVLEECHTEEEEFLRDILGDPGFCSETRSHHNSATPTDGSSLSSDETLAMAAASNVNIPPQKSHIADSSPRTYILSFDTSTIIPPTPHFDTSKSSSTFTLSSKKRHRNLSSEQPKPNSATVKRGRNGSLDHIMAERKRRQELAERFIALSATIPGLKKTDKSSILCEAISYVKELQGRVTELEERNKRGKEAVMILNKSDEDSSNSSETDSKDWCRMLPDIEARVMENEVLIEIHCEKEEGVELKLLDHLENLHLCVTATSVLPFGNSTLGITIVAQMGDAYKMTMKEVVRNLREVLMNEINIHGDPY

>Phvul.005G181400.1-14_Pv

MEENQWGNCSSHMEMEDEVLNECLCQTNPFDEEFLRDILQQPEAEEGGDDMMMMMKNKNSNSSSMMNLCGEENKKKSGYILSFEGLTGWKDVSENSNSTTRSNQGTKKTRSASESMDHIMSERKRRQELTRKFIALASTIPGLKKMDKAHVLREAMNYVKQLQERVEVLEEDIQKNNAESAITITRSHLCIDDTNSDECYGSNEAVPPEVEARVLGKQVLIKIHCGKQKGILLKIMSQLEHLHLYISTSNVLPLGNTIDITIIAQMGDKYNMVVKDLVKELRRVAMMKLCVSQ

>Phvul.006G198400.1-14_Pv

MRSFNMEISSIRGLPQLGIMEDPNFLHQWQLNPADTTSLTGAAFGESLQKLSFSGNSNFNLKTTMETSPNGNERPTKQLRNNSWNSNKSQQQTPDTQYASCSNLLSFVNSNYSNELGLGKPKVEMVCPEIDNSNLAEMLISQGTLGNQNYHFKASQEAKKIETRPKLSQPQDHIIAERKRREKLNQRFIALSALVPGLKKMDKASVLGESIKYMKQLQEKVSTLEEEQNRKRTVESVVTVKKYQLSSDAEDSSSSETGGTLDEVLPEIEARICERNVLIRIHCEKNKGIIEKTISEIEKLHLKVTNSSALSFGSFILDITIIAQMDMEFCMTVKDLVRSLRSAFSY

>Phvul.010G042700.1-14_Pv

MEEDSWESWISSLEMGDEDNFIGHSHIKSLDEDKFVLEPLNFSFHNSSTNNNDNDNNSKKVFSMGSSCLSYEEDASFDERHGKMLKSNSSSSIISQDVVAYKNAASSSSSSSAPSSSSSFILSFENSTMKPSSLHHRPNDDDDHGDLHRYFGGDALCSSVLSSESEHVITKPKAKQGDNKKYRSSSEIQDHIMTERRRRQELTERFIALSATIPGLKKTDKAYILREAMIYMKQLEERVIELENENKRKNTDSRILIKKYSQVCSREEEGTSCDETKSHNISTPPLPQVEARVLEKEVLIGIHCHKQKDIVLKIMALLQNHHLSLASSSVLPFGTSTLKVTIIAQMEEKYCMTVNNLVKSLKQALLKIT

>Phvul.010G043200.1-14_Pv

DFANSSSNNNGSCLERNTQHANSNSEKNQVVSSCNLSFEDSTVVSNVPDKSFHEKPLKGRSSSLSQQHIMAERKRREKISTLLVELSAMVPGLKKLDKLSIIGKTIDYVKYLQNRVKDLQEQHSKMESIDCCRNNKSNVKISQNSNNGLDDRLTFPKVDASVSGKDVIIRVTCEKREHIVTKLLSMLASHNLSVVCSSVLPFGTSTLNISIIAKAQHFGMAIDDLVKNLSEDLLKLYNLQQ

>Phvul.010G043300.1-14_Pv

TMDETWEKWFSDLEMDDELHMFNEHNMNTLEEELMKVVSSVPCDSDFANSSSNNNGSCLERNTQHANSNSEKNQVVSSCNLSFEDSTVVSNVPDKSFHEKPLKGRSSSLSQQHIMAERKRREKISTLLVELSAMVPGLKKLDKLSIVGKTVDYVKYLQNRVKDLQEQHSKIEYAECCRNNKSNVNISENSNNGLDDRLTFPKVNVSVSGEDVIIRVTCEKREHIVTKFLSMLASHNLSVACSSVLPFGTSTLNISIIAKAQHFGMAIDDLVKNLSEDLLKLYNLQQ

>Phvul.010G043400.1-14_Pv

MDQMDDESWQKWVPIMENNDWHIFSDFDMNSAGEELKEDDSSVDPEEVSWKENTATLLTNFLSLEENTAFDMPSLTQQASPHKPQTCVLSFEDSTSVPTVAKKTCQPNLGEQPKQTQEEKPHHRKSLKRERTSPQTADHILTERKRRENITRMFIALAALIPGLKKMDKVSVLSNAVEYVKYLQQRVKDLEKEKQQKRKIESAGCLKLSKTNMVAYDLSWSSHVYDGAKTTKKCSKVEARVAGKDVLIRVTCEMQKNIVRDVMAKLQAHNLSILCSNVLPFGNSALAITTIAEMNPKFTMAVEDLVKKLNDDLSKCCSLQY

>Phvul.010G158200.1-14_Pv

MEESGENWPSDSYLEVGDDVKDSCFDDEESVSEEDDGDSFSEMKTINRKQRQQKLGERFLALSATIPGFQKMDKTSILDKASKYVRELEERVRELEQEVESNKVCVSSSSSSWEVNSNNGYCHGSNQDLEVKVRVLQKEVLIIVHCEKQKGIMLKILSHLENINLSVINSSVLRFGNSTLDITIIAQMGEGYEMGVDELVKSLRLAILTQ

>Phvul.010G158300.1-14_Pv

MMEDSWQNWLSHLGMNDNFGCSSNVCFEEMVWLDNGQGCDRSNNEVGCMGMKEEHSECCCSKRGVGKHEELEEKGREKRGTKRARTSSEIEYHIISERKRRQDIAEKFIALSATIPGLKKIDKASILGEAIKYMRQLQQRIAVLEKASNNNNSAKSLIITKSHLCSTSCEQNSNIEVEARGLENEVLIRIYCEQRKDIMLNLITLLKDLHLSVTTSTVLPFGNSLLNIIIVAQMSEEYNLTITDLVKTLKQNLFKFCEV

>Phvul.010G158400.1-14_Pv

MEEDSCNNNSHLHMEMDFFDNNDFDDFFSDIIFHQTPPPTPFSSGSESDHSFRASSFLPSAAVNAAASKRSSPRTYILSFDNSTVVPATPEPSLPSSPLPAKRALHSQNPTTRPNQGSKRSRTSSQTIDHIMAERRRRQELTERFIALSATIPGLSKTDKASVLRAAIDYVKQLKEKVQELEKESRKSAEETVILVNKSNPNGNEEITNSSTETNCSILPEMEARVLGKEVLIEIHCEKEYGVELKILDHLENLHLCVTGSSVLPFGNSALCITITAQMDEERQMTVNEVVRNLREVLSVSHLVSDSDPY

>Phvul.010G158500.1-14_Pv

MEEDSCNNNNSHLHMEMDFFDNNDFDDFFSDIILHQTPPPTPFSSGSESDHSFPASSFLPNAAAKRSSPRTYILSFDNSTVVPATPEPSVPSSPLPAKRALNAQNPKTRPNQGSKRTRTSSQTIDHIMAERRRRQELSERFIALSATIPGLNKTDKASVLRAAIDYVKQLKEKVQELEKESRKSAEETVILVNKSNPNGNEEITNSSTETNCSILPEMKARVLGKEVLIEIHCEKEYGVEIKILDHLENLHLCVTGSSVLPFGNSALCITITAQMDEQCQMTVNEVVKNLKQSALSVSFGER

>Phvul.010G158600.1-14_Pv

MFNESLFSAIPIPIYYPPFSSQTLHTNLHHSLPVLSFLLSSSLQMGEPCHKYWFSDMEIQDFDFFNQSHHKIESVDDEFLFSSQHAQEKPATATTTTSPTAYILSFNDSTVVAATCQTYGGKQPYQEEVVVGSGGACLPSKGVSEKHYIEPKPNPATRKGRSSAETLDHIMTERKRRRELTERFIALSATIPGLKKIDKATILCEAITYVKRLKERVRELEEQCKRTRVESVSFVHQRSNNGSDKGTTSSGAMKSDECYKTSEALPTVEARVFKKDLLIRIHCKLQNGILIKILDHLNTLDLYTTSNSVMPFGSSTLDISIVAQMGDKFNATMNDLVKNLRLVLLQSCEVQQ

>Tp57577_TGAC_v2_mRNA17469-15_Tp

MENISFIRGFPDQEIVEDPSLCLHQWHLNSIDESNSLPIASAFGDTLHHHSYIYPNFNRRTSVETAPKLDTQFVSYPNLVSFVDLNHVNQFGLTKPKDEMVCPQNSKTAPSDMIPQGTFEAKKLATRPKLSLPQDHIIAERKRREKLSQRFIALSALVPGLQKMDKATVLGDAIKYLKKLQEKVRDLEEEQNMKKNVETVVVVKKSQLRNDVENSSAESGGPFDEELPEIEARFCDRNVLIRIHCEKSNGVVEKTIHEIEKLHLKVTNSSVMTFGNCALDITIMAQMDKEFYMTVKDLVRNLRSTFASFI

>Tp57577_TGAC_v2_mRNA18047-15_Tp

MSSHMEISSTRGLPELGLIEDPNFLHHWNNHLNSIDTNNLTASAFGDALQKHFLSNNPNFNNKTCMETSPTGSERPVKQLKNNSWNYNNSPQTSETQYANSCSNNLLSFADSNYENQLGMLKPKSEMVCPKIDNNSTLANMFGNQNHVFKAFQEAKDIETTRPNKLSQAHDHIVAERKRREKLSQRFIALSALVPNLKKMDKASVLGEAIRYLKQMEEKVSVLEEEKKRKKSVESVVIVKKSQLSTTNDADSSDNTTFDDTLPEIEARFCERSVLIRLHCLKSQGVIEKTISEIEKLHLKVINSSALTFGNFTLDITIIAQMDVGFCMTLKDLVRKLRSEYSSSM

>Tp57577_TGAC_v2_mRNA19805-15_Tp

MEQSWEDIWHLHTEMDIENWLEMCQEEDYLGDYMVHQLSLEAPAMAGAATENTLQEICHIPTVVSSEMTKQLPHPSTSSKTCTVSFDNSADIPLKPSVISSKPPRPPCSAKKRTSEKLKSTEAKAITKEGEKKIMVERKRRLELAHMFIELSATIPRLKKTDKASIVMGAINHVEQLKKRVKELEQQNNIGKEPIRLHNELLPYVKAKVLDKNILIYINCEKQNGIELNILEMLENLHLFVTSTSVLPFGSSTLAITIIAKMDDAYKMKMVDLVDNIRQLLSNGSANHPT

>Tp57577_TGAC_v2_mRNA19816-15_Tp

MDQSWENWPLHSEFGDDEEFLRDIILQPPVTYSGSSLSSSSNEMDGSDWSGKKVHNPSTTLSSNTPGPRTFILSFDKSTIIPATTTQDEDEDEEEEEEPRIKSRSCGKSNNNNNNKRSLELPKVKVSSSTKSGKKSRSDSQCMDHIMAERKRRLEIAQKFIALSATIPGLKKTDKTSILCEAINYVKQLQERVKELEDPNKKSKESTLINLKTNEKSVVDVKARVLGNEVLIEMHCEKENGIELKILNHLQNLHLVVTASTVFPFGNSNLGFTIVAQMGDGYKLTVDDLVKTLQQVPLINMRRST

>Tp57577_TGAC_v2_mRNA23188-15_Tp

MEEINNTPMNVAADSNWLSDLEMVDEYILFDEDCNLNLLDANQEQFLSHDIASAFEEQRQTLQQCLTTEFISTTMSKIFTDETSFESFDDFDFEKLAKELKTIDQSNNAENFSPQFSASTPSIQSPQILSFDNPNPTEFYSFDQTQNEMVTSLSLPELGNIQFSTQISKASSKNQNLETKTSQSKRPRAHGLDHIMAERKRRENLSKSFIALAALVPGLKKMDKASILADTIKYLKELKERLAVLEEPGKKTNEDQSMVVTTKPDVCSDQHYSSSHENTETESAADGIATGQSLFKVEAKVKGKDMLIRIHCQKYDGLLVKIITEIQSYQLSVVNNSVLAFGDSILDITIIAEVKIGEGYNLSIKGLVKHLSIAALEFMSS

>Tp57577_TGAC_v2_mRNA23191-15_Tp

MAKRKSISLCSKEQQFSTPQSVTMEEINNTPINSSFQSLILSFDNPLNSPPTTNTPHIYGFDPTLNETESLSPPQFGNKHVSTQIPKGSSKNENLEAKISQNKRSRYHGNDHIMAERKRREKLSQSFIALAALVPNLKKMDKQSILTDSIKYVKVLKQRLEEQNKTTNADSVVVMSKQDLCINNDNSSYDESIGGAFDANESLLQVEARVSGNMGSLQERLLKISQKKQKCVKASGVGGSSSSAVAEDDVIVVDQSKETRRPKNRASSVVEDPERVKRTRVDLEFGDKEVDKFILPACIGNNGLLDRNITVQISPASLKRKHTKLKNDFLNYKEKFKVHASIILDNTKKEEEIKELRRSQETWVEEKTKLEDFLKSYGSRTLDGEVSTVDRSDELAGLDRATLIAKIHNLEGEMLNSAQESFDNAFSQVKCLNPKIELKTEGMSVFMVVDGDRLTLPVGQDEAEDV

>Tp57577_TGAC_v2_mRNA23192-15_Tp

MEESWTNWLCDMEPDDYRFINESSINTDINQVSSLERPSKLLKTASSSSSSPCSYILTFENENPPPIKVQPALKTKTKVVNSKNEQRRVNTQESNMKKNSFSKSTTHHTPDHIIAERLRREKISQQFIALSALIPNLKKMDKASVLGDAIKYVKELKEQVKVLEEQTKKKSVESVMVVKKLSQLYVDEDVSDTSSNSCDENSDETSKTYLSSLPEVEARLSGKNVLIRILCEKDKGVMVNVYREIEKLHLSVIDASSFSFGSSVLAITIITQMEEELNMSVQKMAKQLRFGLVQLN

>Tp57577_TGAC_v2_mRNA23194-15_Tp

MIVSCLPKGFRFEASFKEKHFSFTMEEINNTPINNVSETSSWLSDLEIDECNIFPEECNLNFLDADEKEEFLSHDIATVFQEQTQQQCLTSESTLNNSFTDETNFDSFDFDFEIERPTKQLKTIDQSENIIETLSTKLSPSFQSLILSFDNPGNSPHTTNTPQIHGFTPTLNSKQNETVSVFPPQSGNMNVSTQIPKGSSKNQNLETKTSQNKRSRAHGHDHIMAERKRREILGQSFIALAALVPNLKKMDKASVLTDSIKYVKELKERLKVLEEQNKKQEAESVVIPNKPNLCSDFDNPSSCDESIEGAANANESVLQVEARVSGKEMLIRIHCQKQKGLLVKIMAEIQSFQLFFVNSSVIPFGDSMDITIISQMGEGYNLSIKELVKNLRMAALKFMSSWH

>Tp57577_TGAC_v2_mRNA23195-15_Tp

MEEINNTPMNSSETSKWLSDLEMDEYNLFSEECNLNFLDAGEEEFLSHDIATVFQEQQNQQQPLTSESTDETNFESFNFYFEIERPIKHMKTTNDSDKITETLSPKLSPSSFQSLILSFENPPNSPPTINTPQIYGFDPTLNSKQNETESLSPPQLGNIHGSSQIPKGSSKNENLETKTSQNKRSRNHGHDHIIAERKRREKLSQSFIALAALVPNLKKMDKASVLTDSIKYMKELKQRLEVLEEQNKKKKAESVVVINKQDLCISDDNSSCDESIEGADIDANESVLQVDARVSGKEMLIRIHCKKHKGLLVKIMVEIQRFELFVVNSSVLPFGNSILNITIIAQVKDSFYLP

>Tp57577_TGAC_v2_mRNA23200-15_Tp

MIQISSTNYMPQLLGMEDALFNDHDYPMDSFAFEYDDMAYFKSFSESPPESSYSSPNNNHKRFHSESTQNSSFPIHSSPDQSVASATPPTKLLKASPKIISFDHSDTSSARQFYDVDAKVKKPKAEIEYGENLNFAALISQGENKVANRNPIQARDHVMAERKRREKLSQRFIALSSILPGLKKMDKATILEDAISHLKALNERVKNLEEQVADKKVESAVFVKRSIIFADDDSSSCDENSDQPLPKIEARVSGKDMLIRIHCNKNTGRTATTILNELEKHHLTVQSSSILPFGNNYLDITIIAQMNKEYCLTIKELIRSISHVLRKLI

>Tp57577_TGAC_v2_mRNA23501-15_Tp

STDESWTTWLCDLEPEDYNFMDDINITPLPEEPENLQQTLSSGSHCSQTTSSTMSNSSGDVVNSFEERPTKILKTTPSNTGYPTQKKDPSHSYFLCFNNENPETEPNPILNIDSSLKPKAQILNYDGKSENKKKEPKRNIQESKKTDSVTRNAKDHIIAERKRREKISQQFIALSALIPDLKKMDKASVLADAITHVKQLQEQVKVLEEKNQRINKESVVYVEKTKSCSSDEDVSDNTSSNSEYGNCCHPSRSLPEVEARVSEKNVLIRIHCEKQKGVLMNIIQEIENLHLSVTSSSTLQFGTTKLDITIIAEMDEEFSLSVQELARNLQVGLLQFMSL

>Tp57577_TGAC_v2_mRNA26902-15_Tp

MDKLNTSSNTSSTSWLSNMLFEEIDIEGCGDLFQQCEQNLFGDEEFLTHDIASVLQQDEILLEQSESYSMERPNKKLKPNTILQDVSPVSSPSSTTSQILSFDCTLNTTKQNEVVPLSSQKHVQLPQNRGSKRSVAHNQDHIIAERKRREKLSQCLIALAALIPGLKKMDKASVLGDAVKYVKELQERLKVLEEKNKNSNSTSPVECVVTMNNGPQLSYDSSSNDESEAEAGSGNNEVLEPHVDARILDKDVLIRIHCKKQKGLLLKVLFEIQKLNLFVVNSSVLPFGDSILDITIVAQMGTGYKLTRNGLIKSLRVAALRYMS

>Tp57577_TGAC_v2_mRNA26903-15_Tp

MMEIMSPIYLPELGMEDLNFLHQYPMDSLASDAFDNFGFHFDNNYETTPNCFPVETHQPNDNYVAQTRPTKKIKTSSTNPSSYSSTPQLISFEQFNATPIVSNKEFYGLDYSDVKPKFEKGCSTNKDFPANYDNRANQTRNTTQAQEHVMAERKRREKLTRSFIALSAIVPGLKKMDKASVLGDAIKYMKQLKARLQTLEEQVEDNKKVGSTVQVKRSVIFIDNNDHDDNNKRTLLPEIEVRVSSKDVLIKIQCDKQSGRAATVLGQLENLNLIVQSSNFLPFGNNMLDVTIVAQMNKENCVTAKDLLGSIRQALIN

>Tp57577_TGAC_v2_mRNA29165-15_Tp

MEESLENLISYMEMEDEVLNPNSFDEQEFLKDIILDQPECEPSSYLCSNKIQNIGTINGEGIATSPINSILSFDETSLIHKSTSSNSIMSLERSNVGSPPASYLLSFDNSSVEPIVEPMSHKKRTIVKDKTTKKVKRSCETVQDHLMAERKRRRELTENIISLSAMIPGLKKMDKCYVLSEAVSYTKQLQNRIKELENQNIVNSAIFKWKSKGSSNKKKYLESLLEIEARVVEKKVIIRIHCEKQKDIVLKIHELLEKFNLTITSSSILPFGASILVINIFAQMDEEHSITMDDIVKNLKNYLLEACESR

>Tp57577_TGAC_v2_mRNA40009-15_Tp

MEDSSWENFHLHTEMECGDDYFMEKYNAVNIDGDDFLREILLQTPEQSLMNSESENGSVTVNVNGDVDVAGNMVKSNSSNSIVSQKQEQQQRLKSKKVDVPRRSSSPTTYILSFDNSTMVPATPEPCVNLEGGKRDYCSKSDYSKKNKQSSEIKKTNTKNQGVKKARSGTQCVDHVIAERKRRQQLTERFIALSATIPGLSKTDKASILRSAIDYVKQLQERVHELEEKQDKNVGLTSSVMVLNKINSCGINNNIEGTNSRDTSCDGDCSNIFPEIEVRVMGKEVLIEIHCEKQSGIELKLLDHIENLQLFVTGNSVLPFGKSAISITIIAQMGDGYKVKVHDLVKSIRQVLLKPQIRCESDPY

>Vang0027ss00450.1-16_Va

LQNQHFCFLLSHGASMAELLSSNGKNRNTRHGDLMLEECHTQEEDFLREILGEPGFSSETETHHHNFTSPTDAETLTMELTERFIALSATIPGLKKTDKSSILGEAINYVKELKERVTELEERNKRGKESVMILKKSDVCESSERDSKDWCRMLPDIEARVMENEVLIEIHCEKEEGVELKLLDHLENLHLCVTATSVLPFGNSTLGITIIAQMGDAYKMTVNDVVKNLRKVFMNHMNINGDPY

>Vang0027ss00510.1-16_Va

MEENQWGKLSSHMEMEDEVLNECLCQTKTFDEEFLRDILQQPQEGAAPDLNTFGETSCVIRAGGGDKLMMKNSNSSMMNNLCGENKNKATASPTTYVLSFDKSDDPHSVSAWKDVSHKLPLSSTRTNQGTKKIRSASESMDHIMSERKRRQELTRKFIALASTIPGLKKMDKAHVLREAINYVKQLKERVEVLEEDIQKNGAESSITITRSHLCIDDTNSDEWYGSNEAVPAEVEARVLGKQVLIKIHCGKQKGILLKILSQLERLHLLISTSNVLPLGSTIDITIIAQMGDEYKMVVKDLVKELRQVAMMKSCVSQ

>Vang0064ss00600.1-16_Va

MEITSIRELPDMKEQEMMEDPNLLRQWHLSSIDDPNLLPIAAAFGETFQQHAFTYPDFNPKASMETTLMDIERPTKHHKNISWNPIKSSAQTSDAQYVSFPNLLSFMDSNHISSLGLVKANEEACPITNSTTSLDTFSQGILGNHNYLFKACQETKKIGRRSKISHPQDHIIAERKRREKLSQRFIALSALVPGLQKTDKASILGDAISHLKQLQEKVKALEEAQNMKKTVESVVIVKKSQLSYDVNNSSEYDGPLFEETIPEIEARFCGRNVLIRVHCEKTKGVVEKTIHEIENLHLKVTNSNAMAFGRCAIDMTVIAQMDMEFCMGVKDVVRNLRSAFTSSM

>Vang0069ss00620.1-16_Va

MHRPAFSSDSETHSPKVQSKSTYGGSDYTALTSSKSSNLVMKTNSSNNFLFSSQLAPEKPATAISPTAYILSFNDSTVVAATCQTYDGKQPYQEDAVAGSGGACLPSKGVSEKHEIEPKTNSATRKGRSSAETLDHIMTERKRRRELTERFIALSATIPGLKKIDKATILCEAITYVKRLKERVRELEEQCKRTRVESVSFVHQRSNIISTDKGTTSGATKSDECYKTNEALPTVEARVFKKDVLIRIHCKIQHGILIKILDYLNNLDLSTTSNSVMPFGSSTLDISIVAQMGEKFNATMNDLVKNLRLVLLQSSEVQK

>Vang0069ss00640.1-16_Va

LKSMEESGENWPSDSYLEAGDDVKDFCFDDEEFASEEDEGDSFPEMKSTDRKQRQQKLRERFLALSATIPGFQKMDKKSILDKASNYVKQLEQRVRELEQEVQSNKVCASSSSSSEVNSNGSNQILPEVKKHQEKTQPHALMMEDSWQNWLSHLGMNDNFGCTSNNGVLFEEMMWLNSTGQGCDSSNKEVACMEVKEEHSKCCWRKRGVEKHQELEAKGREKKGTKRARTSSEIEYHIISERKRRQDIAEKFIALSATIPGLKKIDKVSILGEAIKYMRQLQQRIAVLEKASNNNSVKPFIIAKSHLCSASCSANSNIEVLPEVEASGLENEVLIRIYCEKRKDILLNLMTLLQSVHLSVTSSSVLPFGNSLLNIIIVAREMDCQNEFFYDYDPINDDCDDFFTDIILQPTPPTPFSSESESDHSFRASNTVQTTSFLPSAAVNAVASKRSAPRTYILSFDSSTVVPATPEPSVPSSLLPAKRALHTQSPTTRPNQASKRTRTSSQTVDHIMAERKRRQELTERFIALSATIPGLNKTDKASVLRAAIDYVKQLKEKVDELEKQNRKSAAETVILVNKTDSNGNEDSTNSTETNCSMLPEMEARVLGKEVLIEIHCEKEYGVELKILDHLENLHLCVTGSSVLPFGNSALCITITAQMDEEYEMTVNDLVKNLREVLSKSHLVSYSDPY

>Vang03g04780.1-16_Va

MRDHKTRNHPWICEAIIKKFQCLVGEEHCQSPQNNNRWRIFSSAVFHCMRSFHMEISSIRGLPELGIIEDPNFLHQWQLNPTDTSSLTGAAFGESLQKLSFSGNSNFNPKASMETSPNGNERPTKQLRNNSWNGKKSQHQTPETQYASCSNLLSFVNSNYINELGLGKPKVEMACPKIDNSTLAEMLISQGTLGNQNYHFKANQEAIKIETRPKLSQPQDHIIAERKRREKLSQRFIALSALVPGLKKMDKASVLGEAVKYLKQLQEKVSTLEEEQNRKRTVESVVTVKKSQLSDDAEDSSSSDTGGTLEEALPEIEARFCERNVLIRIHCEKKKGIIEKTISEIEKLHLKVTNSSALSFGSFILDITIIAQMDMEFCMTVKDLVRSLRSAFSYYWLSRQSNTLKVSGSSPGDAICFCFDFSKSNNLGKRRRFNFEFRSHKSRVIWGKLRLMEKMYENISATFRPLRPPRIATNHISPIISFSILIGPRHQPRTFFPKTNNCIYTNAFVILSPKPTRENTFIIIERIISNRTMKSLLLFGIAVLLFHSCLASEIPYPKAISDLKDSIMKGFGITAEDEVKITGFDPREAEVGHSVEYRFDLEIDHQVIPFKLLEDVKRWDYVDLPIFQSQDQAHTGLVPKRASDNRLPVLAPFVLAGPMELWIHDANDMRLSLPHDVDAGVLKKVILAEGAAVTVKGARSVSLRQPLEFPLPLNRTENGFANGLLALAGHLRHASRTQASPILSLRIVGPTSIAAAPSDSTTSTSLKLKRLAPGLVELSSPSKTKEVEPFSSVDLEGEAPTILTPTQFSTLWPLSSINGSNANLLGFEKLLHSVLGHKADKKGSFRLLKADVSAQTYVKIGFKAEKKVNEEDLEGYPAWRTKPETLTTHFEVLAKVDGERVVPEKVMQLKPVVPVDNVAPNLLAGNVSMSKMQDVHPPPNPFSLLSMAFSFFHEFFAIFSSAVFHCMRSFHMEISSIRGLPELGIIEDPNFLHQWQLNPTDTSSLTGAAFGESLQKLSFSGNSNFNPKASMETSPNGNERPTKQLRNNSWNGKKSQHQTPETQYASCSNLLSFVNSNYINELGLGKPKVEMACPKIDNSTLAEMLISQGTLGNQNYHFKANQEAIKIETRPKLSQPQDHIIAERKRREKLSQRFIALSALVPGLKKMDKASVLGEAVKYLKQLQEKVSTLEEEQNRKRTVESVVTVKKSQLSDDAEDSSSSDTGGTLEEALPEIEARFCERNVLIRIHCEKKKGIIEKTISEIEKLHLKVTNSSALSFGSFILDITIIAQMDMEFCMTVKDLVRSLRSAFSY

>Vang04g04640.1-16_Va

MEDSLENWISDLGSCSKRGGAVVKDGSQFEPVMAQPRKRARRSCETQHHIIAERKRRQELTGSIIALSATIPGLKRMDKAYVLREAVNYTRQLQERVKELENKNSEKRVVHHSSTLVRKSEVCSKKSLSNSSENNKESLFEVEARVLDEEILIGIHCEKQKDIVCNILAFLEKLHLSPTSSSVLPFGTCTLIIHIIAQMDEECRMNMDELVKNLREYLLNVYDMQQVSL

>Vang07g03330.1-16_Va

MKRVRCPLYKTFPTTSFFSSPNFTTLISSLQSPPSPPLFEFSIQGLYKRRKLKMMEIASTNYLPELEMEYPIFLDQYQMDSFACPLDDFDFESFSGSPESNSSYRLNSETTLNCFPAQSSDQSFTPPRPTKRLKNTFNTFKTCAGDSIPHNVSASPSSQFISFGHFNASPRASQQFHNFHVKPKSENPSSEHMDFTDFVSQGSYQDKTFFSSDNRTNQVGLTTRNPIQAQEHVIAERKRREKLSQRFIALSAVLPGLKKMDKASVLGDAIKYVKQLRERVKNLEEKSAKTTTGSSVLVKRSILFADGENSDSHCANSLPEIEVRVSGKDVLIRTQSDKHSGRAAVILSELEKLQFIVQSSSLLPFGNNNIDVTIIAQMKENYMAAKDLLGRLRKALKQVDGA

>Vang07g03340.1-16_Va

MFRQCLEDDHELLSLEIASALENLPLPQHQQPLFSESHMLYSETTNFNSSINQPKTNLCSWNSTPISTKHFSPKLSSSSSSSSSPPSQIMFLNNSNSLSVENTQFQGIVSAALSPPQQNKGVSVSIPENGTRSSKNLKDKGIRKTSGHGRNHIIAERKRRQKVSQGLIALAALIPGLKKMDKASVLGDAVRYVKDLQERLKNLEGKNKKKRDEKSEVVMVKKPRLSYHDGSASGDGDGCFRESLPRVEARVSEKDVLLRIHCEKRKGLLLKMLVEIQNLHLFVVNSSVLRFGDSNLDITIVAQMGTEYNLTINDLVKNLRMATLKST

>Vang08g06540.1-16_Va

MDDESWQQWVSILEENDWHTLRDFDLNSAGEEFKGDNSVVPAEDSLKESSTTLLSNFPSLEENTGFDTTSVTQQHASPRPKSCVLSFENFTSVPIFDKKTCQHLGEHSNQTQEKTRKPLKRERNSSQTLDHIMSERKRRENLTKMFIALSALIPGLKKVSSYSFSFAS

>Vang08g06590.1-16_Va

MDQMDDDSWQQWVSILEENDWHTLRDFDLNSAGEEFKGDNSVVPAEDSLKESSTTLLSNFPSLEKNTGFDMTSLTQQHDSPRPKSCVLSFENFTSVPIFDKKTCQHLGEHSKQTQEKTRKPLKRERNSSQTLDHIMAERKRRENLTKMFIALSALIPGLKKVEARVAGKDVLIRVTCEMQRNIVQNVMAKLEAHNLSVVSSNVLPFGNSAITITSIAQMNPKFTTTVDNFVKXLTEDLSKCCNL

>Vang08g06730.1-16_Va

MEDSWENWISSLEMAEDNSTDHSHINEDNFLTGIVLEPLDFSFLNSSTTTTMLSYDDNHDNKNDHLSTPKAKQGAKKYRSSSEIQDHIMAERKRRQELTERFIALSATIPGLKKTDKAYILREAMIYMKELQERVIELEKQNKRKRSTESRILIKKYSQREEESKCDRSNLPLPHVEARVLEKEVLIGIHCHKQKDIVLKIMALLQNHYLSLASSSVLPFGTSTLKVTIIAQMDEQYCMTVNNLVKSLRQALLKIT

>Vang11g05040.1-16_Va

MTQISSTVYLPDFGMEDPTLFHQYPMDSFPFQLDDFDFESFSASPKGSSSHKRLSSESTQNSSLTQSPETSVAPPRPTKQPKTTMSTWSAYGTDMIAPKAASSSSSKIISFDKSSASSVSSQQPYKLDDAKLLKKPKIETGYGENLDFSAVASQSFYDNNSFLDYDKQEKKAAVTMARNPTQAQDHVIAERKRREKLSQRFIALSALVPGLKKTDKATVLEDAIKYVKQLQERVKILEEQSVDKTVESAIFVKRSVVFSGDDSTYSGENSDQSLPEIEARISGKEVLVRLHCDKHSGRTAAILRELEKHNLTVQSSSFLPFGNNTLDVTIVAKMGKDYCLTAKNLIRSLSQCLRQLS

>Vang11g05060.1-16_Va

MMEESNKPMDDASAPSWLSDLDMDDYNLFPDECALNLNLFDDQEFLPQYIASALEEQTQSLQRSLSSECPSKTVSNSSTDETSFDFERPAKLLKTTSSSCCNSDSSTITNNLSPKLSPSTSFSSFQSHILSFDNPNSSPPTTTTTTHFYGFDLNPTQNEMVSVSMPQLTKSRFPDQTPKGSSKNHNFETKPSHGKRSPAHAQDHIMAERKRREKLSQSFIALAALVPGLKKMDKASVLGDAIKYVKDLKERLAVLEEQSKKTREESAVVLNKPELSGDDDSSSCDESIGADSVSDTLFEVESRVSGKEMLLRIHCLKQKGLLVKLLAEIQRNNLLVINSSILPFGDSILDITIVAQMGENYNLTMKELAKNLRVSANKVVS

>Vradi04g07860.1-17_Vr

MEDSLENWISDLLGSSLKGSCSKRGGAMVKDGSQFEPAMAQPRKRVRRSRETQHHIIAERKRRQELTGSIIALSATIPGLKRMDKAYVLREAVNYTRQLQERVKELENKNSEKRVVHHSSTLVRKCEVSEKKNLSNSSESNKESLFEVEARVLDEEILIGIHCEKQKDIVCNILAFLEKLHLSPTSSSVLPFGTCTLIIHIIAQVQIEFFELLK

>Vradi04g07870.1-17_Vr

MDDFFNQSHMVHCNEDESLKDIIVFSPESNIPSSSSLQSNFNSKTSSFSAAHVFCFDNPNSEAHQNHPSKSTTQSQNAISRADRQHHILAERKRREELTNNIVELSAIIPGLKKRDKGSVVREAVDYVKRLKERVKELENQKKDNMNSIGSLTKKPTLSISVEEELPEMKISVSDREVLIGIFCHNPNNTLVKVLSLLDNLHLSTTCSSVLPFGTSTFKVTIIAKMNDEYSMTIDDLIKAVGGCLLNLKSQKVLK

>Vradi05g16320.1-17_Vr

MSSYDSGMWPGTVITKPFDEEFLRDILQQPQEGAATGLNSFGETSCVVRAAGGDKLMMKNSNSSMVNLCGENKNKATTSPTTYVLSFDKSDDPHSVSAWKDVSHNLPLSSTRTNQGTRKTRSASESMDHIMSERKRRQELTRKFIALASTIPGLKKMDKAHVLREAINYVKQLKERVEVLEEDIQKNGAESSITITRSHLCIDDTNSDEWYGSNEAVPAEVEARVLGKQMLIKIHCGKQKGILLKILSQLERLHLLISTSNVLPLGSTIDITIIAQMGDEYKMVVKDLVKELRQVAMMKSCVSQ

>Vradi05g16330.1-17_Vr

MLEECHIQEEEFLREILGEPGFSSETETHHHNNFTTPTDAHTLTMTDKSSILGEAINYVKELKERVTELEERNKRGKESVTILKKSDVCESSERDSKDWCRMLPDIEARVMENEVLIEIHCEKEEGVELKLLDHLENLHLCVTATSVLPFGNSTLGITIIAQMGDAYKMTVNDVVKNLRKVFMNHMNIHGDPY

>Vradi07g11610.1-17_Vr

MTQISSTVYLPEFGMEDPTLFHQYPMDSFPFQLDDFDFESFSASPKGSSSHKRLSSESTQNSSLTQSPETSVAPPRPTKQPKTTMSTWSAYATDMIAPKAASSSSSKIISFDKSSASSVSSQQLYKLDAAKLLKKPKIETGYGENLDFSAVASQTFYDNNSFLDYDKHEKKAAATMARNPTQAQDHVIAERKRREKLSQRFIALSALVPGLKKTDKATVLEDAIKYVKQLQERVKILEEQTVDKTVESAIFVKRSVVFSGDDSTYSGENTDQSVPEIEARISGKEVLVRLHCDKHSGRTAAILRELEKHNLTVQSSSFLPFGNNTLDITIVAKMGKDYCLTAKDLIRSLSQCLRQLS

>Vradi07g11630.1-17_Vr

MMEESNKPMDDASAPSWLSDLDMDDYNLFPDDCALNLNLFDDQEFLPHDIASALEEQTWSLQQSLSSECPSKTVSNSSTDETSFDFERPAKLLKTTSSSCCNSDSSTITNNLSPKLSPSTSFSSFQSHILSFDNPNSSPPITTTTTHFYGFDLNPTQNEMVSVSLPQLTKSCFPDETPKGSSKNHNFETKSSHGKRSPAHAQDHIMAERKRREKLSQSFIALAALVPGLKKMDKASVLGDAIKYVKDLKERLAVLEEQSKKTREESVVVLNKPDLSGDDDSSSCDESIGADSVSDTQFEVESRVSGKEMLLRIHCLKQKGLLVKLLAEIQRNNLLVINSSILPFGDSILDITIVAQMGENYNLTTKELVKNLRVSANKVVS

>Vradi07g14230.1-17_Vr

MEDSNLLRQWHLSSIDDPNLLPMASTFEETFQQHAFTYPDFNPKASMETTLMDIERPTKHHKNISWNPIKSSAQTSDAQYVSFPNLLSFMDSNHISPLGLVKANEVACPITNSTTSLDTFSQGILGNHNYLFKACQETKKNGRRSKISHPQDHIIAERKRREKLSQRFIALSALVPGLQKTDKASILGDAISHLKQLQEKVKALEEAQNMKKTVESVVIVKKSQLSYDVNNSSEYDGPLFDETMPEIEARFCGRNVLIRVHCEKTKGVVEKTIHEIENLHLKVTNSNAMAFGRCAIDMTIIAQMDIEFCMGVKDVVRNLRSAFTSFM

>Vradi09g00080.1-17_Vr

MFFHDSNSHRLHLLTLFTHSLILQLDPTTFSLLKSMEESGENWPSDSYLEVDDDVKDLCFDDEEFASEEDDGDSFPEMKNTDRKQRQQKLRERFLALSATIPGFQKMNEKKDKTSILDKASNYVRQLEQRVRELEQEVQSNKVCASSSSCYEVNSNGANAILPEVKVRVLQKEVLIIVHCEKQKGIVLKILSHLENINLSVLNSSVLRFGKSTIDITIIAQMGEGYQMGVDELVETLRLMILNQ

>Vradi09g06160.1-17_Vr

MAERKRRQELTERFIALSATIPGLKKTDKAYILREAMIYMKELQERVIELENQNKRKRTAESRILIKKYSQREEERKCDRSSPPLPQVEARVLEKEVLIGIHCHKQKDIVLKIMALLQNHHLSLASSSVLPFGTSTLKVTIIAQDH

>Vradi10g11920.1-17_Vr

MRSFHMEISSIRGLPELGIIEDPNFLHQWQLNPANTSSLTGAAFGESLQKLSFSGNSNFNPKASMEASPNGNERPTKHLRNNSWNGKKSQHQTPETQYASCSNLLSFVNSNYINELGLGKPKVEMACPKIDTKIDNSTLAEMLVSQGTLVNQNYHFKANQEAIKIETRPKLSQPQDHIIAERKRREKLSQRFIALSALVPGLKKMDKASVLGEAVKYLQQLQEKVSTLEEEQNRKRTVESVVTVKKSQLSNDAEDSSSSDTGGTLDEALPEIEARFCERNVLIRIHCEKKKGIIEKTISEIEKLHLKVTNSSALSFGSFILDITIIAQMDMEFCMTVKDLVRSLRSTFSY

>Vradi11g03750.1-17_Vr

MMEIASTNYLPELEMEYPTFLDQYQMDSFACPLNDFDFESFSGSPESNSSYQLNSETILNCFPAQSPDRSFTPPRPTKRLKNTFNTFKTCAGDSISHNVSASPSSQLISFGHNFHLKPKSENPSSENMDFTDFVSQGSYQDKTFFSSDNRTNQVGLTTRNPIQAQEHVIAERKRREKLSQRFIALSAVLPGLKKGYAITNLRFSEQTLSDHLAYYVNSTLMLDPMDKASVLGDAIKYVKQLRERVQNLEEKTAKITTGSSVLVKRSILFADGENSDSHCANSLPEIEVRVSGKDVLIRTQSDKHSGRAAVILSELEKLHFIVQSSSLLPFGNNNIDVTIIAQMKENYMAAKDLLGRLRKALKQVDGA

>Vun002089-18_Vu

XEAPGLNSCGKSSENALYSPMEQAWQNWSHQMEMDDDDDRHEDLMLEECHTEEEEFLREILGEPSFCSESETHHHNSTTMAPSKRSSSSPRAYILSFDSSTIIPATTPQPPSSSPLSGKKRRQNLNSEQPKPKSTTQKRGRNGSVDHTMAERKRRQELTERFIALSATIPGLKKTDKSSILGEAINYVKELKERVTELEERNKRGKESVMIQKKSDVCDSSETDSKDWCRMLPDIEARVMENEVLIEIHCEKEEGVELKLLDHLENLHLCVTATSVLPFGNSTLGITIIAQMGDAYKMTVKDVVKNLRKVLMNHMNIHGDPY

>Vun004395-18_Vu

MEDEVLNECLCQTNPIDEEFLRDILQQPQEGPDLNTFGGADKLMMKNTNSSMMNLCGENKNKPTTSPTTYVLSFDKSADPHPLSAWKDVSHNLPLSSARTNQGTKKTRSASESMDHIMSERKRRQELTRKFIALASTIPGLKKMDKAHVLREAINYVKQLQERVEVLEEDIQKNGAESAITVTRSHLCIDDTKSDEWYGSNEAVPAEVEARVLGKQVLIKIHCG

>Vun008510-18_Vu

MEDSWQNWLSHLGMNDNFGCTNNGARYEEMVRLNNNMQGCCDSSNNEVASCMEVKEEHSECCWRKRGVGKLRNWREREGRREEQKGQEPLLRLNITSSQREKGDXDIAEKFIALSATIPGLKKIDKASILGEAIKYMRQLQQRIAVLEKGSNSNSVKSFMIAKSHLCSASCEAKSSVEMLPEVEASGLENEVLIRIYCEKRKDMMLNLMTLLKHVHLSVTSSSVLPFGNSLLNIIIVAQMSEEYSLTVTDLVKTLKQNLLKFYEV

>Vun010426-18_Vu

MDCQNEFFPINDDCDDDFFTDLIPPTPFSSESESDHSFRASNTVHNTSFLPGAAVNAAVNAVVASKRSSPRTYILSFDSSTVVPATPEPSVPSSPLPAKRALHTLSPTARPNQGSKRTRTSSQTIDHIMAERKRRQELTERFIALSATIPGLNKTDKASVLRAAIDYVKQLXEKVEELEKQNRKSVAETVIL

>augustus_masked-scaffold05011-abinit-gene-0.2-mRNA-1-19_Cm

MEISSIRGLSELGVEDPRFINQWQMNSLDELGILPIAATFGENLQRSFAHPYFSDKNAMETSHSEIDRPMKQHKANSWSSPSKLDHVPTLQVASSPNILSFANANYMNQMNILKPKEEAACPQSMDTIPTEILTAQGPFGNQNYVYKACQGAKRLSTSTRLSQTQDHIIAERKRREKLSQRFIALSAIVPGLKKMDKASVLGDAIKYLKQLQERVKTLEEQTKKKNMESVVFVRKSQLFTDGDNSTSDKNSSSDPLDEPLPEIEARFCDKNVLIRIHCEKRKGLLEKSVTEIEKLHLTVINSSVMTFGSSALDITIIAQMDEEFCMTVKDLVRNLRSAFKLIM

>maker-scaffold03116-snap-gene-0.21-mRNA-1-19_Cm

MEISSAKWISELEMEDPTFIHQYEINSFGYSLDDLDFNSFSGESYPSNPDLNPKSTYNFHASVIENPHTDLGRPAKQLKTNSWNSCATDHIPSKAASSSSSHFISFENSNSPPAISQQFYGLDCTMKQKSEAASDRNTNFPAMISESSFGMKNCSSKHGQVPKKVASMTRTPLHAQDHVLAERKRREKLSQRFIALSAVVPGLKKMDKASVLGDAIKYLKQLQERVKTLEEQAAKKTMESVVFVKKTRLSAADDTSSSEDNSDSESNEQLPQIEARVLEKDVLIRIHCENRKGYAAKILGEIEKLDLAIVNSSVLPFGNSTLDITVVAQD

>snap_masked-scaffold02766-abinit-gene-0.16-mRNA-1-19_Cm

MEISSAKWLSELGMDEYNYIHQSHMNTLDGFTTHEMATAMVENFQQSLSSESYSSYPTFTKKATTTFSNTSAETSQRPAKQPKTNSWDSYITDHVSPKPSSSSHILSFENSISMLTNPQEFYGSHDSSLKLNNEMVSQANIHFPPLISKDSFENQDYARKASLQGIKRTYSSMTRNPSNAQDHIIAERRRREKLSERFIALSAIVPGLKKMDKASVLGEAINYVKELQERIKLFEEQTKKRTVESVVFVKKSQLSADDDTSSCNENSDNHSDEALLEIEARISEKDVLIRIHCEKQKGAIVKILSEIEKLNLTVVNSSVLPFGNSTIDITIIAQEFI

>WALNUT_00014878-RA-20_Jr

MEDPRFISQWHMNYVDELSIPPLAATFEEESLQQYPFTHPNFNLRNHDMAASRTGIDAPMKMLKPNSWNSSKLEHVSYPQHSPSQNVLSFANANYANQMGILNPKEEAAVCFKRMNTLPSDILISQDSLGNQDYGLKACQGGRRIRRSTRHSQTPDHVIAERKRREKLSQRFIALSALVPGLKKMDKASVLGDAIKYLQQLQERVKTLEEQTKKRNIESAVFVKKSQLFVDGDNSSSGENFSSDPLDEHLPEIEARFCDKNVLIRVHCEKRKGVLEKLIAEIEMLHLTVINSSVITFGSSALDITIIAQMDEGFSMTVKDLVRSLRSAFESIM

>WALNUT_00023299-RA-20_Jr

MELSSGKWLSELEMDDYEYIHQCHMNSTLDDGFPTNDIATAIQENFQQSFSSESYSSYPAFSTKNTITTTTTTLSNSSIETSRNVSFERPAKQTKIDSTWNVSGITQNESPKPSSSPHILSFGNPNSLPDNPKQFCRNLDSSLKPKHEVVTPVNMHVPMISRGSIENQNHAPKASQGNKRAGSVTRSTHSNAQDHIMAERKRREKLSQRFIALSAIVPGLKKMDKASVLGDAIKYVKELQERVNLLEEQTKKRTVESFVSVKKSQLSTDDDTSSCDENFEGRSDEALPEIEARISDKDVLIRIHCEKNRGVVVKILGEIENLHLSVVNSSVVPFGNSTLDITVIAQMDNEFDLTAKDLVKTLRVAL

>WALNUT_00023638-RA-20_Jr

MITRKISNLQGMEDPRFINQWHMNSVDELSLLPLAATFGESLQHSFTHPNFNLKNAMATPQTGTDIPMKQLKSNGWNSYKLEDASYPQLPPTPSVLSFANANYANQMGIVKPKEEAVCSQSMNTLPSDILISQGSLGNQNYVFKAYQGGKRINTSTRLSQTQDHIIAERKRREKLSQRFIALSAIVPGLKKMDKASVLGDAIKYLKQLQERVKTLEEQTTKKNIESVVFVKKSQLFVDGDNSNSGFSSEPLDEHLPEIEARFCDKNVLIRIHCEKRKGVLEKSITEIEKLHLTVINSSVMTFGSSALDITIIAQMDEGFSMTVKDLVTNLRSAFELIL

>WALNUT_00024529-RA-20_Jr

MEISSTKWLSELEMDDFEYIQQCDMNTLDDGFATAMRENFQQSLSSESHSSYPAFNTKTTITTNTFSNSSISQNSFERPAKQTKTNNWNSGIAEHGSIPKPSSSSHILSFWNSNSLPADSKQFFRTLDSSLKPKDEVVCQVNKHVHLISKGSAENQDHGTQKTNSMTRTPSNAQDHIMAERKRREKLSQRFIALSAIVPGLKKMDKASVLGDAITYVKELQARVNLLEEQTKKRTVESFVYVKKAQLSADDDTSSCDENFHGRSDEAALPEIEARISEKDVLIRIHCEKNKGAVVRTLCEIEKLHLSVVNSSVLPFGNSILDMTVIARMDNEFDLTAKDIVKCLRLAV

>WALNUT_00024530-RA-20_Jr

MEISSANWISEVGVEDPIFMHQYDMNSFDYSILDELNFESLSAESYSSYPDLNPKSAHSFNGSAIENPHTGLESPAKQLKTSSWNSCTTDRMTSMAASSSSSKLISFENSNSAPATSQKFLDCTVKPKNEAGSDLRNMNFPTMISESSLEIQNGSSKHEQDPKRVGTVTRTPLHARDHVMAERKRREKLSQRFIALSAIVPGLKKMDKASVLGDAIKYLKQLQERVKILEEQAAMKTTKSVVSVKKARLSADDDISSSDDNSDSHSEHPLPEIEARVSGKNVLIRIHCDKSKGCAAKILSETEKLDLTIISSNVLPFGNSTLNVTVVAQMDDDFCITAKDLVRNLRRALA

>WALNUT_00030451-RA-20_Jr

MEISSAKWISELTVEDPNYLSHQYDLNPFDYSLDGLDHDFQSISAESYSSYPVLKTPKSIHTFNIGVGENIPQTGMDPRPAKLLKTSSWNSFSTDRIASKAPASSSLSQLISFENSNSPPASYQQFYGLDCTMNPKNEAGSDHGNRSFPNLISEKPTSMMQNCSPKQEQFPKRAGSTTRTPLHAQDHVIAERKRREKLSQRFIALSAIVPGLKKMDKASVLGDAIKYLKQLQERVKILEEQTAKKTMETVVFVKKTRISADDDTSTSDENSNSHSDEPLPEVEARISEKNVLIRIYCEKRKGCTAKILSEIEKLHLTITTSSILPFGNSTVDMTVVAQMDVDFCMTAKDLARNLRQALLKLM

>Jcr4S00056.100-21_Jc

MEISSISGLSELEMEDPSFIEQWPMNSIDDLSLLPLVAAFGENYMQHSLINNLKIPMDTSSSTTTIVRPTKQLKPNPLHNLPNPQPSFSPNNILSFANSNQLMGSIVKPKEEAVLCSKSINNTTPPSDMLIPQGSIMNQNYMFKACQGAKRINTNNGRLSQSQDHIIAERKRREKLSQRFIALSAIVPGLKKMDKASVLGDAIKYLKQLQERVKTLEEQTKRKTMESVVIVKKSQLLFCEDDSSSSDESFSKGPFDETLPEIEARICDKHVLIRIHCEIRKGVLEKTIAEVEKLHLNIINSSVLTFGSSALDVTIIAQMDSEYEMSVKDLVKNLHSAFKFFM

>Jcr4S01606.30-21_Jc

MEDPAFNYPYQMNPLDYALDDLDFQSFSSTNDQIFSPQTIQNFNYTLPIQENSQTSIERPTKQLKTNSWNSGSCTTTDNNNSNNQIISSKASPSSSSHIISFENLNNSSPAPISQQFYGLDSTTSTIIKPKSEFVGSNGSANHNSSFFCQAGSTYYPQGNKKTGAMSRTLSHAQDHVIAERKRREKLSQRFIALSAVVPGLKKMDKASVLGDAIKYLKQLQERVKTLEEQSARKNMESVVFVKKCQVYADDESSSTDENCDSCSDQPLPEIEARVSEKDVLIRIHCEKQKGYLLKIFSEVEKLHLNVINSSVLPFGNSTLDITVIAQMDSDFSMTMKDVVRNLRQALLKYM

>Jcr4S11708.10-21_Jc

MEIESARWFSDQDLDDYNLIHEYHMNSLAELTTQNMATALGENLKQSFSSESYTSYHNFNTKNTSTATATIATTTTLSSSSIETSQTSPEKPSKLHKTNSRSSNMITANHQSPKPQILSFETSNPPPFFMTLDHSTVKPKDEAASPRNMHFQSLISKAPQGITNNNKRPYSMTRSPSHAQDHILAERKRREKLSQRFIALSAIVPGLKKMDKASVLGDAIKYVKQLQERVKVLEEQTKKRTVESVVLVKKSQVSTDDDSSSCDENSDGGSDSALPEIEARASDKDVLIRIHCDKQQGILPRILNEVENLRLSITNSTVLPFGNSTLDVTIIAQMDTEFSMAMKDLVKNLRLAFLKFM

>Jcr4S13810.10-21_Jc

MEIESARWFSDQDLDDYNLIHEYHMNSLAELTTQNMATALGENLKQSFSSESYTSYHNFNTKNTSTATATIATTTTLSSSSIETSQTSPEKPSKLHKTNSRSSNMITANHQSPKPQILSFETSNPPPFFMTLDHSTVKPKDEAASPRNMHFQSLISKAPQGITNNNKRPYSMTRSPSHAQDHILAERKRREKLSQRFIALSAIVPGLKKMDKASVLGDAIKYVKQLQERVKVLEEQTKKRTVESVVLVKKSQVSTDDDSSSCDENSDGGSDSALPEIEARASDKDVLIRIHCDKQQGILPRILNEVENLRLSITNSTVLPFGNSTLDVTIIAQMDTEFSMAMKDLVKNLRLAFLKFM

>Manes.07G048600.1.p-22_Me

MFSPMEISSISGLSELEMEDPIFIEQWPMNSIDDLSLQSLVAAFGENMQHSLSHHPNFNLKTSMETSATAIVRPSKQLKPNGFNSIKTTEYNLPNPQADFYPNILSFANSTNLNQMGIVKPKEEAVCSTRINAFPSDMVISPGNQNHVFKACDGAKRISNNSSRYSQTQDHIIAERKRREKLSQRFIALSAIVPGLKKMDKASVLGDAIKYLKQLQERVKTLEEQTKKKTMESVVIVKKSQLLLSEDESFSSDESFSKGPIDEPLPEIEARICDKQVLIRIHCEKRKGVVEKTVAEIEKLHLTVVNSSILTFGSSALDVTIIAQMDMEFVMSVKDLVKNLHSAFKLFI

>Manes.10G090800.1.p-22_Me

MEMEESSFIDQWPMNSIDDPSLPSLVAAFGENMHHSFNLKNSMEAASPNTLAIVRPSKQLKPNGFNSSKTTDYNLQNPEAAFSPNVLYFANSTNPYQMGFVKPKEEAVCSRNFDAFTSDMLVSPENQNFMFKACQGAQRFSSNNSSRHSQTQDHTIAERKRREKLSQRFIALSAIVPGLKKMDKASVLGDAIKYLKQLQERVKTLEEETKEKIIESVVIVKKSHLLFCEDESSFSDESLSKVFVNKPLPEIEARICDKQVLIRIHCEKKKGVLKKTVAEIEKLHLTVVNSSVLTFGSFALDVTIIAQSGKNENLNWEQKMEDEFGMSVKDLVKNLHSSFKLFM

>Manes.12G119700.1.p-22_Me

MDIASARWFTEQDLDDYNLIHEYHIKSLADQLTTQNMATAPEENLRRSFPSESCSSYPPLSSSSIETCQTGSERPSKLHKTSSFNSSMITTEHPSPKPSTTQVLCFESSLISAPANSQQCFMNLNSATTFKPKEEAASPRNLRFQPLISKVAPFESQNYEMKASQGASNNKKPYSISRTPSHAQEHILAERKRREKLSQRFIALSAIVPGLKKMDKASVLGDAISYVKQLQERVKVLEEQTKTRTVESVVLVKKSQLSADDDSSSSEENSDGSSDSALPAIEARVSDKDVLIRIHCDKQRGVVPKILNDVENLNLSIINSSVLPFGDSTLDITIVAQMEAEFSKAAKDLVKNLRVAFLKLT

>Manes.13G105700.1.p-22_Me

MDIAPARWFTEQDLEDYNLIHEYHMNYLDELTNQNMAAALGENLRQSFSSESYSSYPPFKAKDTATATANTTTLSSSSIENSQTSSERPSKLHETNSWNSSMITTDHQSPRPSTTPQILSFESSSVSAPANSQQFFMTLDSTTVKPKDEAASPRNMHFQPLISKVAPFGNQNHEIKTRQGTTNKRPYSMTRTPAHAQDHILAERKRREKLSQRFIALSAIVPGLKKMDKASVLGDAIKHVKQLQERVKVLEEQTKKRTVESVVLVKKSQVSADDDSSSCDENSDGGSDSALPEIEARVSDKDVLIRIHCGKQQGVVPKILNEVENLNLSIINSSILPFGNSTLDITIIAQMEAETSMAVKDLVKNLRVAFLKFM

>Manes.13G105800.1.p-22_Me

MEISSAKWLSELGMEDSAFSYQYQMNPLDYPFEDLDFHGFSAKSYSTNEQIFNPQTVQNFSCAPIESSQTSYERPKKQLKTSSWNSCTTEQINSKPPPSSSHIISFENSNSSAATSQQLYGLDPTSVKPKKEAGTNGNSNYPSALCCQGSFEEYGQGNNKKAGTLSRSPLHAQDHVLAERKRREKLSQRFISLSAVVPGLKKMDKASVLGDAIKYLKHLQERVKTLEEQAAKKTMESVVFVKKSQVYVDDDLSSTDENSVGCCDQPLPEIEVRVSDKDLLIRIHCENQKGCLLKILSELEKFHLNVINSSVLPFGNSTLDVTVVAQMDGDFSMTVKDLVRNLRQALL

>28179.m000478-23_Rc

MVTALGDELQPSFSSDSNSSYPVFTAKSTATAAAAGTTLSSYFIETSQTSYERPSKLHKTNSWNSDITTEDQSAKPSSTSQLLSFETPKVSSPVNSQRFYMKPKDEAASPRNMHFQPVISRPPYDIQNHDIKTIQGITNNKRPYSVTRTASHAQDHILAERKRREKLSQRFIALSALVPGLKKMDKASVLGDAIKHVKQLQERVKMLEDQTKKRTMESIILIKKSQLSADDESSSCDDNSDGCSDSALPEIEARVSDKDVLFRIHCEKQQGVVPKILHEVENLHLSIINNTVLPFGSSTLDITIIAQMDENNSMAVKDLVKNLRVALLKFM

>28179.m000479-23_Rc

MEIPSAKWLSELGMEDASFNYQYQINTLDYSIDDVDFQSPSSESYSLNDQIFNPQSVQNMSCAPSNIDQRPAKQLKTNSWRSCTTDQITSSKASPSSSSHIISFDNTNSSPATSPHFYGLDSTTVKPKTEIGPNGKVNHDPSSLFGLGSFEDQYGSTYYNQGTKKAGASTRSPLHAQDHVIAERKRREKLSQRFIALSAVVPGLKKMDKASVLGDAIKYLKHLQERVKTLEEQAAKKTMESVVFVKKSQVYADDDSSSIDENFVGSCDHPLPEIEARVQTRISSLKSIVKSRKVAYSKY

>29804.m001498-23_Rc

MAAFGENMQHSLSHQNFNLKASMETSPLSGINLRPTKQQLKPNENYLHSSFSPNILSFANSANTNHPMGLVVKPKEEAAVYYKSIHTLPSEHMLISQGNSLENHNYVFNACQGAKRISTNNNNGRISQSQDHIIAERKRREKLSQRFIALSAIVPGLKKMDKASVLGDAIKYLKQLQERVKTLEEQTKKKTMESVVIVKKSRLVFGEEDTSSSDESFSKGPFDEPLPEIEARICDKHVLIRIHCEKRKGVLEKTIAEIEKLHLSVTNSSVLTFGSSALDVTIIAQMDNEFSMSVKDLVKDLHSAFKLFM

>Lus10003488-24_Lu

MEDYKFFQHCDTINSEFEQNTVAAAALLPGEALQPSFSSESYSSYSAGFHHQSTAPNTTIFTPPPAAISRSSINETSQTKTFDDHRPNKQQRKTNSWSSSVTTDHQQHIDFSSNPHPQMLSFDNSKSTTSSLQPLYKNGLELTVKPKEEADMLFHSTTNTRMMSDPSENTIYAPMGMKTKPFSSLTRTPSHAQDHIMAERKRREKLSQRFIALSSIVPGLKKMDKASVLGDAIKYVKHLQEKIKQLEEQTKKRTVESVVLVKRCQLPMDEDSNSSSCEDDNNNSSKGEKKGCWDSTLPEIEAKASERDVMIRIHCEKQPGVVAKLLYEVESLNLSIVNSSVLPFGNSTVDITVIAQMDGEISMTVKEVVKNLRLAFLKFM

>Lus10011578-24_Lu

MEVLQTEWMPELSSDMNNNSRPAKIPKTTNNSSRASPPSSSSSPYSCIISFDSSSSTDSVPAVPQKPPRSYPPAAVIKPEYSSIENGLQYGCSVLNNATTSHGTKRPAAAMSRSPISAQDHVMAERKRREKLSERFIALSAVVPGLKKTDKASVLGDAIKYLKQLQERAKTLEEQVKRKTIESVVYVKKSRVYNLDDDELSSSTDENSDGFSSNQSLQLPEIEARVSGKDVMIRIHCEKQKGCLPKLLCEIEKLGLNVMNSNVLPFGSSTLDITVVAQMDHDFSTTTIDLVRNLRKALK

>Lus10012457-24_Lu

MEDHSHFINQWRQMESSNNSSFGVPTLENGLFNTTMPSHHHALEMGFLKPKQETAENMYLSQQNWFGNHHNIISKGRIRMSQQSQDHVIAERKRREKLSQKFIALSAIVPGLKKMDKASVLGDAIKYLKQLQERVKTLEEQTRNKSMESAKVVVKKSQVFDECGSSEQLPEIEARFSDRHVLIRVHCEKKKGLLEKTVAEIEKLHLTVINTSVLTFGSSALDLTIITQMDVEFNMTVKEVVQKLHPAFSLFN

>Lus10019247-24_Lu

MEDPSFNYQYQSPFNFSIDDFDFDHHSFTPANCSMRFPNEEAAVNNPPLIISPQSFTTTSQSSDMNNNSRPAKIPKTTSDSRASPPSSSSSPYSCIISFDSSSSTDSVPAVPQKPPRSSYPPAAVKPEYASIENGLQYGCSVLNNAASHGTKRPAAAMSRSPISAQDHVMAERNRREKLSERFIALSAVIPGLKKTDKASVLGDAIKYLKQLQERAKTLEEQVKNKTIESVVYVKKSRVYNLDDDELSSSTVENSDGFSSNQSLQLPEIEARVSGKDVMIRIHCEKQRGCLPKLLCEIEKLHLNVMNSNVLPFGSSTLDITVIAQMDHDFSTTTVDLVRNLRKALT

>Lus10020503-24_Lu

MEDHTHFINQWRQMDSSNNSSFGVPTLENGLFNTTMPSHHHALEMGFLKPKQEAAEMYLSQQNWFGNHHNVISKGRTRMSQQSRDHVIAERKRREKLSQKFIALSAIVPGLKKMDKASVLGDAIKYLKQLQERVKILEEQTRNKSMESAKVVVKKSRVFDVGYDECGSGEQLPEIEARFCDRHVLIRVHCEKKKGLLEKTVAEIEKLHLTVINTSVLTFGSSALDLTIIAQMDMEFNMTVKEVVQKLHPAFNLFK

>Lus10039639-24_Lu

MEDYKFFQHCDTINSEFEQNTVAAAALLPGEALQPSFSSESYSSYSAGFHRQSTAPNTLTPPPAAISRSSINETSQTKTFDDHRPKRQQRKTNSWTSSVTTEHQQHIDFSSNPNPQMFFRQLQIHHFLSSAFLQERLRANKPLPTLKITSWLSSSLTRTPYHAQDHIMAERKRREKLSQRFIALSSIVPGLKKMDKASVLGDAIKYVKHLQEKIKQLEEQTKKRTVESVVLVKRCQLPMDEDSNSSSCEDDNNNSSKGEKKGCWDSTLPEIEAKASERDVMIRIHCEKQPGVVAKLLYEVESLNLSILNSSVLPFGNTTVDITVIAQLCIVAYEEKD

>Lus10039640-24_Lu

MEDPTMTWEGEYHQIIQNPFGYPHQDDFSFEHYPLMHDRCLMNGRNETNSAYNNPYTSVVDSIASLGIRSPTTTTTTSAATHHHISTEFNGAETHLHSKPSHPPPISSSSSSSSSNCMISFGNPPSPNTTSHEQQLCYYDCLNSETSNNDGSVGGFDGRRPISNRRPPHGQDHVIAERKRREKLSQHFIALSALVPGLKKTDKASVLGDATKYIKHLQERVKSLEKQTRCSHNKTVEMVHYYVNRSQVLCMEDAACNSPSTNEENWKGRILCSNNNDDINNPSSSSCWSRGTLPEIEARVCDEEVMIRIHCERQKGSSSLAKLMSEIEKHQLNVVNTSVLPFGTSLLDITVLAHHHHLQGMQMESGYMSSRMRHLVKTLQRVIVQ

>CCG003376.1-25_Pe

METSPAAWLSDEMGMEEYSAFGHVRYDTNPLDYSIDEFNFQTFPSKCFSMNDQTFNHQAPQNFTCASSISSQVSIDQRPAKQPKNSQQAPPSSSSHVISFDNSSPPPASSLQYFGSTNYEDGATYFRKAGTKKIATTSKSPSHAIEERNRREKLSQRFIALSAVVPGLKKMDKASVLGDAIKYLKYLQERVKTLEEQAAKKTMESAVFVKKSMVCIADDSSSSTDENPAGGCRDYPLPEIEITVSDEDVLIRILCENQKGCLTKILTEMEKLHLKVINSVVMPFGNYTLDVTIVAQMDVDFSMTLKDLVKNLRRALLQG

>CCG017618.1-25_Pe

METSSITALYELGMEDPGFTNQWYMNSLDDISLLPLAAAAFGENVHHPFSHQNFNLKTPMDSNPTSINVRPPKQMKTFHLSDPQSAFSPNFLSFVNPNHANQMGLVKPKEEAVCSQSINNFPSDMVVSQDIFGSQNYVFKACQGPERISTNTTRLSQSQDHIIAERKRREKLSQRFIALSAVVPGLKKMDKASVLGDAIKYLKQLQEKVKTLEEQTKRKTMESVVIVKKSHIYVDEGDVNSSSDESKGPIHETLPEIEARFCDKHVLIRIHCEKRKGVLEKTVAEIEKLHLSVINSSVLAFGTSALHVTFIAQMDIDFNLSLKDLVKTLRSAFEFFM

>CCG019679.1-25_Pe

METSSITALCELGMEDSSFTNQWFMNSLDDTGLLPFAAAFGENMHHSFSHQNFNLKTSMDSARPTKQLRTDHLSNPQPAFSPNILSFVNSNHANQMGLMKPKEEAVCSKSINNLPSDMVVSQDSFGNQNYAFKASQGPKMISANGTRLSQSQDHIIAERKRREKLSQRFIALSAVVPGLKKMDKASVLGDAIKYLKQLQERVKTLEEQTKRKTMESVVIVKKSRVYVDEGGENSSSDVSKGPIHETLPELEARFCDKHVLIRIHCKKNKGVLEKTVAEVEKLHLSVINSSVLTFGTSVLDVTIIAQASSVLNHSF

>CCG033096.1-25_Pe

MPDPIESYKPYTLTQTLQGHKSSISSVKFSSDGRLLGSSSADKTIKTYSLSPSNPPTSPITPLHDFHGHEQGVSDLAFSSDSRFIVSASDDKTLRLWDVTTGSTIKTLHGHTNYVFCVSFNPNSSMIVSGSFDETVRIWDVKSGKCLKVLPAHSDPVTCVDFNRDGSLIVSSSYDGLCRIWDSGTGHCIKTLIDDENPPVSFVKFSPNGNYILVGTLDNNLGMDDYKFIHQCHINSLAEFTAQNMATTLLGENLQRSFSSESFSSKPSLMMTRNTTITSTSNGSSSETSQTSIETPGKQQRTNSWNSSFSTLHQSPKPTSSFSTPHQSPKPPSPIPESFSFNTSAPPPTASSQQFYGNLDRLIKPKDEAASPINMHFQTSISKAACERSESYAPEAKQGIKRPYSMTRSAMHVQDHIMAERKRRKKLSQQFIALSAVVPGLKKMDKASVLEGAMKYMKQLQEQLKQLQDQTKTKTMESVVLLKKSKLSVDDECSSSDENFDGLPDSPLPEIEARTTDKDVLIRIHCKNQQGVGIKILSEIENLHLSVVNSSVLVFGNSTLDVTVIAQMDNDFSLTMKDLVKKLRLACMKLSCAIIPSSCIQA

>Potri.001G287200.1-26_Pt

METSSVTALCELGMEDSSFTNQWFMNSLDDTSLLPFAAAFGENIHHSFSHQNFNLKTSMDSVRPTKQLRTDHLSNPQPAFSPNILSFVNSNHANQMGLMKPKEEAVCSKSINNLPSDMVVSQDSFGNQYYAFKASQGPKMISANGTRLSQSQDHIIAERKRREKLSQRFIALSAVVPGLKKMDKASVLGDAIKYLKQLQERVKTLEEQTKRKTMESVVIVKKSHVYVDEGGENSSSDVSKGPIHETLPELEARFCDKHVLIRIHCKKNKGVLEKTVAEVEKLHLSVINSSVLTFGTCALDVTIIAQMDIDFNMSVKDLVKTLRSAFQYFM

>Potri.005G095400.1-26_Pt

MTKLSIIKPHKTLPVHPLFPLKSALIRGQQNSQKLLNRLRLLPPGTKKIAATSKSPSHAIEERNRREKLSQRFIALSAVVPGLKKMDKASVLGDAIKYLKYLQERVKTLEEQAAKKTMESVVFVKKSLVCIADDSSSSTDENSAGGCRDYPLPEIEITVSDEDVLIRILCENQKGCLMKILTEMEKLHLKVINSIVMPFGNYTLDVTIVAQMDVDFSMTLKDLVKNLRRALM

>Potri.007G009400.1-26_Pt

MDDYKFIHQCNINSLAEFTAQNMATTLLGENLQRSFSSESFSSKPSLMMTRNTTITSTSNGSSSETSQTSIETPGKQQRTNSWNSSISTLHQSPKPPSPIPESFSFNNSAPPPTASSQQFYGNLGRLIKSKDEAASPINMHFQTSISKAACERSESYAPEAKQGIKRPYSMTRSAMHVQDHIMAERKRREKLSQQFIALSALVPGLKKMDKASVLDGAMKYMKQLQEQLKQLQDQTKTKTMESVVLLKKSKLSVDDECTSSDENFDGLPGSPLPEIEARTTDKDVLIRIHCKNQQGVGIKILSEIENLHLSVVNSSVLVFGNSTLDVTVIAQMDNDFSLTMKDLVKKLRLACLKLSCAIIPSSCVQA

>Potri.009G081400.1-26_Pt

METSSITALYELGMEDPGFTNQWYMNSLDDISLLPLAAAAFGENVHHPFSNQNFNLKTSMDSTPTSINVRPTKQMKTFHLSDPQSAFSPNFLSFVNPNHANQMGLVKPKEEAVCSKSINNFPSDMVVSQDIFGSQNYVIKGCQGPERISTNTPRLSQSQDHIIAERKRREKLSQRFIALSAVVPGLKKMDKASVLGDAIKYLKQLQEKVKTLEEQTKRKTMESVVIVKKSHIYVDEGDVNASSDESKGPIHETLPEIEARFCDKHVLIRIHCEKRKGVLEKTVAEIEKLHLSVINSSVLAFGTSALHVTFIAQMDIDFNMSLKDLVKTLRSAFEFFM

>SapurV1A.0019s0910.1.p-27_Sp

METSSITALCELGMEDPSFINQWYMNSLDDISLLPLAAAFGENAHHPFSHQNFNLKTSMDAAPTSINVRPTKQMKTYHLSDPQSAFSPNILSFVNPNHANQMGLVKPKEEAVRSKSINNLPSDMVVSQDIFGNQNYVFKACQGPERISTNSTRLSQSQDHIIAERKRREKLSQRFIALSAVVPGLKKMDKASVLGDAIKYLKQLQEKVKTLEEQTRRKTMESVVIVKKSHIYVDEGDGNSSSDESKGPIHETLPEIEARFCDKHVLIRIHCEKRKGVLEKTVAEIEKLHLSVINSSVLAFGTSALHVTIIAQMDIDFGMSVKDLVKNLRSTFEIFM

>SapurV1A.0279s0180.1.p-27_Sp

MDTTPATISTVRPTKQLKTDHFPNPQSAFPPNILSFFNSNHANHMGLMKPKEEAVCSKSISNLPSDMVVSQDSFGNQNYVFKSSQGPKRINTNGTRLSQSQDHIIAERKRREKLSQRFIALSAVVPGLKKMDKASVLGDAIKYLKQLQDKVHTLEEQTKRKTMESVIIVKKSHIYADDGDGNSSSDLSKGPVIIHETALPELEARFCDKHVLIRIHCKKNKGVLEKTVAEVEKLHLSVTNSSVLAFGTSALDVTIIAQMDVDFSMSVRDLVKTLRSAFQYFM

>SapurV1A.0821s0050.1.p-27_Sp

MDIAAWKWLSEMGMDDYRFIHQCHINSLAEFTTQNVANTLPGENLQRSFSSESFSSKPSLMMARNTTITGTSNGSSSETSQASIETPGKQQKTNSWNSNIATLHQSPKPPSPISESFSFNTPAPPPTTSSQQFHRNLDRMIKPNDEAASPINMHIQTSISKAACERSESYAPEAKQGIKRNYAMTRTALRVQDHIMAERKRREKLSQQFIALSAIVPGLKKMDKASVLEDAMKYMKQLQEQLKELQDQTRTRTMESVVLLKKSKLSIDGECSSSDENFDDRSSSLLPEIEARTTDKDVLIRIHCRNQQGVGMKILSEIENLHLSVLNSSVLVFGNSTLDITVIAQMDDEFSLTMKDVVKNLRSACLKLYQARALKPEDSP

>SapurV1A.3034s0010.1.p-27_Sp

METSSITSLSELGMEDSSFTNQWYNMNSLDDTSLLPFSAAFGDNMHDQYSFSHQNFNLKTSMDTTPATISTVRPTKQLKTDHFPNPQSAFPPNILSFFNSNHANHMGLMKPKEEAVCSKSISNLPSDMVVSQDSFGNQNYVFKSSQGPKRINTNGTRLSQSQDHIIAERKRREKLSQRFIALSAVVPGLKKMDKASVLGDAIKYLKQLQDKVHTLEEQTKRKTMESVIIVKKSHIYADDGDGNSSSDLSKGPVIIHETALPELEARFCDKHVLIRIHCKKNKGVLEKTVAEVEKLHLSVTNSSVLAFGTSALDVTIIAQMDVDFSMSVRDLVKTLRSAFQYFM

>PK09122.1-28_Cs

XDGLKNNNTTSNFLPSDCVVKVANHQGANCNKRLINSTSTKLSQTKDHILAERKRREKLSQRFIALSAIVPGLKKMDKASVLGDAIKYMKQLQERVKILEEEMRKRNMESVVFVRKFQLIADKDSNSPSYSSDENYSSFDEPLPEIEARVCDKNVLIRIHCEKKKGVMEKTIVEIEKHHLTIINTSCMTFGTSSLDLTIIAQMNMEFSLTMKDLVKNLRLAFSLFK

>PK11573.1-28_Cs

XQGVEDYNNIFSLDNQLEFGSDDHQILVGGDNYKQDFSSESNSSYSTITTSGGSDSIKKYSHHDHHQASNKKPAPSSNSSQLLSFDNYQNQHYPNSSSPPKINMSDISSTTLRPKKGLSQTDINFSSLDDVDHVVSNNKDRNSFIISNKKKNYSSSYQPSIFIKGTNDYNNNNKRPYSMIKTPSHAQDHIMAERKRREKLTQRFIALSAIVPGLKKMDKASVLGDAIVYVKQLQERVNILEEQSKSRTVESVVFKKSQVSISSSTDEQNQDSSSSDQNFEGRSNNDQDYNHNNNNDNNQTLTLLPEIEARVYEKDVLIRIHCENHKGIVVKILSEIEKLDHHLSVVNTSVMPFGNCTLDITILAQMEKEFSMTVKDLVKNLRKALLKIM

>HL.SW.v1.0.G000440.1-29_Hl

MDVASANWLSEVLGEEDYSSIFHHQYEINSLNQLGIASDDHIIGDNYKQFFSSESYSSYSTITTKTNDPSITTSSGSDSIKNSHEALNKKLSLSFSSSQLLSFDQKQFSNPPPNNSNLHHHDQKVVYGNLSMSNNTTLRPKREAVPEIDMNFFNKDSLITKKKDNYSSCEPTTFNKGTNKRPYSMIKTPSHAQDHIMAERKRREKLTQRFIALSAIVPGLKKMDKASVLGDAIMYVKQLQERVSVLEEQTKSRTVESVVFKKSQVSADDHNHQGSSSSDQNFEGRSHHDQDDHNQTLPEVEARVSEKDVLIRIHCEKQKGIVVKILTEIEILDHLSVVNTSVLPFGNCTLDITILAQVEKEFSMTMKDLVKNLRKALLKIM

>HL.SW.v1.0.G017572.1-29_Hl

MEILSAKSLSDELEMNDALSNFMCDQYDMDCLDLIDLEDDGDHHHHEQQLNYFQSSSPDEGNIHTPTICSNYQQNTTTKSSLPVDKERVKFKSSRNWNSRTSAAPIAKMVSTTTTSSSAALISFNNSRSNSTATATALVPSSEKNYYLEYWGSTTTATTSDQFYVPKQTQNCTQKCNNKQGVRRVGSISTNTRSPLHARDHVIAERKRREKLNQKFIALSAVVPGLKKMDRTSVLGDAINYVKHLEERVKTLEEQQEMKTENHKPVILMKKSLLCTVEDSLSDNDMVMQPLPEIEARVSNKDVLIRIYCEKQYGGCNLANLLTKIENLHLTIVNTSALPLGSSTIDITIVAQ

>HL.SW.v1.0.G033561.1-29_Hl

MEISSAKWMSELELEDPANIFEQYQVDYSFDDLNFQSFSSESYSSYPNFVPENPQNLSSNNTILDQAPQTESFERPSKQLKTNSWNSCATTTPNDQISTKASSSSSSQFISFEKYANSASATPENKKYYNDLDVTTTTITTTLNVKPKDEPGFNGYNMVFQSPYETRNCSPNKYGQGVKRPLGVSTTRSPLHAQDHVMAERKRREKLSQRFIALSAVVPGLKKMDKASVLGDAIKYIKNLQERLSTLEERSAKKTVESVVFIKRSHLSADDETSSSDENFDSNSNQPLPEIEAKVSDKDVLIRIHCEKHKGCLANILSQVEKLNLTIFNSSVLPFGGSTIHITIVAKMDVEYNMKVKDLVRNIRQALLRN

>HL.SW.v1.0.G038215.1-29_Hl

MESNFSSSMDKASVLGDAIKYMKQLQERVKTLEEEMRKRNMESVVFVKKFQLFADKDNNSSYSSDENCSSFDEPLPEIEARVCDKNVLIRIHCEKKKGVMEKTIAEIEKHHLTIINSSCMTFGSSSVDLTIIAQMNMEFSITMKDLVKNLRLAFSLFM

>HL.SW.v1.0.G038216.1-29_Hl

MDITSVKSLSELGMNDPNMINRHWHMNSLDEFSNSMVLPIAASLGENLHHLHPHPHSNSNFSLVKASSFDNNSHMGIDYRGMMKHHKPNGWNSNEIISHLSNNPPQQVASSPNLYSFAAGSNNYVIDEMGYVRPKEEAVDGLKNINTTSAFPSDYVLKTNQVANKRFNNTSTKLSQTKDHILAERKRREKLSQRFIALSAIVPGLKKV

>XP_010105102.1-30_Mn

MEIPSAKWLSELEMDYNFFHQYDQVNSLDHHYSFDDINFQSFSSESYSSHNTNFAPANSQNLGGAATPVDQAPHHQITDFESRPAKQLKINNSWNNNNSCTTNDNYHYHKTSAKAASSSSSQIISFEKYASSAATTPEKYYDNLDQSPVKQPKDEPAGSTDKYMIFQSSYHDRNENFSPKLGQVIREKRPAAAMSRSPLHAQDHVIAERRRREKLNQRYIALSAVVPGLKKMDKASVLGDAITYIKTLQERVSILEEQAAKKTVESVVFVKRSHLSADDEISSSDENFDSSSDQPLPEIEARVSGKDVLIRIHCEKQKGCLSNILCEIEKLHLTIVNSSVLPFGGSTTHITIVAQMDVEYSMNAKDLVRNIRQALLKNYT

>XP_010105103.1-30_Mn

MDVASAKWLSELGLDQDYSSSFFHKSEMNSLNQFGFISEEVATALGEDYKQSFSSESYSSYSTITTKNPSNAASSGSDYSVKDSQVSFDRPPKQLKISGSPSSQLILSFENSNSSPTRPQRHVYGNFESTLRPKKEAASQIDHVPYFSSVVTKDHSVLNGDNIDSAPKTSIKGTKRSFSSMTRTPSHAQEHIMAERRRREKLSQRFIALSAILPGLKKMDKASVLGDAIKYVKELQERMKTLEEQNKKRTVESVVFVKKSQLMISSTDDDTSSSNDSHDSHSDEVLLPEIEARVSEKDVLIRIHCEKQKGFMVKMLSEIEKLHLSIVNTSVLPFGNSTFDITVIAQDEPLYKELAIKLMENEFNMTVKDLVKNLRQALLKFM

>XP_010107640.1-30_Mn

MQGMEDSSYINQWQMNSLDELSMLPLATTLGENLHHHYHHHPSFNIKASLENSHIGMKHHKVNGWNSSESINNLSNPQVLASSPNLFSFVSSNYNTNQMGLVKPKEEAIRLKNINTVTVPSDVLVSQGCFPNQNYVLKANQGAKRISTSTKLSQAKDHILAERKRREKLSQRFIALSAIVPGLKKMDKASVLGDAIKYMKQLEEKVKTLEEQMRKKNMESVVFVKKSRIFAVEDNSSSDENVSGGSFSKPLPEIEARVCDKNVLIRIHCEKRKGILEKTIAEVEKFHLTVINTSCMTFGSFSLDVTIIAQMNVEFSLTVKDLVKNLRLAFTLFMM

>XP_015869190.1-31_Zj

MQSPHGPQTVIERPAKQLKITLDSSSSSHIISFQSSNESTVKPKESVGYNSNGNGNMDFPSLISYQDSYKQMQNLSTNYGQGVKRPVNDSAMRLSRTPSLAKDHVIAERKRREKLTQRFIALSAILPGLKKMDKASVLGDALKYVKQLQERLKTLEEQATKKTVQSVVLVKRFHVSDEGDISSSDHNHNEVVNSCYDQLLPEVEARVLGKDVLIRIHSEKLDQGCLVKILNEIEKLYLIIVNSSVLQFANSTHITIVAQMEIEFCMKAKELARNLRHAFLNFI

>XP_015869191.1-31_Zj

MEIPLAKWLSAAELEVKDSTFLNQYQMDNLHNSDDDLNFQSYTSEGHSYYPKNDHNLVNFPFEIPQTGTESPPNKPLKSNSWSSCTTEYTSTKASSSSSPHLISFQNSSSPPPPPATAEQYHGLDCTIKPKNELDYEESMILIPPFLSDLDHSFQTQKYYCLQKFGHSVKRPFGAMSRSPLHAQDHVIAERKRREKLTQRFIALSAVLPGLKKMDKASVLGDAIRFVKQLQERVKTLEEQAAKSAGESRIIVKRTKVLVDDHHHEISSSLDESCNDKPLPEVEAKVSGKDVLIRIYCEKHKGCLVNILREIEKLHFTVVDSSVLQFGNSTLDITIVAQMDVEFCMKVKDLVSYLRQALLQFI

>XP_015870213.1-31_Zj

MNMATGLENQDGQPENLKKQLALAVRRIQWSYSIFWSISASQPGVLEWSDGYYNGDIKTRKTVQAVELNADQMGLQRSEQLRELYESLSAGEASPQSRRPSAALSPEDLSDAEWYYLVCMSFVFNIGQGLPGRTLANDKPIWLCNAHFADSKVFSRSLLAKSASIQTVVCFPFLGGVVELGVTELVMEDPDIIQQIKTSILEIPYPIVSRNANLCAANARNDLDHDPVETKSVPVLGSDELDMASPNDNSDGFEPIQLADDSLIIEGINGGASQVQSWQFMDDDELSNCIPHSMDSSDCISQTLLNPEKPASGPKSEMVGDHQLQDLQDCNHTRLSALDLHSNDVHYQGVLSALLKSSHQLLLGPLFQNSHQESSFISWKKGGFVKCPKPRGEISQNLVKKVLFEVPRMHAACRLESPEDNGNRDGVWRPEADEIGMNHALSERRRREKLNKRFSILKSLVPSISKDDKISILDDAIEYLKVLERKVEELEYFRESTEIEPKTKRQPQDTTERTSDNYGNNKTSSGKKPLINKRKASDIDESEQGINYGVQDNGLADNVTVSMKNNSVTIEIMCSWREGVLLEIMDALSNLHLDSHSVQSSIIDGILSLTIKSKFKGS

>XP_015871249.1-31_Zj

MNMATGLENQDGQPENLKKQLALAVRRIQWSYSIFWSISASQPGVLEWSDGYYNGDIKTRKTVQAVELNADQMGLQRSEQLRELYESLSAGEASPQSRRPSAALSPEDLSDAEWYYLVCMSFVFNIGQGLPGRTLANDKPIWLCNAHFADSKVFSRSLLAKSASIQTVVCFPFLGGVVELGVTELVMEDPDIIQQIKTSILEIPYPIVSRNANLCAANARNDLDHDPVETKSVPVLGSDELDMASPNDNSDGFELNQLADDSLIIEGINGGASQVQSWQFMDDDELSNCIPHSMDSSDCISQTLLNPEKPASGPKSEMVGDHQLQDLQDCNHTRLSALDLHSNDVHYQGVLSALLKSSHQLLLGPLFQNSHQESSFISWKKGGFVKCPKPRGEISQNLVKKVLFEVPRMHAACRLESPEDNGNRDGVWRPEADEIGMNHALSERRRREKLNKRFSILKSLVPSISKDDKISILDDAIEYLKVLERKVEELEYFRESTEIEPKTKRQPQDTTERTSDNYGNNKTSSGKKPLINKRKASDIDESEQGINYGVQEDGLADNVTVSMKNNSVTIEIMCSWREGVLLEIMDALSNLHLDSHSVQSSNIDGILSLTIKSKFKGSTLASARKIKQALQRFVGSC

>XP_015890378.1-31_Zj

MDDFYLPDEDELGTELMVAAFVEDLHQTISNSESNTSYSTPPLVSQSINNDNTTTSYTSMEFHQRPPTNQLNLNNNDQTSSVLDYKTNSEPLILSFGNSDLFNNVPDHHLHYKENIAIAGFSGDLSSSSPVFNYSNINSSNANQFHEDEIIFREWSKKISGGTTKPASQLRDHVLAERKRRENLNKLFIALSTVVPGLKKTDKSSVLGETIKYIKQLQEKEKKLEVQSAKKDIEQVVVVKKTKIIVDDEDENDCSNETSSDSKEEWLPQIEAKVSGNNVLLKLHCKKQKGVLAKALSEMDKYNLKVINTSAMPFGDLCLDITIIAQMEQECCAPLIKSLVRCLRAAFEQL

>XP_015890379.1-31_Zj

MVFRIAIQQGTKRSYSMSRTPSHAQEHIIAERKRREKLSQRFIALSGIVPGLKKMDKASVLGDTIKYVKQLQERVKVLEEQTKKRTVESVVFVKKSQFSGDDDSSSCDENFDGRSDEALPEIEVKVSEKDVLIRIHCEKQRGVTVKILSEIEKLQLSVVNSSILPFGNSTLDITIVAQMDDEFSMTVKDLVKILRMALLKFM

>XP_015890448.1-31_Zj

MEIASAKWLSEFEMEDPTFINQYNSLDYSLDGLNFQSFSSESYSSYPNFNTPKCAPHQLLSTTPMETPHHQTTSIERPSKQLKSNSWNSCTTTHDHQISTKASSSSSSHLISFENNSAEKAVMPPTSEQYYASHGPIKPKNEVGSDGNMSVFPSLISRTSYETQNHSMKHTEGVKSSSTSAGTMSRRTAIHAQDHVLAERKRREKLSQRFIALSAVVPGLKKMDKASVLGDAIKYVKQLQERVNTLEEQAAKKTVESAVFVKRSLVSGDDELSSSDENFDSCSDQPLPEIEARVSDKDVLIRIHCEKHKGCLSNILSEVEKLPLTIVNSSVLPFGGSTLDITIVAQMDVEFSMNVKDIVRNLRQALLNFI

>XP_015893295.1-31_Zj

MDIRGLPEMGMEDPSFINPWYMNSLDEISMLPLAAALGENLQQSHSHLYSNFNLKASMDGSHIGTVIDRPVKHHKPNSWNSTKVDPISNPLVAYSPNFLSFVSSNYTNQMGLMKPKEEAVCLKGSKNPPSDGLVSQGSYANQDSALKANQGAKRVCSSTRLPQTQDHIVAERKRREKLSQRFIALSAIVPGLKKMDKASVLGDAIKYMKHLQEKVKTLEEQTRKENMESVVFVKKTQLFADGDNSSLDKNFCSGPFNEPLPEIEARFCDKNVLIRIHCEKRKGILEKTIAEMEKLHLTVTNSSCLTFGSSVLDVTIIAKMNEEFSLTVQDLMLFSAKFYH

>mrna05610.1-v1.0-hybrid-32_Fv

MEDPTFLNQYEMNFHLDYSLEDLNFRSLSAESYSSYPDFTPPQNDVDERPAKQPKNNHSNWNSCTTTHDHKTTVPRASSSASSHLISFDNSNSSAYGSLDCTTTVKPKNEVQGSGGNLNSIPTLISQGNSYDPQTCSPNRAYGQGIKRAATVTRSPLHAQDHVLAERKRREKLSQRFIALSALVPGLKKMDKASVLGDAIKYVKQLQERMKILEEQAAKKTVESVVFVKRTQYSADDDISSSDENFDSCSDQPLPEIEARVSDKEVLIRIHCEKKKGCLANILHEIERLNLTILNSSFLPFGNSTLDITVVSQMDVEYSMTVKELVRSKALYTSLSCTNAEKTTLPAEQWYTAMAGFLLQGFVYVGGGFFPSFLRG

>mrna05612.1-v1.0-hybrid-32_Fv

MDASSAKWFSDLGMDDYNFIQEGFTAQDIATALGGNFKQSFSNESYSSYSTLTTQNTPNTTLSGGSSINETNSQTSFERPAKLVKTNNWNSGITEHVSPKPSSNSSSSHILSFENANLNSSPPSKPHQKFSNGFDTLKPKEEAPSQICMQFENQGYGTNKRPYSMTRTPSHAQDHIMAERKRREKLSQRFIALSAIVPGLKKMDKASVLGDAIKYVKQLQESVKVLEEQTKKRTVESVVFVKKSQLVSSDDDTSSCDENFDSRCSDEPLPQIEARVSEKDVLIRIHCENQKGYVVKVLSELENLQLSVVNSSVLPFGKSTLDITIVAQMDDEFNMTVTDLAKSLRVALLKFMPDVERTSALRLKVRTSSVLCLLTPDWPATSFQANLDQSWCFPSRASIRQENLSPASPGRRETVLEACAVVREGEKPSPA

>mrna07355.1-v1.0-hybrid-32_Fv

MEISSIGGLTELGMEDSFFINQWHMNSLDELSMLPLAAAFGENLQHSHCHPILNPKTSTNTSQNSIDRPMKQLKPNNWSPPNPQFAALPNLLSFVNSCNTNQVGVAKPKEEKTLCSKSINNHLPSDVLVSQASFGNQNSLLKAGHGTNRISTNTRLSQTQVHIMAERKRREKLSQQFIALSAMVPGLKKTDKASVLGDAIKYIKQLQEKLKILEEQTRVKKMETVVSVKRSHLIANGDNNSTVSFEETLPQIEARFCDNNVLIRVHCEKRKGVVEKTIAEVEKLQLKLINSSVMTFGSSVVDVTIVAQMEVEFCLTVKDLVKNLRSAFDLFM

>FANhyb_rscf00000397.1.g00002.1-33_Fa

MEDPTFLNQYEMNFHLDYSLEDLNFRSLSAESYSSYPDFTPPQNDVDERPAKQPKNNHSNWNSCTTTHDHKITVPRASSSASSHLISFDNSNSSAYGSLDCTTTVKPKNEVQGSGGNLNSIPTLISQGNSYDPQTCSPNRAYGQGIKRAATVTRSPLHAQDHVLAERKRREKLSQRFIALSALVPGLKKMDKASVLGDAIKYVKQLQERMKILEEQAAKKTVESVVFVKRTQYSAEDDNSSSDENFDSCSDQPLPEIEARVSDKEVLIRIHCEKKKGCLANILDEIERLNLTILNSSFLPFGNSTLDITVVSQMDVEYSMTVKELVRSKALYTYISLMHERREHYSSSRAVYCHGWFFAARLRICWRRVFPVIFCVVDHVQLPSSDDKAAGFSL

>FANhyb_rscf00000397.1.g00003.1-33_Fa

MDASSAKWFSDLGMDDYNFIQEGLTAQDIAIALGGNFKQSFSNESYSSYSTLTTQNTPTTTLSGGSSINETNSQTSFERPAKLVKTNNWNSGITEHVSPKPSSNSSSSHILSFENANLNSSPPSKPHQKFSNGFDTLKPKEEAPSQICMQFENQGYGSNKRPYSMTRTPSHAQDHIMAERKRREKLSQRFIALSAIVPGLKKMDKASVLGDAIKYVKQLQESVKVLEEQTKKRTVESVVFVKKSQLVSSDDDTSSCDENFDSRCSDEPLPQIEARVSEKDVLIRIHCENQKGYVVKVLSELENLQLSVVNSSVLPFGKSTLDITIIAQMDDEFNMTVTDLAKSLRVALLKFM

>FANhyb_rscf00000468.1.g00011.1-33_Fa

MEISSIGGLTELGMEDSFFINQWHMNSLDELSMLPLAAAFGENLQHSHCHPILNPKTSTNTSQNSIDRPMKQLKPNNWSPPNPQFASLPNLLSFINSCNTNQVGVAKPKEEKTLCSKSINNHLPSDVLVSQASSIGNQNSLLKAGHGTNRISTNTRLSQTQVHIMAERKRREKLSQQFIALSAMVPGLKKTDKASVLGDAIKYIKQLQEKLKILEEQTRVKKMETVVSVKRSHLIANGDNNSTVSFEETLPQIEARFCDNNVLIRVHCEKRKGVVEKTIAEVEKLQLKLINSSVMTFGSSVVDVTIVAQMEVEFCLTVKDLVKNLRSAFDLFM

>MDP0000124672-34_Md

MDMISSAKWVSDFEIEDPXFINQYEMSSRDYSLDELNFLSFSSESYSSYPNFTPKADNFSKASIENLHQMYGTHERPAKQPKNNTNSWNSCSTDHIITANASSSSSSHLISFESSNSSPPTTSQQYYGLDCRVIXPKNEVEYSNGKLNPRALISQGIYDPQTCSPKHGQGIKRAATVTKSPLDAQDHVLAERKRRENLSQRFIALSALLPGLKKTDKASVLGDAIKYVKHLQELTKMLEKQAAKKTVEAVVFVKRTQYSADDDISSSDENSESCSNQPLPEIEARVSDKEVLIRIHSEKTKGSLASILSEIEKLDLTIVHSCALPLGNSTLDITVVAQMDVEFRMTVKDLVKNLRQALLKLVGPEI

>MDP0000159571-34_Md

MEISSRGGLSELGMEDPFFINQWQYMNSLDELGMLPLAAAIGDNLQHSHFHPTFNLKTPIDNSHTGIDRPAKQLKTDGWTSCKTDHLVLNPQVASSPNILSFVNSNHGNQMVVLKPKEEAAAVCSKSTNTLPSDILLSQGSFGNQNCLFKASQGTNRNSTNPRLSATQDHIIAERKRREKLSQRFVALSALIPGLKKMDKASVLGDSIKYIKQLQDKVKTLEEHTRNKNMESVVFVKKTQLFANGDKTSSNENNSTGPFDATLPEIEARFCDNNVLIRIHCEKRKGVVEKTISEVEKLQLKFINSSVMTFGSCALDVTIIAQMEAEFSLSVQDLVKNLRTAFNLFM

>MDP0000170202-34_Md

MEDPTFINQYEMSSLDYSFEELIFPSLSSDSYCSNPNFTSKATAATHNFSKAFIENPHQTGTQGRSAKQPKNTNSWKSCSTDHIIAAKASSSSSSHLISFENSDSSPPTTSQQYYGLDCKVIKPKNEVEYSNGKLNPSALASQCSYDTQICSPKHGQGIKRAATVTRSPLHAQDHVLAERKRREKLSQRFIALSALIPGLKKMDKASVLGDAIKYVKHLQERTKVLEEQAAKKTGEAVVFVKRMQYSADDDISSSDENSESCSDQPLPEIEARVSDKEVLIRIHCEKTKGCLTSILSEIEKLGLTIVHSCALPFGNSTLDITVVAQMDVEFRVAGKHLIKNLRHALFKLTFGGNTSHCTHAASKALFCRRNGHGSGSGGNDTDGEGGDGDGSNNGGSMMVVTIVGCGSX

>MDP0000174149-34_Md

MEISSRGGLSELGMEDPFFINQWQYMNSLDELGMLPLAAAFGENLQHSQFHPIFNLKTPIDNSHTAIDRPTKQLKTDGWTSCKTDHLVSNPQVASLPPNILSFVNSHHANQMPVLKPKGEGAAVCSKSTNTLPSDILLSQGSFGNRNYSSNGISTTTRLSQTQDHIIAERKRREKLSQRFIALSAIVPGLKKMDKASVLGDSIKYIKQLQDKVKTLEEQTRNKNMESVVFVKKTQLFANSDSSCSDENSSTGPFDTTLPEIEARFCDNNVLIRIHCEKRKGIVEKTIAEVEKLQLKFINSSVMTFGSCALDVTIIAQMEAEFSLSVKDLVKNLRAXFELFM

>MDP0000291132-34_Md

MEFQKTNSGFRFGMSNNHRRDLRFSAPPPTVLAAKTRVLTRKSPRADRGSWLGFGSGRIQCFKEVQGRRGMMTTTNKVYMVVLVSKLVLTLIEFKFNTYLGMDYSFIPQCHLNGLDEGFNAHDIGTALGENFKHSFSESYSSYSTLTTKNTTTTTTIASSGSSINETSHGTSFESPAKLLKTSSWNSSITENVSPKPCSSSSQILSFENLNSPLPSNPQKFCNKFEPALKSKDEAPSQINMQFSHAQDYEAKGCSQGTKRPCPTSRTPSHAQDHIMAERKRREKLSQRFMALSAIVPGLKKMDKASVLGDAIKHVKQLQERVKVLEERSKKRTVESVVFVKKSQLSADDDTSSCDENFDGCSPDEAALPEIEARVSEKDVLIRIHCEKQKGVVVKILSEIEKLQLSVVNSSIFPFGASTLDITIMSQMDDGFNMTVKDLARKLRVALLTFIGTPRVYASPIHSYSTTLAEGNLTYEVTDLTAEYLNNDEVIISATKAPASKPRRERKSCEDKKVIKNVDSIQVSHHVYAMLLGNQILTSNPDLAMQANVVFLPLLVFMKTIPSSFILTFFLPASGYPSHTIMRLQRCCFLRPIRVWQLFGATCPIIVSVFNKTMSTALGTTRPGGKVCLVGMDHGVLDPFTPAAARKCVAFSMGL

>MDP0000318216-34_Md

MASXVHXRARNLDVRSXQRLGYSSKVQVPKRFGLGVAAKYSPRVAVGHEPRELGRGKIKAQEAQRXAGKLGQAXGGLGASCSSKLTLGRNLVVECSSTTKKKKTKKSVGFXQATPPRMAXRGKEVVRTPEPYIIGPVHSIFLIFLLFSHRLDVERVRIPIKDYNCKTLPSWRTGKTRTWIVFTGRKEMEDPTFINQYAMSSLDYSLDELIFSPFSSESYSSYPNFTSKTAAVTHNFSKAFIENPHQTGSQERPAKQPKNTTSWKSCSTDHIIAAKASXSSSSHLISFENSDSSPPTTSQQYNGLDCKVIKPKNEVEYSNGKLNLPALISQGSYDTQTCSPKHGXGIKRAATVTRSPLHAQDHVLAERKRREKLSQRFIALSALLPGLKKMDKASVLGDAIKYVKHLQERTKVLEEQAAKKTGEAVVFVKRMQYSADDDISSSDENSESCSDQPLPEIEARVSDKEVLIRIHCEKTKGCLTSILSEIEKLGLTIVHSCALPFGNSTLDITVVAQMDXQFSMTVKDLVRASHVPTNESGATEIDAWASHNGRLGFQRLLQRSLTTWPNL

>MDP0000391743-34_Md

MLKIMFLRRESAEKISASVASIRDSKLPIFTKLQTDKASVLGDAIKYVKHLQELTKMLEKQAAKKTVEAVVFVKRTQYSADDDISSSDENSESCSNQPLPEIEARVSDKEVLIRIHSEKTKGSLASILSEIEKLDLTIVHSCALPLGNSTLDITVVAQMDVEFRMTVKDLVKNLRQALLKLVGPEI

>MDP0000502440-34_Md

MSSSTPSSTTGVGRQRHXVGIXXDEQERWGQMMSLIEPLXAMSXRLRLRGMDYSFIPQCHLNSLDDGFNAHDIGTALGENFKQSFSSESYSSYSTLTTKNTPTTTTIASSGGSSINETSHGTSFERPAKLLKTSSWNSIITENVSPKPCSSSSQILSFENLNSPLPSKPQKFCNKFEPALKPKDEXPSQINMRFSHAQDYEAKGSSQGTKRPCPTSRTPXSHAQDHIMAERKRREKLSQRFIALSAIVPGLKKMDKASVLGDAIKHVKELQERVKVLEERSKKRTVESVVFVKKSQLSADDDTSSCDENFDGCGPDEAALPEIEARVSEKDVLIRIHCEKQKGVVVKILSEIEKLQLSVVNSSVLPFGASTLDITIMSQMDDGFNMTVKDLARKLRGALLTFM

>XP_008236173.1-35_Pm

MAISSIGGMSELGMEDPYFINQWHLNSLDEVSMLPLAAAFGENFNQSHFHPNFNLKTSMDSSHSGIDRPMKQLKTDGWSSCKTDQQGSNPQVASSPNILSFVNSHSTNQMGVLKPKEEAAVCSKSNNGLPSDILLSQSSFGNQSYLFKASQGTKGVNTNTRLSTTQDHIIAERKRREKLSQRFIALSAMVPGLKKMDKASVLGDAIKYIKQLQDKVKTLEEQTRKKNMESVVFVKKTQLFANDDNSSSEENNSSGPFDETLPEIEARFCDNNVMIRIHCEKRKGVVEKTIAEVEKLQLKFINSSVLTFGGCALDVTIIAQMEVEFSLSVKELVKNLRSAFDMFM

>XP_008236876.1-35_Pm

MDISSAKWFSELGIDDYNFIPQCDLNALDEGFTAQDIATALGENFKQSLSSESYSSYPTLTAQNTKTTTTTTTTLSGGSSINKTSQTSFERPAKQLKTSNWNSGITEHVSPKPSSSSSQILSFESSSSPSSKPQHFCNNFDSTLKPKDEAPSQINMQFSPLISKSSIKDCSQGTKRPYSITRTPSHAQEHIMAERKRREKLSERFIALSAIVPGLKKMDKASVLGDAIKHVKQLQDRVKVLEEQTKKRTVESVVFVKKSQLSADDGTSSCDESFDGHSDEASLPEIEAKVSETDVLIRIHCEKQKGFVVKILSEVEKLQLSVVNSSILPFGNSALDITIIAQMEDQFNMSVKDLARNLRVALLKFM

>XP_008236887.1-35_Pm

MDMISSAKWVSDLEMEDPTFIHQYEMNSLDYSLDDLNFQSFSSESYSSYPNFTPKATHNFNNASIETSHQAGTHERPAKQPKNHTTWNPCTSDHTFMAKAASSSSSHLISFDNSNSSPPTSSQQFYGNLDNTMKPKNEVEYSNGKLNLTTLISQGSYDPQTCSPRHEQGIKRAATVTRSPLHAQDHVLAERKRREKLSQRFIALSALVPGLKKMDKASVLGDAIKYVKHLQERTRMLEEQAVKKTVEAVVFVKRTQYSADDDISSSDENFESCSDQPLPEIEARVSDKEVLIRVHCEKTKGCLAKILSEIESLDLTIVNSSVLPFGNSTLDITVIAQMDAEFSMTVKDLVKNLRQSLLKFV

>Prupe.6G211900.1.p-36_Pp

MDISSAKWFSELGIDDYNFIPQCDLNTLDEGFTAQDIATALGENFKQSLSSESYSSYPTLTTQNTKTTTTTLSGGSSINKTSQTSFERPAKQLKTSNWNSGITEHVSPKPSSSSSQILSFESSSSPSSKPQHFCNNFDSTLKPKDEAPSQINMQFSPLISKSSIKDCSQGTKRPYSITRTPSHAQEHIMAERKRREKLSERFIALSAIVPGLKKMDKASVLGDAIKHVKQLQERVKVLEEQTKKRTVESVVFVKKSQLSADDGTSSCGESFDAHSDEASLPEIEAKVSETDVLIRIHCEKQKGFVVKILSEVEKLQLSVVNSSVLPFGNSALDITIIAQMEDEFNMSVKDLARNLRGALLKFM

>Prupe.6G212000.1.p-36_Pp

MDMISSAKWVSDLEMEDPTFIHQYEMNSLDYSLDDLNFQSFSSESYSSYPNFTPKATHNFSNASIETPQQAGTHERPAKQPKNHTTWNPCTTDHTIMAKAASSSSSHLISFDNSNSSPPTSSQQFYGTLDNTMKPKNEVEYSNGKLNLTTLISQGSYDPQTCSPKHGQGIKRAATVTRSPLHAQDHVLAERKRREKLSQRFIALSALVPGLKKMDKASVLGDAIKYVKHLQERTKMLEEKAVKKTVEAVVFVKRTQYSADDDISSSDENFESSSDQPLPEIEARVSDKEVLIRVHCEKTKGCLAKILSEIESLDLTIVNSSVLPFGNSTLDITVIAQMDAEFSMTVKDLVKNLRQSLLKFV

>Prupe.8G157500.1.p-36_Pp

MEISSIGGMSELGMEDPYFINQWHMNSLDEVSMLPLAAPFGENFNQSHFHPNFNLKTSMDSCHSGIDRPMKQLKTDGWSSCKTDQHGSNPQVASSPNILSFVNSNSTNQMGVLKPKEEAAVCSKSNNSLPSDILLSQSSFGNQSYLFKASQGTKRVNTNTRLSTTQDHIIAERKRREKLSQRFIALSAMVPGLKKMDKASVLGDAIKYIKQLQDKVKTLEEQTRKKNMESVVFVKKTQLFANDDNSSSEENNSSGPFEETLPEIEARFCDNNVMIRIHCEKRKGVVEKTIAEVEKLQLKFINSSVLTFGGCALDVTIIAQMEVEFSLSVKELVKNLRSAFDMFM

>Pbr022522.1-37_Pb

MEISSRGGSSELGMEDPFFINQWQYMNSLDELGMLPLAAVFGENLQHSQFHPIFNLKTPIDNAHTAIDRPTKQLKTDGWTSGKTDHLVSNPQVVSLPPNIFSFVNSHHANQMPVLKPKEEAAAVCSKSTNTLPSDILLSQGSFENRNYSSNGISTTTRLSQTQDHIIAERKRREKLSQRFIALSAIVPGLKKMDKASVLGDSIKYIKQLQDKVKTLEEQTRNKSMESVVFVKKTQLFANGNNPSSDENGSTGPFDTTLPEIEARFCDNNVLIRIHCEKRKGIVEKTIAEVEKLQLKFINSSVMTFGSCALDVTIIAQMEAEFSLSVKDLVKNLRAAFELFM

>Pbr022523.1-37_Pb

MEISSRGGSSELGMEDPFFINQWQYMNSLDELGMLPLAAVFGENLQHSQFHPIFNLKTPIDNAHTAIDRPTKQLKTDGWTSGKTDHLVSNPQVVSLPPNIFSFVNSHHANQMPVLKPKEEAAAVCSKSTNTLPSDILLSQGSFENRNYSSNGISTTTRLSQTQDHIIAERKRREKLSQRFIALSAIVPGLKKMDKASVLGDSIKYIKQLQDKVKTLEEQTRNKSMESVVFVKKTQLFANGNNPSSDENGSTGPFDTTLPEIEARFCDNNVLIRIHCEKRKGIVEKTIAEVEKLQLKFINSSVMTFGSCALDVTIIAQMEAEFSLSVKDLVKNLRAAFELFM

>Pbr024262.1-37_Pb

MEDPTFINQYEMSFLDYSLDELNFQSFSSESYSSYPNFTPKADNFSKASIENLHQMSGTHERPAKQPKNNTSSWNSCSTDHIITANASSSSSSHLISFENCNSSPPTTSQQYYGLDCRVIKPKNEVEYSNGKLNPPALISQGIYDPQTCSPKHGQGIKRAATVTRSPLDAQDHVLAERKRRENLSQRFIALSALLPGLKKTDKASVLGDAIKYVKHLQELTKMLEKQAAKKTVEAVVFVKRTQYSADDDISSSDENSESCSNQPLPEIEARVSDKEVLIRIHSEKTKGCLASILSEIEKLDLTIVHSCALPLGNSTLDITVVAQMDVEFSMTVKDLVKNLRQALLKLVGPEI

>Pbr024263.1-37_Pb

MEDPTFINQYEMSFLDYSLDELNFQSFSSESYSSYPNFTPKADNFSKASIENLHQMSGTHERPAKQPKNNTSSWNSCSTDHIITANASSSSSSHLISFENCNSSPPTTSQQYYGLDCRVIKPKNEVEYSNGKLNPPALISQGIYDPQTCSPKHGQGIKRAATVTRSPLDAQDHVLAERKRRENLSQRFIALSALLPGLKKTDKASVLGDAIKYVKHLQELTKMLEKQAAKKTVEAVVFVKRTQYSADDDISSSDENSESCSNQPLPEIEARVSDKEVLIRIHSEKTKGCLASILSEIEKLDLTIVHSCALPLGNSTLDITVVAQMDVEFSMTVKDLVKNLRQALLKLVGPEI

>Pbr040909.1-37_Pb

MEISSRGGLSELGMEDPFFINQWQYMNSLDELGMLPLAAEIGDNLQHSHFHPTFNFKTPIDNSHTGIDRPAKQLKTDGWPSCKTDHLVSNPQVASSPNILSFVNSNHANQMAVLKPKEEAAAVCSKSTNSLPSDLLLSQGSFGNQNCLFKASQGTNRNSTNPRLSATQDHIIAERKRREKLSQRFIALSAIIPGLKKMDKASVLGDSIKYIKQLQDKVKTLEEQTRNKNMESVVFVKKTQLFANGDKTSSDENNSTGPFDATLPEIEARFCDNNVLIRIHCEKRKGVVEKTISEVEKLQLKFINSSVMTFGSCALDVTIIAQMEAEFSLSVQDLVKNLRTAFNLFM

>Pbr040914.1-37_Pb

MEISSRGGLSELGMEDPFFINQWQYMNSLDELGMLPLAAEIGDNLQHSHFHPTFNFKTPIDNSHTGIDRPAKQLKTDGWPSCKTDHLVSNPQVASSPNILSFVNSNHANQMAVLKPKEEAAAVCSKSTNSLPSDLLLSQGSFGNQNCLFKASQGTNRNSTNPRLSATQDHIIAERKRREKLSQRFIALSAIIPGLKKMDKASVLGDSIKYIKQLQDKVKTLEEQTRNKNMESVVFVKKTQLFANGDKTSSDENNSTGPFDATLPEIEARFCDNNVLIRIHCEKRKGVVEKTISEVEKLQLKFINSSVMTFGSCALDVTIIAQMEAEFSLSVQDLVKNLRTAFNLFM

>Pbr041632.1-37_Pb

MEDPTFINQYEMNSLDYSFDELIFSSLSSESYSSNPNFTSKPAAATHNFSKAFVENPHQTGTQDRPAKQPKNTTSWKSCSTDPIIAAKASSSSSSHLISFENSDSSPPTTSQQYYGLDCKVIKPKNEVEYSNGKLNLSALVSQGSYDTQTCSPKHGQGIKRAATVTRSPLHAQDHVLAERKRREKLSQRFIALSALLPGLRKMDKASVLGDAIKYVNHLQERTKVLEDQVAKKTGEAVVFVKRMQYSADDDISSSDENFESCSDQPLPEIEARVSDKEVLIRIHCEKTKGCLTSILSEIEKLGLTIVHSCALPFGNSTLDITVVAQMDVEFSMTGKHLVKNLRHALLKLV

>Pbr041633.1-37_Pb

MDYSFIPQCHLNSIDEGFNAHDIGTALGENFEESFSSESYSSYSTLTTKNTTTTMTIASSGGSSINETSHGTSFERPAKLLMTSSWNSSITENVSPKPCSSTSQILAFENLNSPLPSNPQKFCYKFEPALKSKDEAPSKINMQFSHAPDYEAKGCSQGTKRPCPTSRTPSHAQDHIMAERKRREKLSQQFIALSAIVPGLKKMDKASVLGDAIKHAKQLQERVKVLEERSKKRTVESVVFVKKSQLSADDDTSSCDENFDGCGPDESALPEIEARVSEKDVLIRIHCEKQKGDVVKILSEIEKLQLSVVNSSIFPFGASTLDITITSQMDDGFNMTVKDLARKLRVALLTFM

>AT2G22750.2_AtbHLH018-38_At

MNSLVGDVPQSLSSLDDTTTCYNLDASCNKSLVEERPSKILKTTHISPNLHPFSSSNPPPPKHQPSSRILSFEKTGLHVMNHNSPNLIFSPKDEEIGLPEHKKAELIIRGTKRAQSLTRSQSNAQDHILAERKRREKLTQRFVALSALIPGLKKMDKASVLGDAIKHIKYLQESVKEYEEQKKEKTMESVVLVKKSSLVLDENHQPSSSSSSDGNRNSSSSNLPEIEVRVSGKDVLIKILCEKQKGNVIKIMGEIEKLGLSITNSNVLPFGPTFDISIIAQKNNNFDMKIEDVVKNLSFGLSKLT

>AT2G22760.1_AtbHLH019-38_At

MDEDFFLPDFSLVDIDFDFNIYEENNLSPDESLSNSRRADQSSKFDHQMHFECLREKPKAAVKPMMKINNKQQLISFDFSSNVISSPAAEEIIMDKLVGRGTKRKTCSHGTRSPVLAKEHVLAERKRREKLSEKFIALSALLPGLKKADKVTILDDAISRMKQLQEQLRTLKEEKEATRQMESMILVKKSKVFFDEEPNLSCSPSVHIEFDQALPEIEAKISQNDILIRILCEKSKGCMINILNTIENFQLRIENSIVLPFGDSTLDITVLAQMDKDFSMSILKDLVRNLRLAMV

>AT2G22770.1_AtbHLH020-38_At

MDDSSFMDLMIDTDEYLIDDWESDFPICGETNTNPGSESGSGTGFELLAERPTKQMKTNNNMNSTSSSPSSSSSSGSRTSQVISFGSPDTKTNPVETSLNFSNQVSMDQKVGSKRKDCVNNGGRREPHLLKEHVLAERKRRQKLNERLIALSALLPGLKKTDKATVLEDAIKHLKQLQERVKKLEEERVVTKKMDQSIILVKRSQVYLDDDSSSYSSTCSAASPLSSSSDEVSIFKQTMPMIEARVSDRDLLIRVHCEKNKGCMIKILSSLEKFRLEVVNSFTLPFGNSTLVITILTKMDNKFSRPVEEVVKNIRVALAE

>AT4G37850.1_AtbHLH025-38_At

MSILSTRWFSEQEIEENSIIQQFHMNSIVGEVQEAQYIFPHSFTTNNDPSYDDLIEMKPPKILETTYISPSSHLPPNSKPHHIHRHSSSRILSFEDYGSNDMEHEYSPTYLNSIFSPKLEAQVQPHQKSDEFNRKGTKRAQPFSRNQSNAQDHIIAERKRREKLTQRFVALSALVPGLKKMDKASVLGDALKHIKYLQERVGELEEQKKERRLESMVLVKKSKLILDDNNQSFSSSCEDGFSDLDLPEIEVRFSDEDVLIKILCEKQKGHLAKIMAEIEKLHILITNSSVLNFGPTLDITIIAKKESDFDMTLMDVVKSLRSALSNFI

>cra_locus_1072_iso_2-39_Cr

ILNMEAPSTSWFSDLGLLENPLVSDECDMFDFLNQEYLSAAALGKDSQALCASSAMNIEAPQQWPDNNTYNSFENLTEMLPEQVNLEDAAAKMAPIRKKNSSSRGREPSQTYDHIIAERKRREQLSRQFVALSGIVPGLKKTDKTSVLGDTINYLKHLQEKVKKLEEKTTKKSMESVVLAKKSQLPFEDEDKGSSHEQSFPHIEAKVFDKNILIRIQCLKHKGVLLNALSSIMKFNLAVTKINATPFGRWALEITIIAQMETEFSLTIQDLIESLQSALKKNR

>cra_locus_12822_iso_1_CrBIS1-39_Cr

MTMMMTMDNSVNSWFSDLGMEDPFSSDQYDITDFLNEDFAALGEDLQAFTPTAESDSSNNFINIPTSNSSNTLCALATELPSVVAEIPTTITATTTTKKRKSNSSTNQNVPNARRAARTPIVLTFGNTTAETNPNKHSLSPDINDDSLISTENLTSQGNLEEAVAAAKSTKLNKKTGGRVRPASQTYDHIIAERKRREQLSQHFVALSAIVPGLKKMDKTSVLGDAITYLKHMQERVKTLEEQTTKQTMESVVLVKKSQVLVEDEGSSDEIDQDQSSSQLPEIEAKVCDKTILLRVHCEKNKRVLINILSQLEKLNLVVTNTSVSAFGSLALDITIIVEMEKESSINMKELIQTLRSAVMRANLED

>cra_locus_1467_iso_1-39_Cr

LPTIFPRNYSFSQARRILGKLSKMDLLGAHEWFSQLEVTDHDEWQMKSNSNNFNTNSSAAYYCSSNESIQAASHQAQKKSPLLTSPHHHQKKKMDFISFGGNNNNNNYSNSSAAAKSYGKACNNIVKTEDTMMSLNGDLNFSSSIHQYYETILEEEAGVNNKRVCNRTALQAQDHVLAERKRREKLTERIVALSTMVPGLKKLDKASVLGGAIKYLEQLQERIKMLEAEKRELLEEQSANNNNNNLNNNNMSSLISKRPRISVSSDEISSSAGEISYCSTPGGQPPLSSPEIEVRISEKTDVLVRVYCKKRNGVIKDILSEIEKLHLSIMSSSVIPFGTSILNITVIAQMDGDSVCLSAQNIASNLRTAIQKLMQ

>cra_locus_19483_iso_1-39_Cr

MEVANFRSLAELGIDHGFIHQTWPLNSFDELSSLSMAASAFGEHYQMQQHSSDHHQIFSQKHSLELSENGVLDRPVKQQKTNSWSSSNSHLSNPQIDGSPNYHVVNSGYTDHHHQSMVMKPKEEIISMNFPRDLMVSQGSIGNQNYVFKTNQGAKSLSPKARLTQAQDHIMAERKRREKLSQRFIQLSALVPGLKKMDKASVLGDAIKYLKQLQDKVKNLEEQTKKRTMESVVFVKKYELQFDTDNSSSNENFSGEPINEPLPEIEARCSDKDVLIRIHCDRRKGILDKTVSEIEKLHLSVINSSVMTFGSLTLDITIIAQMEEEFNMSMKDLVQNLRVTLKKFL

>cra_locus_2845_iso_5_CrBIS2-39_Cr

MTDNSGISNWFTELGMEDPNNFLMINNDDECDVMEFLNEDICAATVGQDYYFQISPTFSLNTTSTLYPSSSSTPMDILDQSPPFMLDDDIDETMNRRPAKQLKSTSNNNQNNQNPSTIHDSFDAQMSTPYLLTFGNPNSPEIINPPHHQQHHQPNATLNLNPSDEDVQVSEVFNSQSSSYGNLIEEEAAAPKSSKPTSKKSGGRVRPASQTYDHIIAERKRREILSQRFMALSTLVPGLKKMDKTSVLGDAIKYLKYLQERVQILEDQAAKQTMESVVMVKKSHVFIQEEEDDEEGSSDDQITSDGGSSEEHPLPEIEVKVCNKTLLLRIHCEKQKGVLIKLLNEIERLNLGVTNINVAPFGSLALDITIIAEMEKEYNMTTVQVIKNLRSVLLNSPPMAD

>AUR62005875-RA-40_Cq

METDPNNPSLINNPYQQSETMEPSFEEQLAATLGNDFPYYPIFATTVPAFPEGNIHQSPRENKKETTMKQAVSSAGAKRKRRPSQVQDHIMAERKRRQLLRHMFISLSAILPGLKKIDKTTVLGEAIKHMKELQEKVKVLESVIAKRTMESVVAVVKKSKLIIDNGSSDDNVSSSTVDDYCGNGDDSFPEIQVKVMGKTLLLRVLCEKQKGILAKLFAEIDNQDLTITNFSVVPFESLALHVTIVAQMESGFNKNVRDLVRILGNAIASKWLISNCMR

>AUR62005877-RA-40_Cq

MSSIMTMDAWLAELEMEMDTIPSLINHPSHQSGTMEAAFDEQLVATLADDFPYNLYDTSEATSPQGDFECNNVIEQFISTPPISETSENNTDNNNNNDKNDSDNIVSSDVQESQPSKTKEETKKKQGVSRAGAKRKRQPSQVQDHILAERKRRELLSQLFISLSAIVPGLKKVDKTSVLGEAIKHMKELQEKVKVLESVIAKRTMESVVAVVKKSKLIIDNGSSDDNVSSSTVDDHCGSGSNDDGDSSGDSLPEVEVKILGKSLLLRVYCGQQKDILVKLFAEIDKHHLSITNFSVIPFENLAQDITIVAQVMIPQLPSTS

>AUR62010677-RA-40_Cq

MTSTMTMDAWLAELEIDMDSIDSLINPYYQSVMDMESTFDEQLAATFFDTASPQGDIECDVIEQYMSTPPISDLSENHDDNNNKNDRDNIASSDVQESQLSKTKQKTKKQAVSDGPKRKRQPSQVQDHIIAERKRRELLSQMFISLSAIVPGLKKIDKISILGEAIKHMKELQEKVKVIENEVAKRTVESVVLVKGIVDDNVSSSSTMEDYCGHGSSDDDGGNSNGSETFSEIDVKVLGKTVLLRIYCEKRKEILAKLLAEIDKHHLVITNFSVIPFENLALNITIVAQMEGGFKKNVRDFATTLRNALH

>AUR62014013-RA-40_Cq

MSSTMTMDAWLAELEMEMDTIPSLINSHCEQSGTMEAAAFDEQLAATLADNFPYSLYDTSDATSPQGDFECNNVIEQFISTPLISETSENHTDVEEMSQPPKTKEETKKKQAVSSGGAKRKRQPSQVQDHIIAERKRRELLSQLFISLSAIVPGLKKIDKTSVLGEAIKHMKELQEKVKVLESVIAKRTMESVVLVKKSNLMFDEGSSDDNASSSTTVDDYCRSSNNDDGDSSSDSLPEVEVKILGKSLLLRVYCGQQKDILVKLFAEIDKHHLSITNFSVIPFENLAQDITIVAKMERGFNKNVKDFVRTLHNALHLASCQRFDFNQNEYSS

>AUR62017204-RA_CqTSARL1-40_Cq

MDAWLAELEIDMGTIDSLINNPYDEQSVMDMESSFEEQLAATFFDTASPQGDIECNVIEQYISSPPIPDISENHDDNNDNINNKNDNDNNIVSSNVQERQPSKTKQKTKKQAMSTSHAPKRKRQPSQVQDHIIAERKRRELLSQMFISLSAILPGLKKIDKTSILGEAIKHLKELQEKVKVLESEVAKRTVESVVLFKGIANDNVSSSSTVEDYGGNGSSGSSDDDGGNNNSSDSFLEIEVKVLEKTILLRVYCEKRKEILAKLFAEVDKHHLVITNFSVIPFENLALNITIVAQMERGFNKNVREFATTLRNALH

>AUR62017206-RA_CqTSARL2-40_Cq

MTSTMTMDAWLAELEIDMDPVDSLIDPYHQSVMDMESTFDEQLAAAFFDTASPQVDIECNVIEQYISTPPISDISENHDDNNNNNNNNESENIVSSDVQESQPSKTKQKTKKQAVSTSHAPKRKRQPSQVQDHIIAERKRRELLSQMFISLSAILPGLKKIDKTSVLGEAIRHMKELQEKVKVLESEVAKRIVESVVLVNGTADDNVSSSSTMDDYLKVLGKTVLLRVYCEKRKEILAKLFAEVDKHHLVITNFSVIPFENVALTVTIVAQMEKGFNKNVRDFATTLRNALH

>AUR62027064-RA-40_Cq

MEASMITGFTDMELDDFSFIDHLLQVNSSSLEQIDLFGPPLLPSYEEDTYKKDTNYNQLQVKRSPQIDHHTIVEEKPLIKQHITNNWNSSYEIDHAFTSQDDDYSNMNMLSFSSSSSSINSQNCIPKATQVAKKSSAALASLSQQPKDHIIAERKRREKLNQRFIALTATIPGLKKMDKASVLGDAIKYVKQLEKQVKKLEEVTKTKTIESAVLVKRSRVFEEEENDQFIKEGPLYPEIEAKFSNKDVLIRINCEKKNGVVEKIISHIENLHLVVINSSTLVFGCSSIDATIIAQMDAEFSMTSKDLVRHLHAALKRLL

>AUR62032870-RA-40_Cq

MSQPSKTKQAVSSGGAKRKRQPSQVQDHIIAERKRRELLSQLFISLSAIVPGLKKIDKTSVLGEAIKHMKELQEKVKVLESVIAKRTMESVVLVKKSKLMVDEGSSDNNVSSSTTVDDYCRSSNNDDGDSSSDSLPEVEVKILGNSLLLRVYCGQQKDILVKLFAEIDKHHLSITNFSVIPFENLAQDITIVAK
