# Supplementary material for: Insights into the diversification of subclade IVa bHLH transcription factors in Fabaceae
Source: BMC Plant Biol. 2021 Feb 23;21:109. doi: 10.1186/s12870-021-02887-w (PMC7901066; doi:10.1186/s12870-021-02887-w)
Supplement: Supplementary file 3 — Additional file 3 Fig. S1. Phylogenetic tree of subclade IIIf and IVa bHLH proteins in Glycine max and Arabidopsis thaliana. Fig. S2. Detailed phylogenetic tree of subclade IVa bHLHs in fabids. Fig. S3. Predicted domains of subclade IVa bHLH proteins identified using MEME. Fig. S4. Expression patterns of TSAR1 orthologues. Data were retrieved from Lotus Base, Soybean eFP browser, and Medicago eFP browser. Fig. S5. Biosynthesis pathways for aglycones of soyasaponins and hemolytic saponins from M. truncatula. This figure shows representative aglycones of soyasaponins and hemolytic saponins. Cytochrome P450 monooxygenases have been found to oxidise different carbon positions of the β-amyrin backbone [cytochrome P450 enzymes (positions to be oxidised), *characterised in soybean]. Although the soyasaponin pathway is common among Fabaceae, only Medicago spp. acquired the hemolytic pathway. Fig. S6. Expression of LjCYP93E1 and LjbHLH032. Data retrieved from Lotus Base. Lj1g3v3555800: LjCYP93E1; Lj0g3v0292969: LjbHLH032. [file 12870_2021_2887_MOESM3_ESM.pdf]

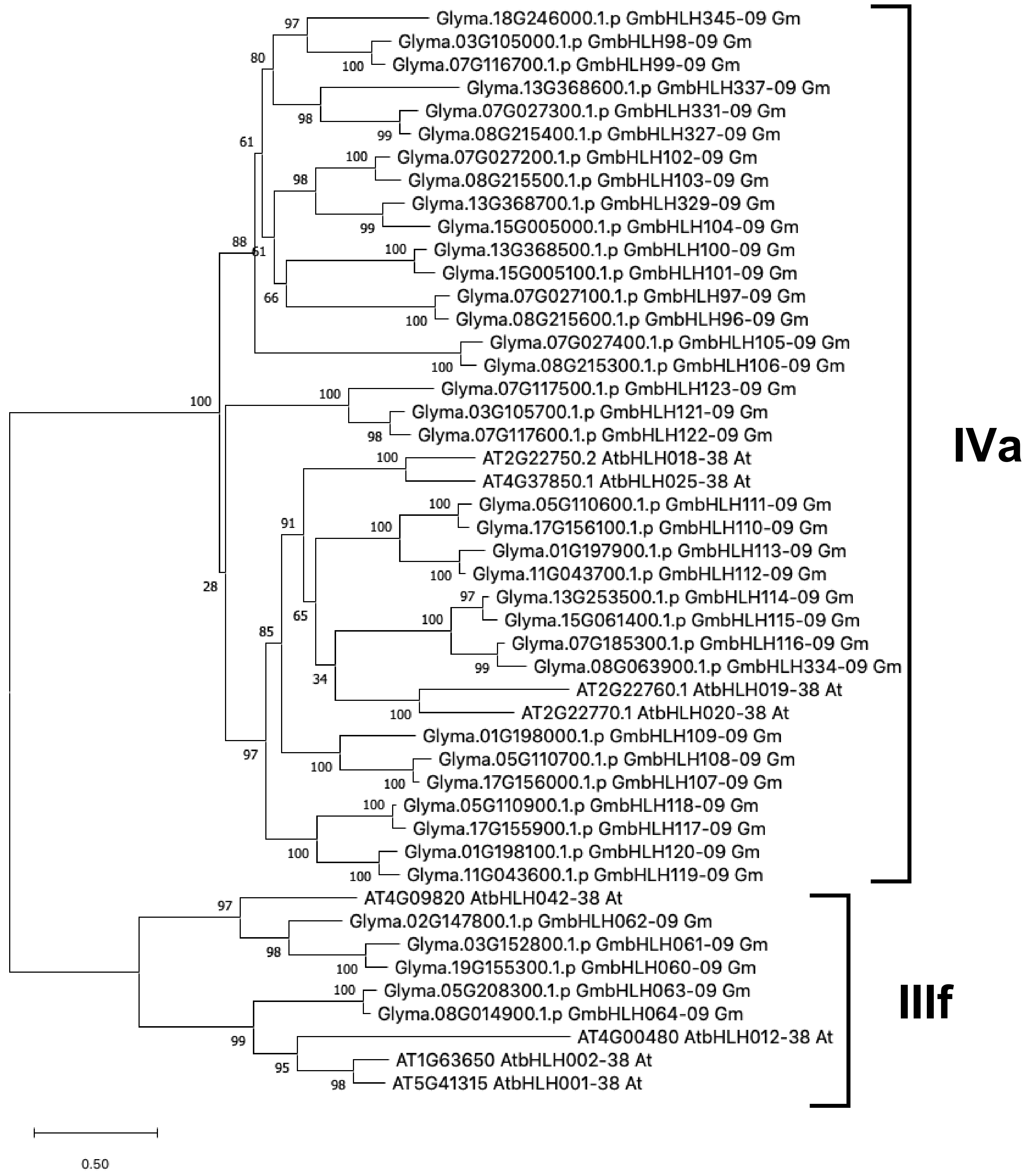

Fig. S1. Phylogenetic tree of subclade IIIf and IVa bHLH proteins in *Glycine max* and *Arabidopsis thaliana*.

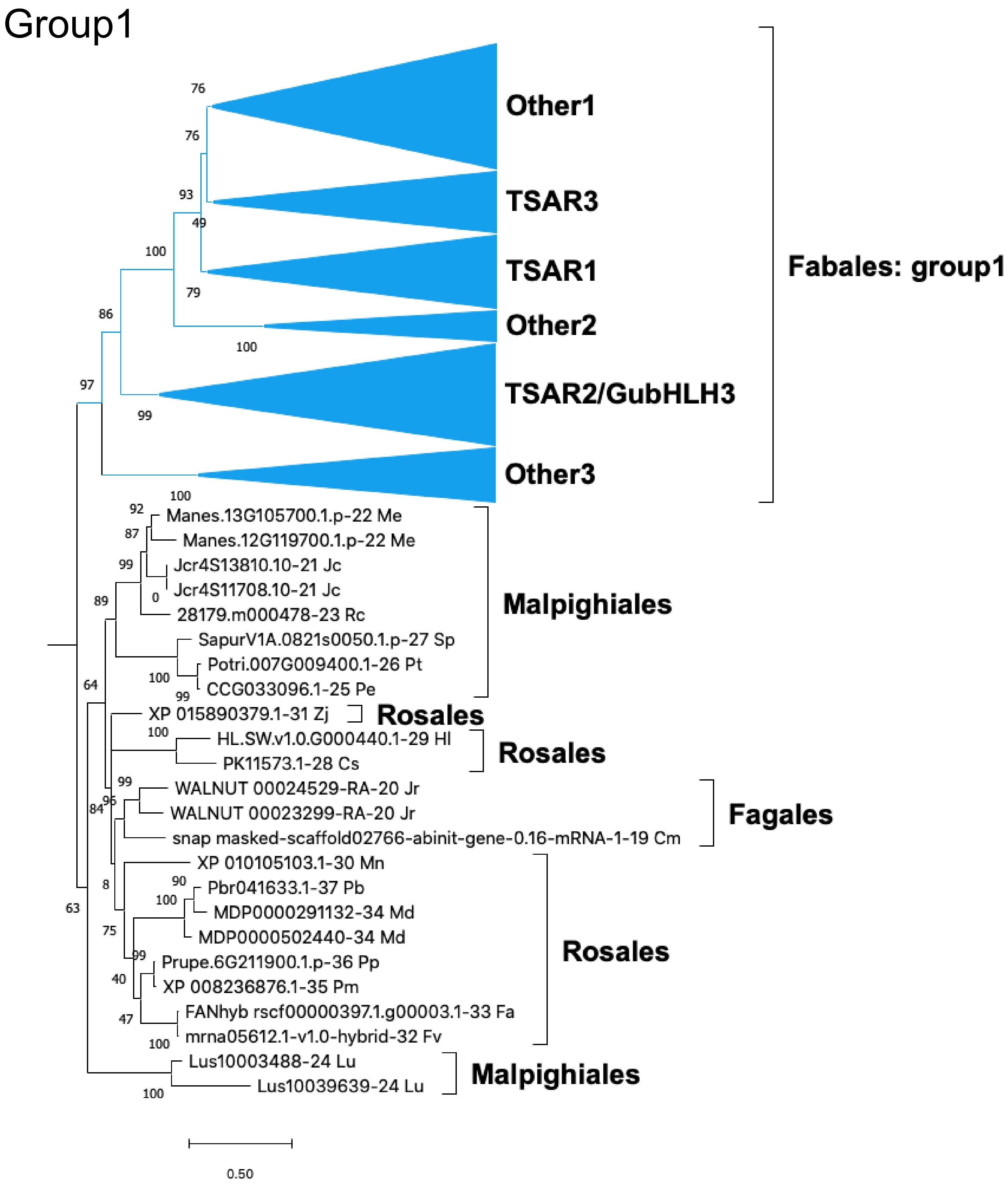

Fig. S2. Detailed phylogenetic tree of subclade IVa bHLHs in fabids.

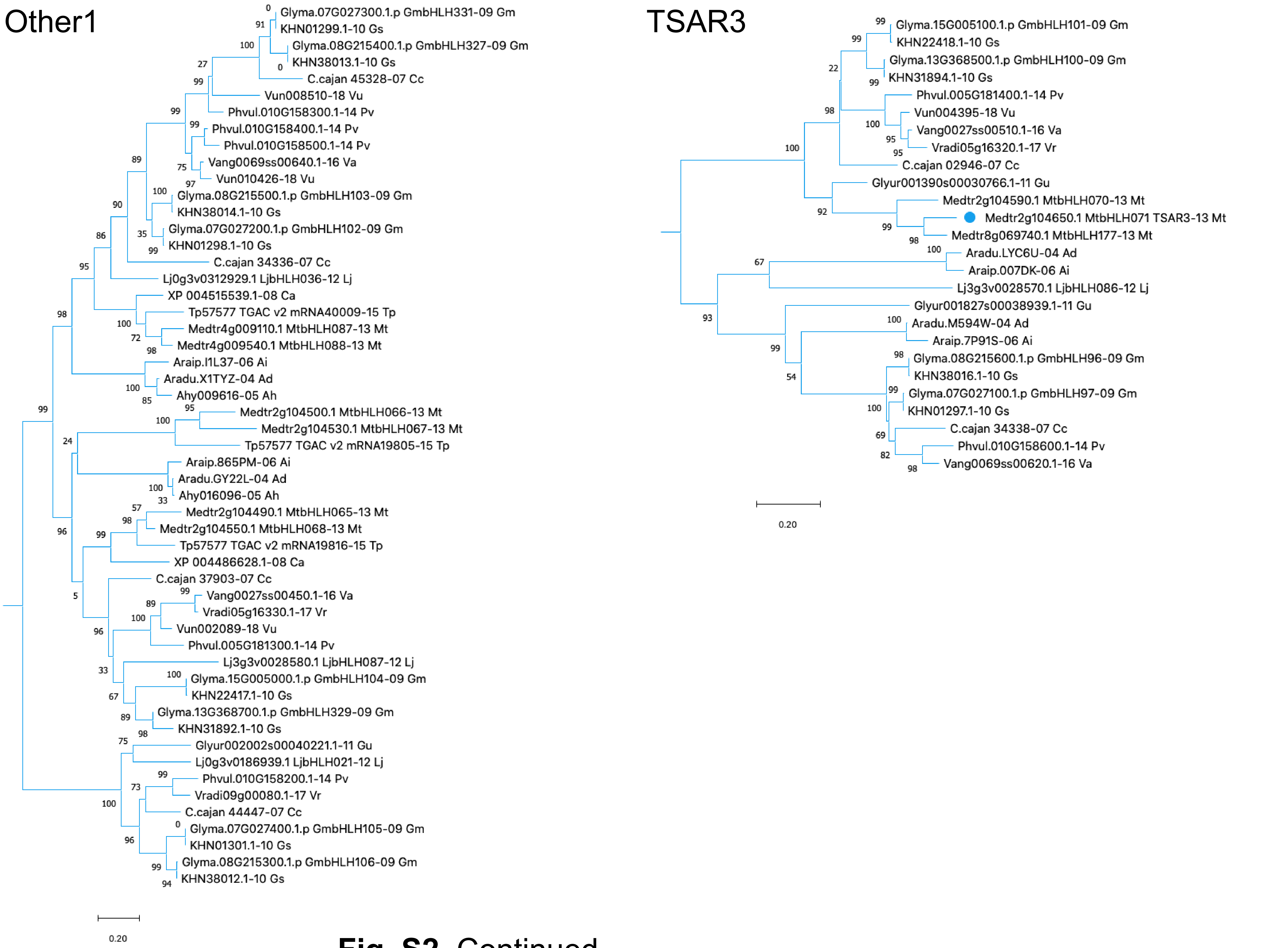

Fig. S2. Continued

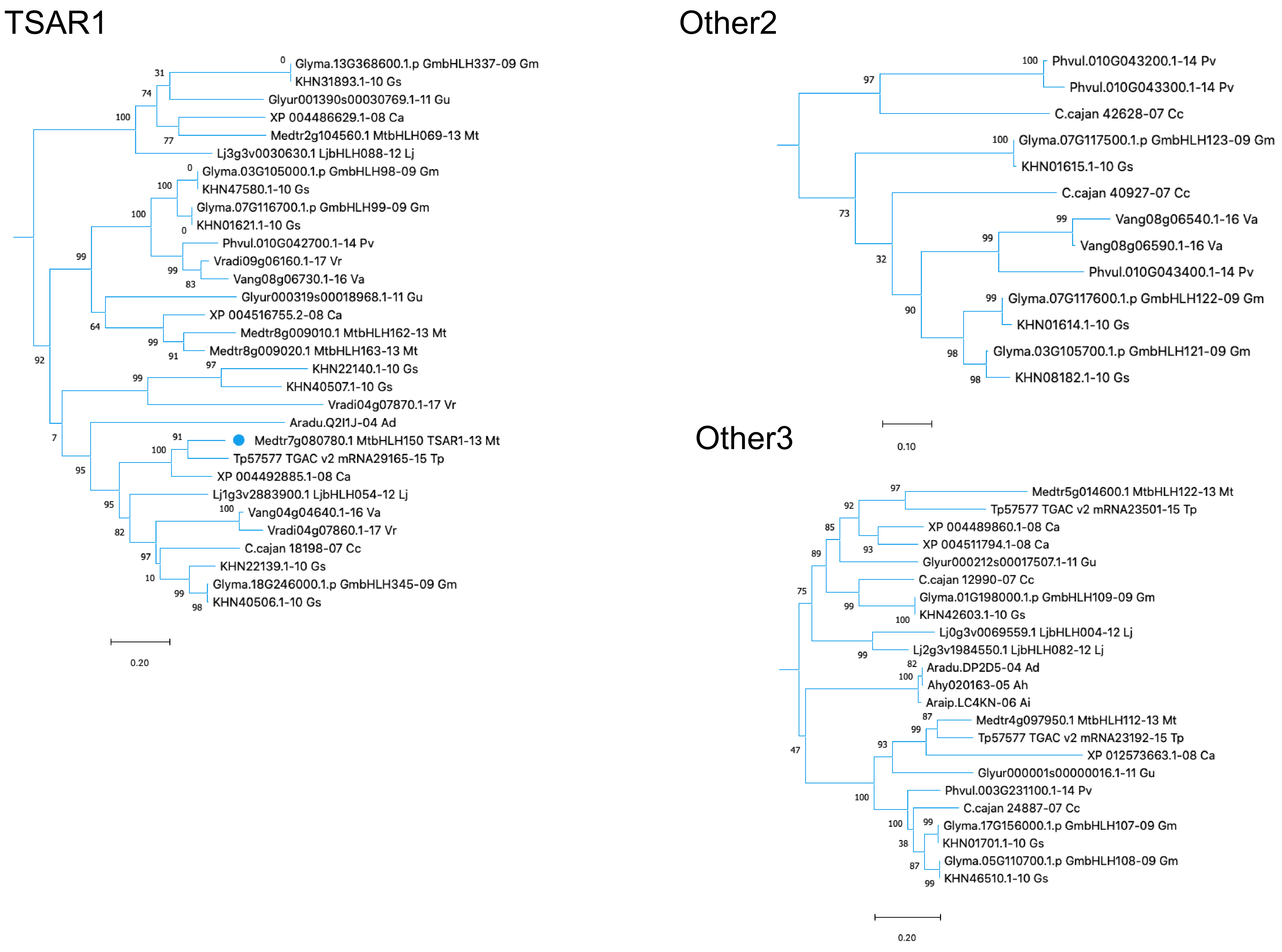

Fig. S2. Continued

TSAR2/GubHLH3

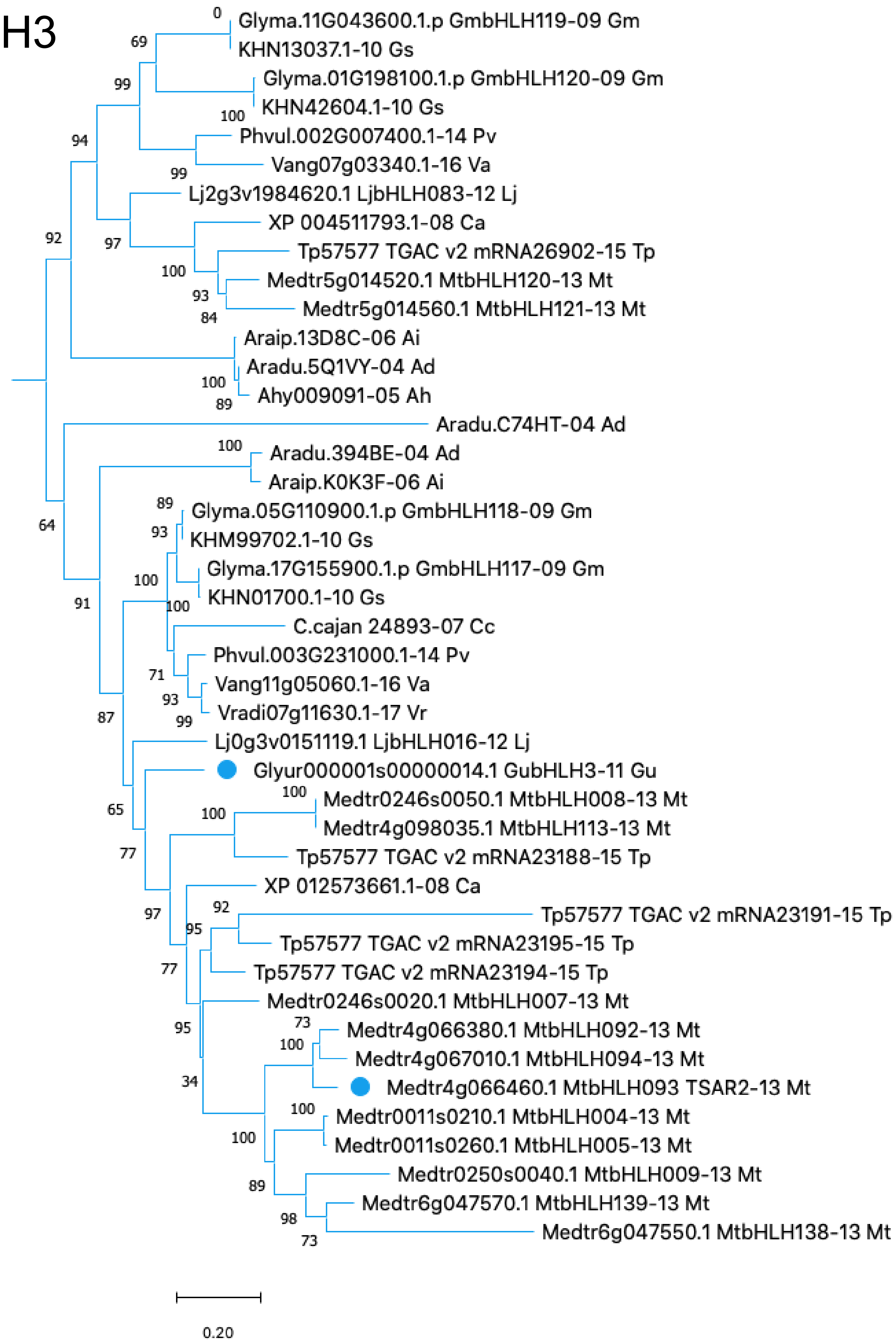

Fig. S2. Continued

Group2

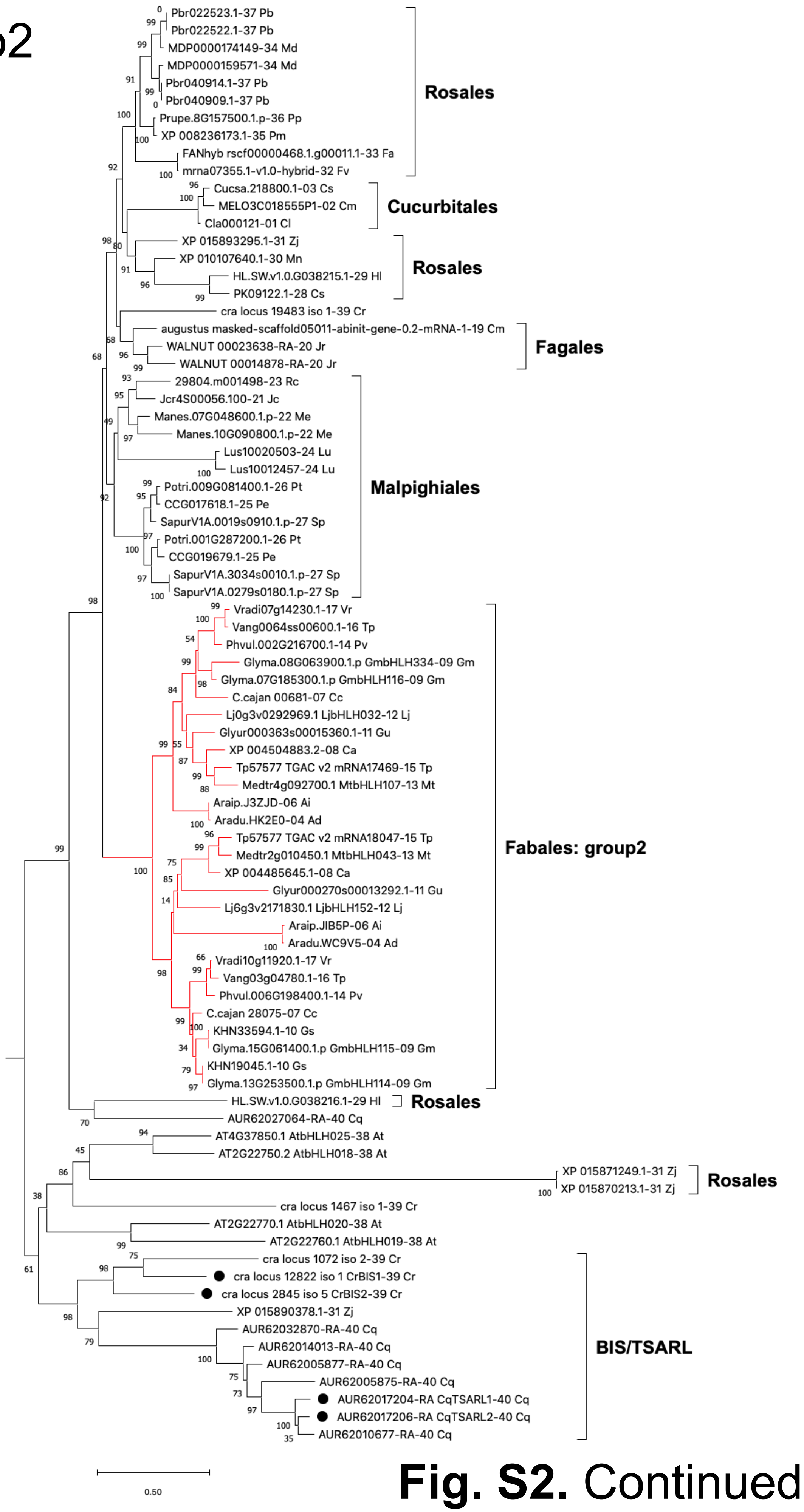

Fig. S2. Continued

Group3

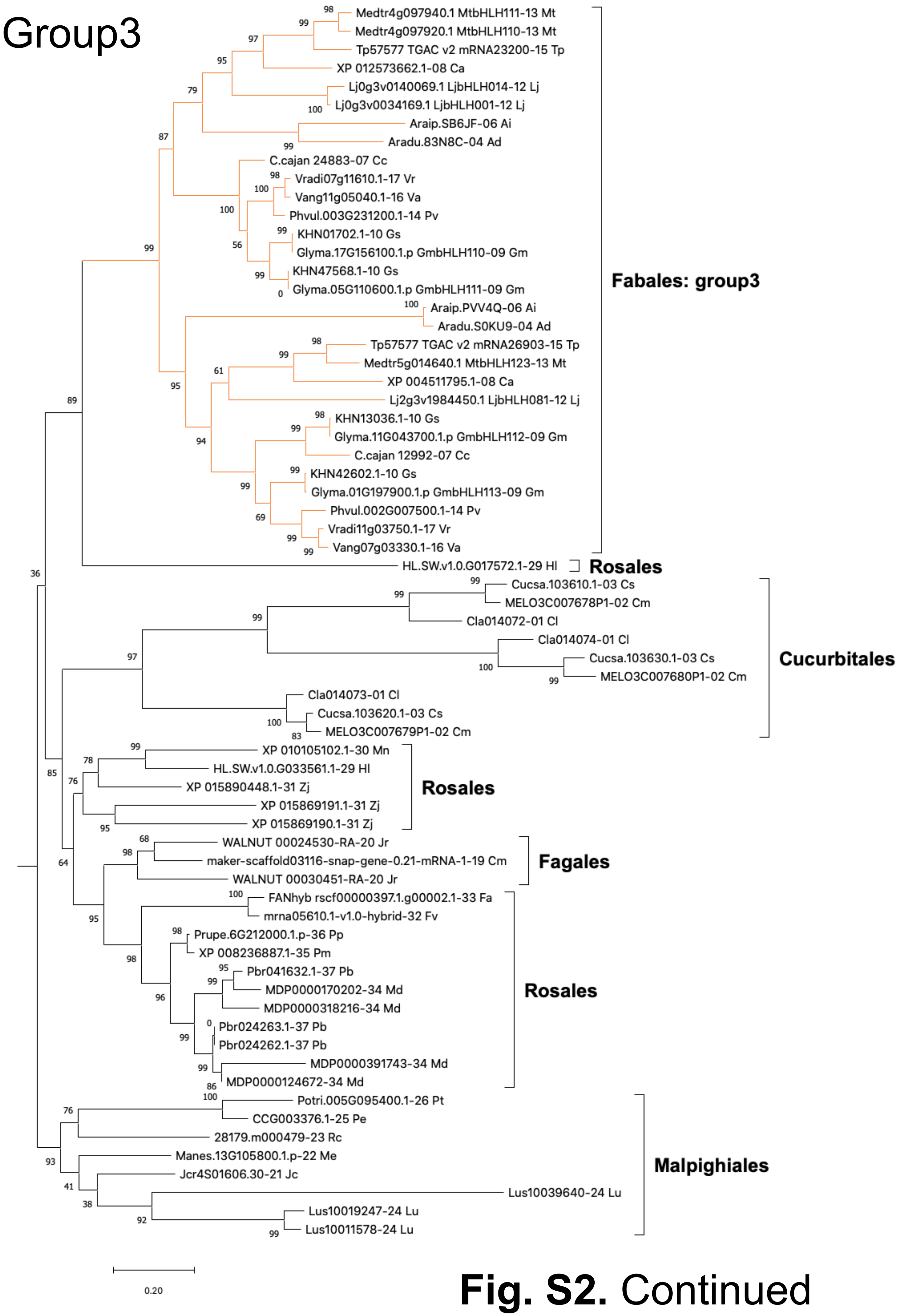

Fig. S2. Continued

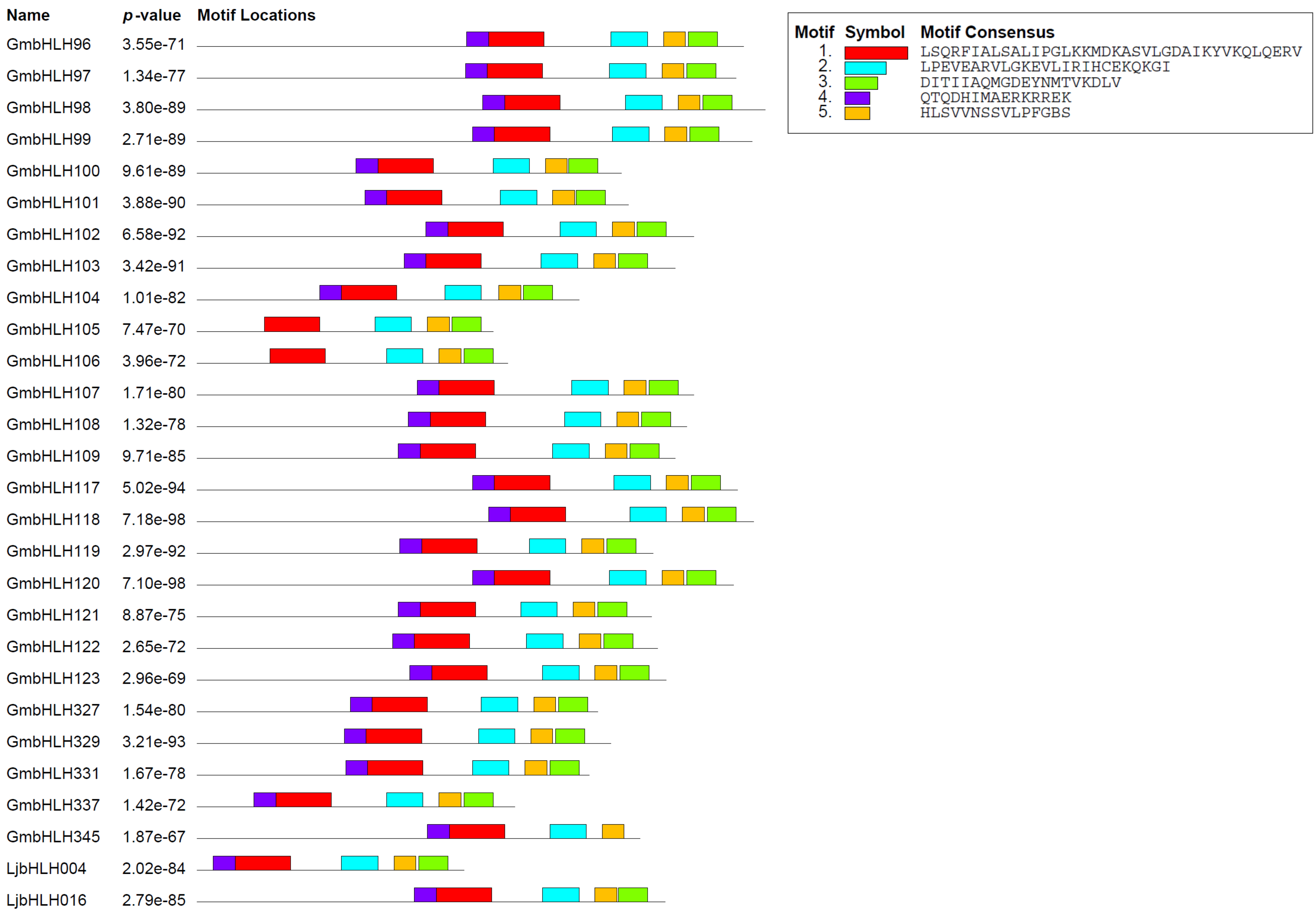

Fig. S3. Predicted domains of subclade IVa bHLH proteins identified using MEME.

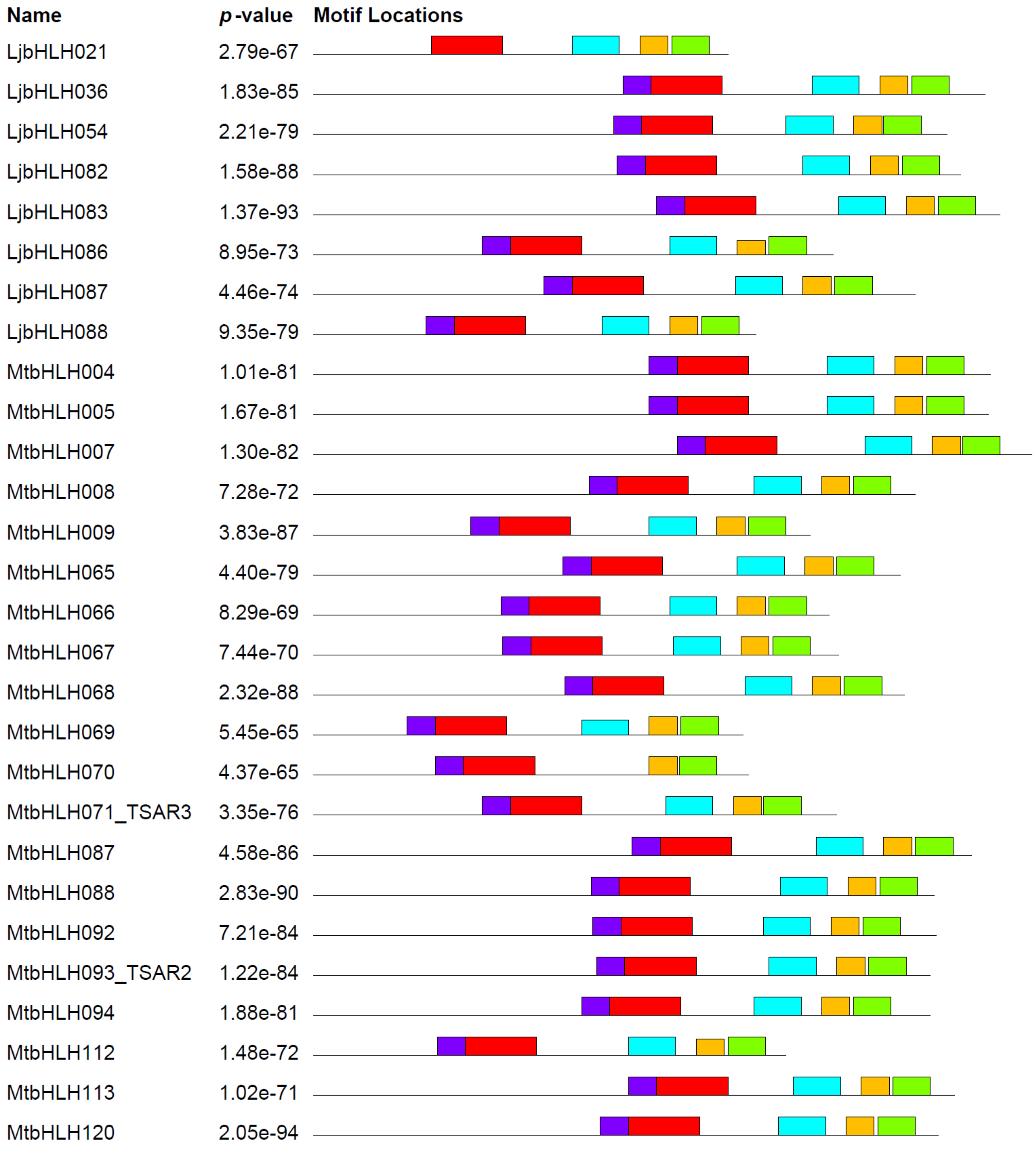

Fig. S3. Continued

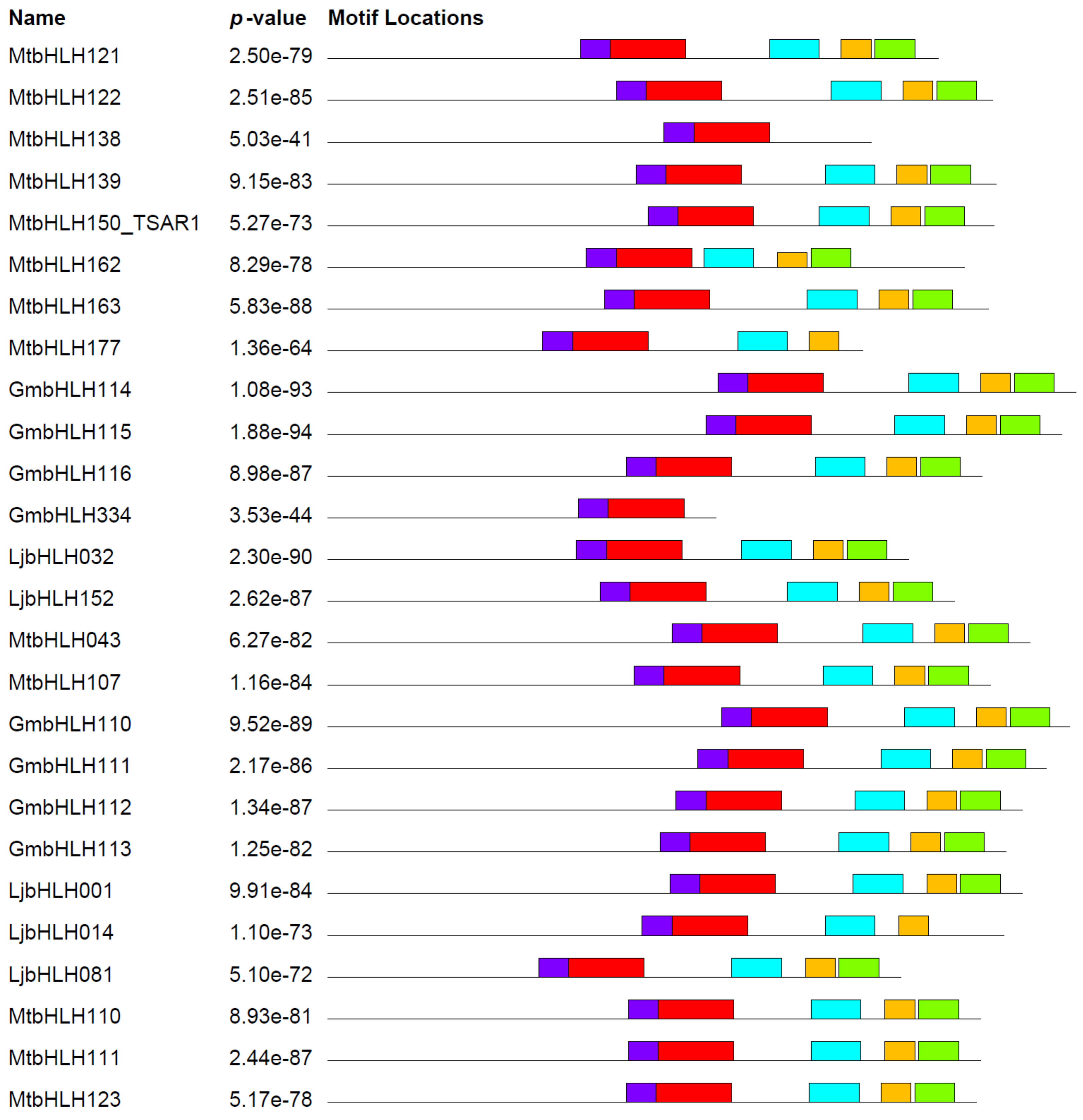

Fig. S3. Continued

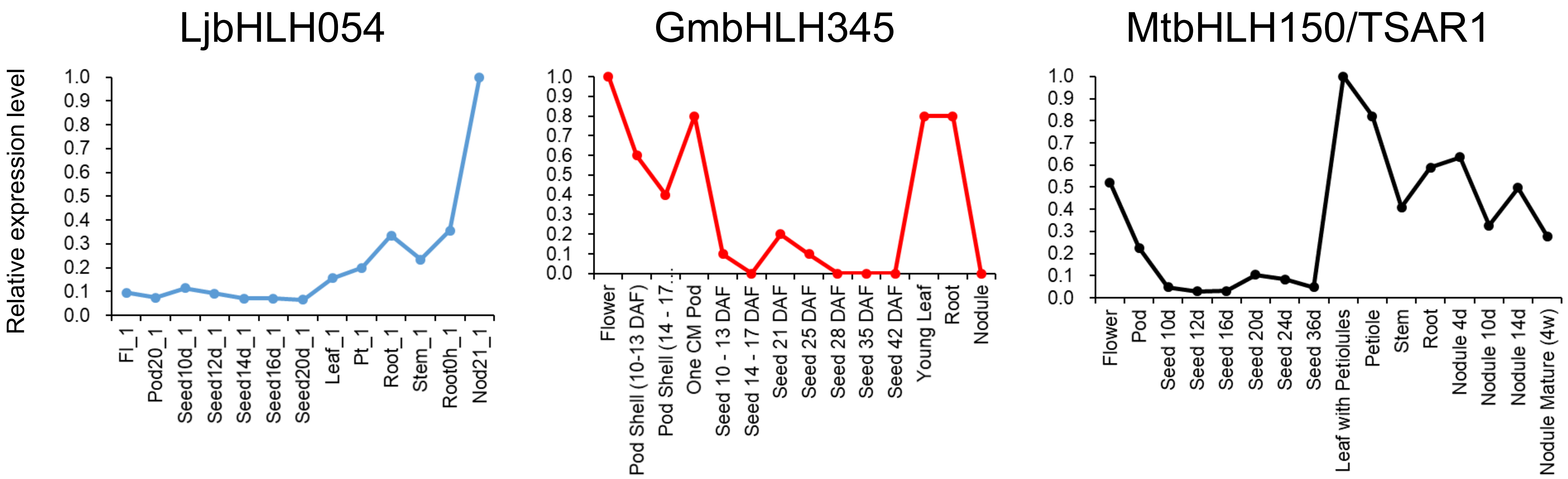

Fig. S4. Expression patterns of *TSAR1* orthologues. Data were retrieved from *Lotus* Base, Soybean eFP browser, and Medicago eFP browser.

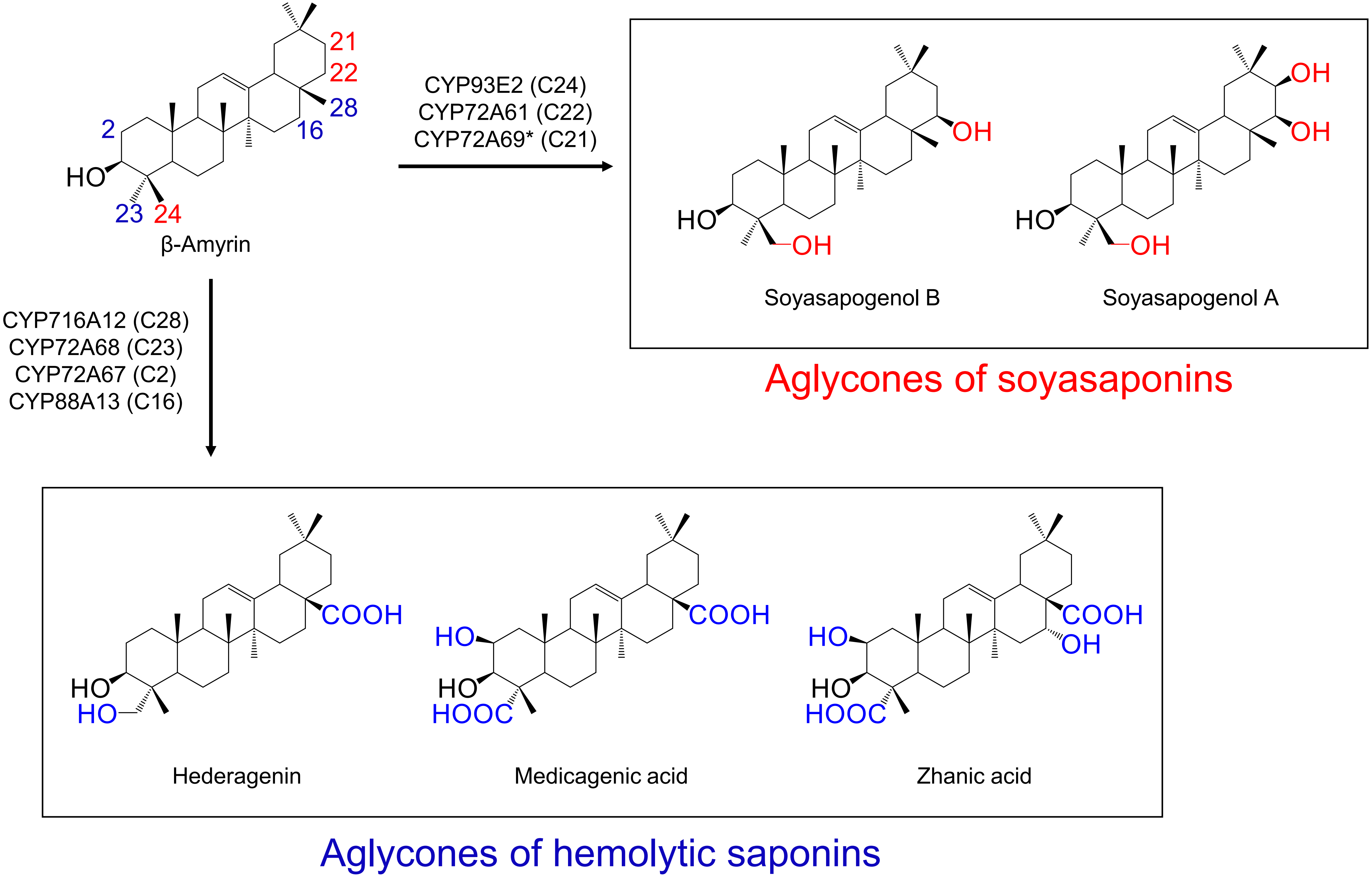

Fig. S5. Biosynthesis pathways for aglycones of soyasaponins and hemolytic saponins from *M. truncatula*. This figure shows representative aglycones of soyasaponins and hemolytic saponins. Cytochrome P450 monooxygenases have been found to oxidise different carbon positions of the β-amyrin backbone [cytochrome P450 enzymes (positions to be oxidised), *characterised in soybean]. Although the soyasaponin pathway is common among Fabaceae, only Medicago spp. acquired the hemolytic pathway.

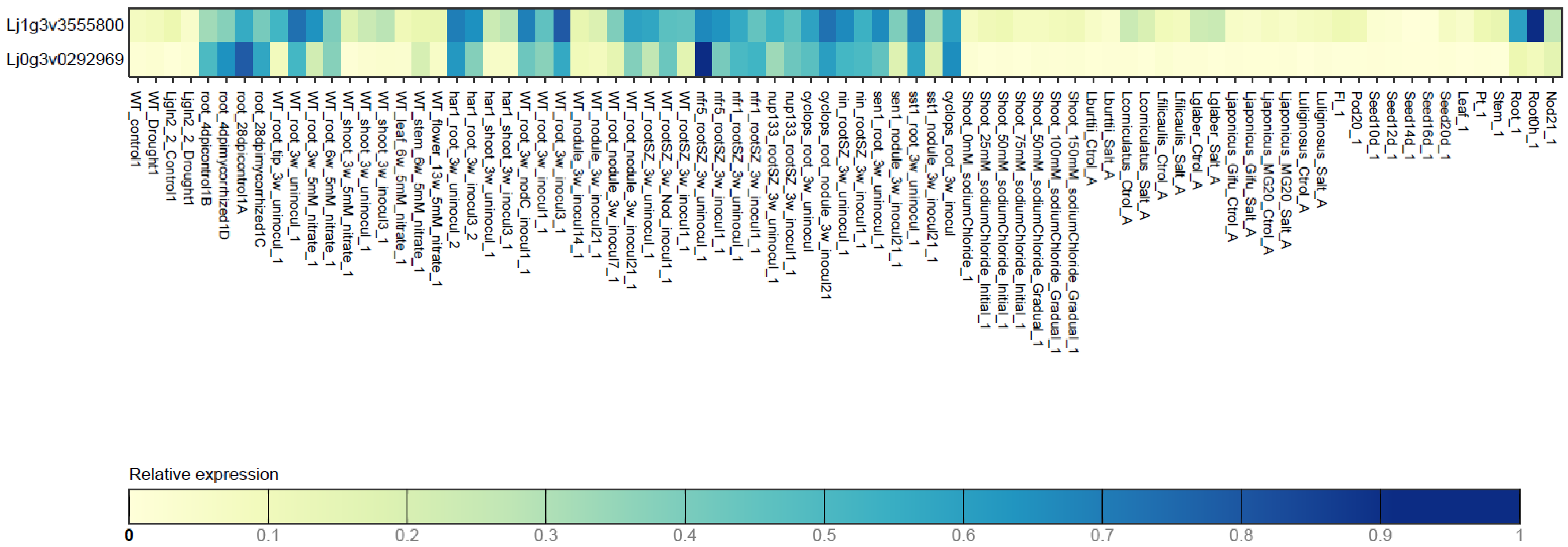

Fig. S6. Expression of *LjCYP93E1* and *LjbHLH032*. Data retrieved from *Lotus* Base. *Lj1g3v3555800*: *LjCYP93E1*; *Lj0g3v0292969*: *LjbHLH032*.
